# Ancient genomes provide evidence of demographic shift to Slavic-associated groups in Moravia

**DOI:** 10.1186/s13059-025-03700-9

**Published:** 2025-09-03

**Authors:** Ilektra Schulz, Denisa Zlámalová, Carlos S Reyna-Blanco, Sam Morris, Guido Alberto Gnecchi-Ruscone, Raphael Eckel, Renáta Přichystalová, Pavlína Ingrová, Petr Dresler, Luca Traverso, Garrett Hellenthal, Jiří Macháček, Daniel Wegmann, Zuzana Hofmanová

**Affiliations:** 1 Department of Biology, University of Fribourg, Fribourg, 1700 Switzerland; 2 Swiss Institute of Bioinformatics, Fribourg, 1700 Switzerland; 3 Department of Archaeology and Museology, Masaryk University, Brno, Czech Republic; 4 UCL Genetics Institute, University College London, London, UK; 5 Department of Archaeogenetics, Max Planck Institute for Evolutionary Anthropology, Leipzig, Germany; 6 Research Department of Genetics, Evolution & Environment, University College London, London, UK; 7 Senckenberg Centre for Human Evolution and Palaeoenvironment, University of Tübingen, Tübingen, Germany

## Abstract

**Background:**

The Slavs are a major ethnolinguistic group of Europe, yet the process that led to their formation remains disputed. As of the sixth century CE, people supposedly belonging to the Slavs populated the space between the Avar Khaganate in the Carpathian Basin, the Merovingian Frankish Empire to the West and the Balkan Peninsula to the South. Proposed theories to explain those events are, however, conceptually incompatible, as some invoke major population movements while others stress the continuity of local populations.

**Results:**

We report high-quality genomic data of 18 individuals from two nearby burial sites in South Moravia that span from the fifth to the tenth century CE, during which the region became the core of the ninth century Slavic principality. In contrast to existing data, the individuals reported here can be directly connected to an Early-Slavic-associated culture and include the earliest known inhumation associated with any such culture.

**Conclusions:**

The data indicates a strong genetic shift incompatible with local continuity between the fifth and seventh century, supporting the notion that the Slavic expansion in South Moravia was driven by population movement.

**Supplementary Information:**

The online version contains supplementary material available at 10.1186/s13059-025-03700-9.

## Main text

### Background

The first historical sources mentioning Slavs describe their attacks on the Byzantine Empire at the beginning of the sixth century [1, 2]. In Central Europe, written sources (*Chronicle of Fredegar*) document their presence no later than the first half of the seventh century [3, 4]. However, while linking archeological finds to identities is challenging, archeological evidence suggests that the Slavs were present in large parts of Central Europe several generations earlier [5–7]. Whether this process was driven by the movement of people has long been debated (e.g., [8–19], see Additional File 1: Section 1).

A particular challenge in addressing the question of the emergence of the Slavs is that the earliest historically documented usage of a Slavic language dates to the ninth century [20], and thus postdates their alleged emergence by centuries. These earliest Church Slavonic texts include the *Life of St. Constantine* about St. Constantine who introduced the Glagolitic alphabet to transcribe the Slavic language. They originated in the early Slavic Moravian principality located in modern day Czech Republic and Slovakia, whose rulers and inhabitants spoke a Slavic language according to interpretations of written sources (e.g., see in *The Annales* of Fulda/East Frankish chronicles, AD 846: Sclavos Marganses/Moravian Slavs, [21]), and are often linked with the Middle Hillfort period (800–950 AD, [22]). In Moravia, the Early Hillfort period (680–800 AD) continued the Prague-Korchak-culture period (classically dated to 540–680, [22–24]), characterized in its earliest phases by Prague-type pottery that emerged across vast territories of Europe as of the sixth or early seventh century CE [5, 7, 25]. Linking the spread of the Prague-type pottery with the spread of the Slavic language [26, 27], some archeologists and historians have explained the emergence of Slavs by invoking the migration of Slavic-speaking people from a supposed homeland, usually placed outside of Central Europe in present-day Ukraine and Belarus [28–30].

However, neither the self-described ethnicity nor the languages spoken by the early bearers of the Prague-Korchak culture are known as these people left no texts or inscriptions [10], except for the runic bone from Lány, which is surprisingly associated with a Germanic- and not a Slavic-speaking population [31]. Archeological finds cannot therefore serve as the only or main indicator of Slavic ethnicity [9, 32] and whether there is concrete historical evidence that supports a Slavic expansion is disputed [11], leaving room for alternative theories about their emergence that stress population continuity [3]. Curta [3], for instance, argues that the supposed Slavic ethnicity could be a mere social construction in that earlier groups in Central Europe, including both indigenous and migrant such as the Germanic speaking Lombards [33], might have undergone a process of assimilation, effectively transitioning into Slavic identities due to economical and social changes. And as a newer collective action theory emphasizes, this process of assimilation may have occurred through bottom-up social strategies [6, 34]. However, all proposed narratives are disputed, with political and nationalist biases often influencing the interpretations [10, 11, 35].

Several attempts using genetic data have been made to address the questions of whether the emergence of Slavs was accompanied by a population movement. Studies of genetic variation in modern Europeans [36–38], however, remain inconclusive due to a lack of resolution and the genetic similarities between European populations in general. Power may be gained by haplotype-based methods, which revealed a much higher genetic similarity among Slavic populations than was expected by their geographic distance [39]. This was initially interpreted as the result of a common genetic origin [40], supporting a migration event, but could also be due to recent small effective sizes [41] or both [42].

The few existing studies using ancient DNA samples also reached opposing conclusions. Early works based on matrilineal markers (mtDNA) supported genetic continuity in Central Europe from the Bronze Age to the present [43, 44], while genomic target enrichment data suggested a population turnover in the Volga-Oka interfluve between the Iron Age and Medieval times [45]. Although on a much larger geographic scale, genomic target enrichment data from a temporal cline spanning from Roman times to the Medieval period also revealed a noticeable genetic shift between samples that predate the fifth century and those dating to the eighth century or later in the Balkans. However, the results were not compatible with a complete replacement [46]. Finally, a recent genomic study on a large sample from modern-day Poland also found rather strong genetic differentiation between the Iron Age and Medieval populations, the presence of individuals with similar genomic make-up in both groups led the authors to conclude that there was predominantly genetic continuity between these times [47]. However, the authors admit that “based on these data, one cannot exclude additional migrations from the Eastern Europe, either during the Migration Period or later” [47].

A challenge in using ancient DNA to address the emergence of Slavs is that individuals associated with early Slavic cultures, including Prague-Korchak culture, mostly used cremation as their primary funerary rite, resulting in a sampling gap for the most relevant time period. The earliest Medieval sample in the Polish study [47], for instance, dates to the tenth century, making it difficult to exclude that later processes also contributed to the genetic make-up of those samples. While some Central European samples of the Balkan study date to the eighth century [46], these samples lack archeological context indicative of Prague-Korchak culture.

**Fig. 1:**
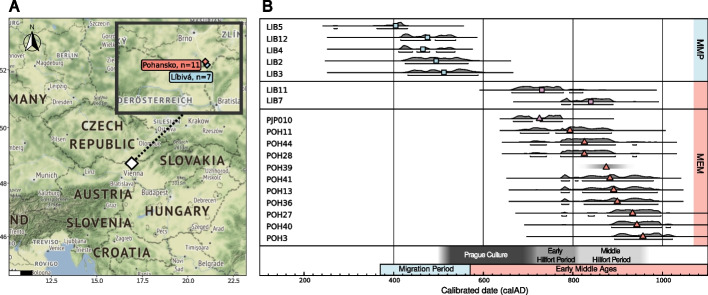
Overview of analyzed samples. **A** Map of Europe showing the location of the analyzed sites. **B** Time axis with ^14^C dates of all samples except POH39, which could only be dated archaeologically. The main chronological periods and also fine-scale archaeological periods are added for comparison (details of.^14^C and archaeological dating are further discussed in the Additional File 1: Section 2.5)

To narrow the time gap in sampling and to expand the genomic view of the likely geographically diverse history of Slavs, we here report high-quality, whole-genome data of individuals from the two burial sites of Líbivá and Pohansko (Fig. 1), located in the core area of presumably the first Slavic principality of Moravia from the ninth century [48–51]. The sites are within 1-h walking distance and played a different part in the socio-cultural context of the Migration Period and the Early Medieval South Moravia (Additional File 1: Section 2).

In multicultural Líbivá, inhumations were archeologically associated with the fifth century CE, while pits, sunken houses, and few settlement graves date between the six/seventh to ninth centuries [52–54]. Importantly, the site was linked to residues of a poor, local and non-transient population during the Migration Period [55], not like many sixth century sites in the Lombard period that are traditionally linked with a short-lasting population movement [56].

In contrast, Pohansko was a fortified settlement of a significant strategic role in the Slavic principality of Moravia and was occupied since the sixth century [57–60], the burial of a newborn child (sample PJP010, H 205) found in one of the earliest Early Medieval features of Pohansko-Severovýchodní Předhradí dating from seventh to eighth century CE [61], to samples from the ninth to tenth century CE, from when there are historical records written in Church Slavic in the region [51, 62].

### Results

Using low-depth whole genome sequencing (median depth 0.0008 X), we screened a total of 54 individuals from the Pohansko and Líbivá sites (Fig. 1), of which 22 had > 20% authenticated endogenous genetic material (Additional file 2: Screening Results). Among those, we chose 17 samples that represent the cross-section of social structure for whole-genome sequencing (see Additional file 2: Screening Results for the archeological descriptions and grave positions that informed the choice). Among the remaining samples with lower preservation, one of particular interest was sample PJP010, the oldest known inhumation associated with the very late stage of the Prague-Korchak culture or with the archeologically continuous Early Hillfort Period which had an endogenous DNA content of only 1.69% (Additional file 2: PJP010 Results). For this sample, we captured 1,233,013 SNPs (“1240 K capture” [63, 64]), resulting in 0.2163X coverage at the 1240 K sites.

We ^14^C-dated all available samples for which genomic data was produced (see Additional File 1: Section 2.5, Figs. S2-5, Tables S2-3). As expected based on the archeological assessment of the graves, most Líbivá samples dated to the Migration Period (fifth century) and all Pohansko samples dated to the Early Middle Ages (seventh to tenth century), except POH39, for which no date could be obtained due to collagen preservation. However, two of the Líbivá samples (LIB7 and LIB11) that did not have any associated grave goods also dated to the Early Medieval period. Notably, LIB11 dated to the seventh to eighth century and is thus among the earliest inhumations chronologically associated with the very late stage of the Prague-Korchak culture or with the archeologically continuous Early Hillfort Period and shows a similar chronology as the PJP010 individual of Pohansko.

For whole-genome sequencing, we used a multi-library strategy to maximize complexity [65] and obtained a median depth of 7.4 X (ranging from 5.1 X to 9.4 X, Additional File 1: Fig. S6). All samples showed post-mortem damage patterns characteristic of ancient DNA (Additional File 1: Fig. S7) and low contamination (< 2%, Additional File 1: Fig. S8). Sample integrity within sequencing runs and within different sequencing events of the same libraries was confirmed via principal component analysis (Additional File 1: Fig. S9). Blank controls were processed alongside the samples during all laboratory steps and their sequencing results evidenced no contamination (Additional file 1: Section 3.1, Additional File 2).

The genetic sex inferred with *BeXY* [66] matched the archeological assignments for all samples but POH39, which was preliminary assigned as female based on poorly preserved and fragmentary skeletal material, whereas genetic sexing suggests an XY karyotype (Additional File 1: Fig. S10). No aneuploid sex karyotypes or autosomal trisomies were found (posterior probabilities < 0.0001 in all cases, highest posterior probability was for individual POH28 karyotype XYY = 0.00001).

#### Genetic shift in the South Moravian Region

**Fig. 2:**
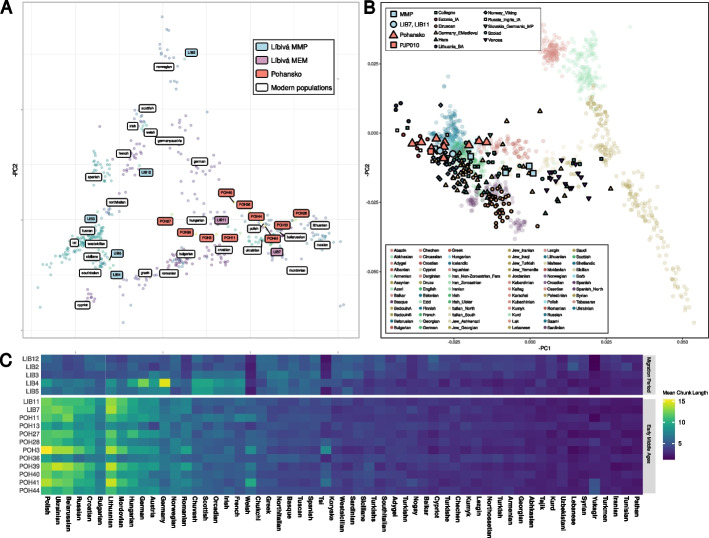
Genomic ancestries of the studied samples. Analysis in panels **A** and **C** has been done on a merged dataset with 500 modern reference individuals from 26 European populations (HellBus dataset, see Methods: Chromosome painting). **A** Principal component analysis (PCA) on the chunk length matrix shown in **C**. **B** Projected PCA. The Migration Period cline overlaps with the Early Medieval position of Pohansko and LIB7 and LIB11. While the MMP samples that are dated to the fifth century lie along a cline typical for Migration Period, spanning the diversity of modern Europe from South to North, MEM samples cluster together more homogeneously on top of modern samples from Eastern and Central Europe. **C** Raw chunk lengths matrix from the present-day painting. The proportion of genome shared with modern individuals shows a strong inclination of MEM samples to modern Eastern and Central European populations, such as the Polish, Ukrainians, Belarusians, or Russians. In contrast, MMP is similar to Northern Europeans in general, with LIB2 being strongly pulled towards Norwegians

The relatively high depth of our whole genome samples allowed us to investigate their genetic composition with the haplotype-based method *ChromoPainterV2* [67] against a large modern reference panel. We found evidence for strong genetic differentiation between the samples dating to the Migration Period and Early Medieval from the studied region. We will refer to these groups as Moravia Migration Period (MMP) and Moravia Early Medieval (MEM), respectively. As is visible both from the chunk length matrix (Fig. 2C) and its visualization as either a dendrogram (Additional File 1: Fig. S11) and a PCA (Fig. 2A), the MMP samples are diverse and spread along a cline of modern populations from the Western Mediterranean (LIB4 and LIB5) to Scandinavia (LIB2). In contrast, the MEM samples form a coherent cluster and are most similar to present-day North-Western Europeans, such as Polish and Lithuanians. Notably, this cluster includes the two samples from Libivá that also date to the Early Medieval period (LIB7 and LIB11) confirming our ^14^C results. To further test for the homogeneity of the MEM samples, we split the MEM samples into an early (PJP010, LIB7, LIB11, POH11, POH44) and late (POH3, POH13, POH27, POH28, POH36, POH39, POH40, POH41) group based on the largest gap in ^14^C dates between POH44 and POH28 (POH39 was assigned based on its archeological dating, see Additional File 1: Section 2.4). Using *ADMIXTOOLS* [68] *qpWave* on a large set of outgroup populations (see Additional File 1: Section 3.2) indicated that these two chronological groups within MEM are not distinguishable (*p* = 0.11757).

A projected PCA based on the 2280 modern individuals genotyped with the HumanOrigins chip [68–70] allowed us to include the infant sample PJP010 (Fig. 2B). On this PCA, LIB7 and LIB11 cluster among the other MEM samples, as does the infant sample PJP010, although the clusters are less clear and partially overlap due to lower power. Yet, several additional analyses confirm the genetic differentiation between the MMP and MEM samples: (1) On a PCA not informed by any modern reference and performed on genotype likelihoods (Additional File 1: Figures S12 and S13), they form distinct (but scattered) clusters. (2) The infant sample PJP010 shares unique gene flow with the rest of MEM samples compared to MMP samples (*f4(MEM,MMP; PJP10, outgroup)* = *0.001157, Z-score* = *3.221*). (3) A supervised *ADMIXTURE* [71] analyses run with 1000 genomes samples [72] inferred a prominent FIN component for the MEM missing in the MMP samples (Additional File 1: Fig. S14). (4) Simple continuity between the MMP and MEM samples could be rejected by testing for cladality with *ADMIXTOOLS qpWave*, as the two groups do not form a clade (*p* < 10^–53^), even when splitting the MEM samples into early and late groups as described above (*p* < 10^–36^; Additional File 1: Section 3.2).

**Fig. 3:**
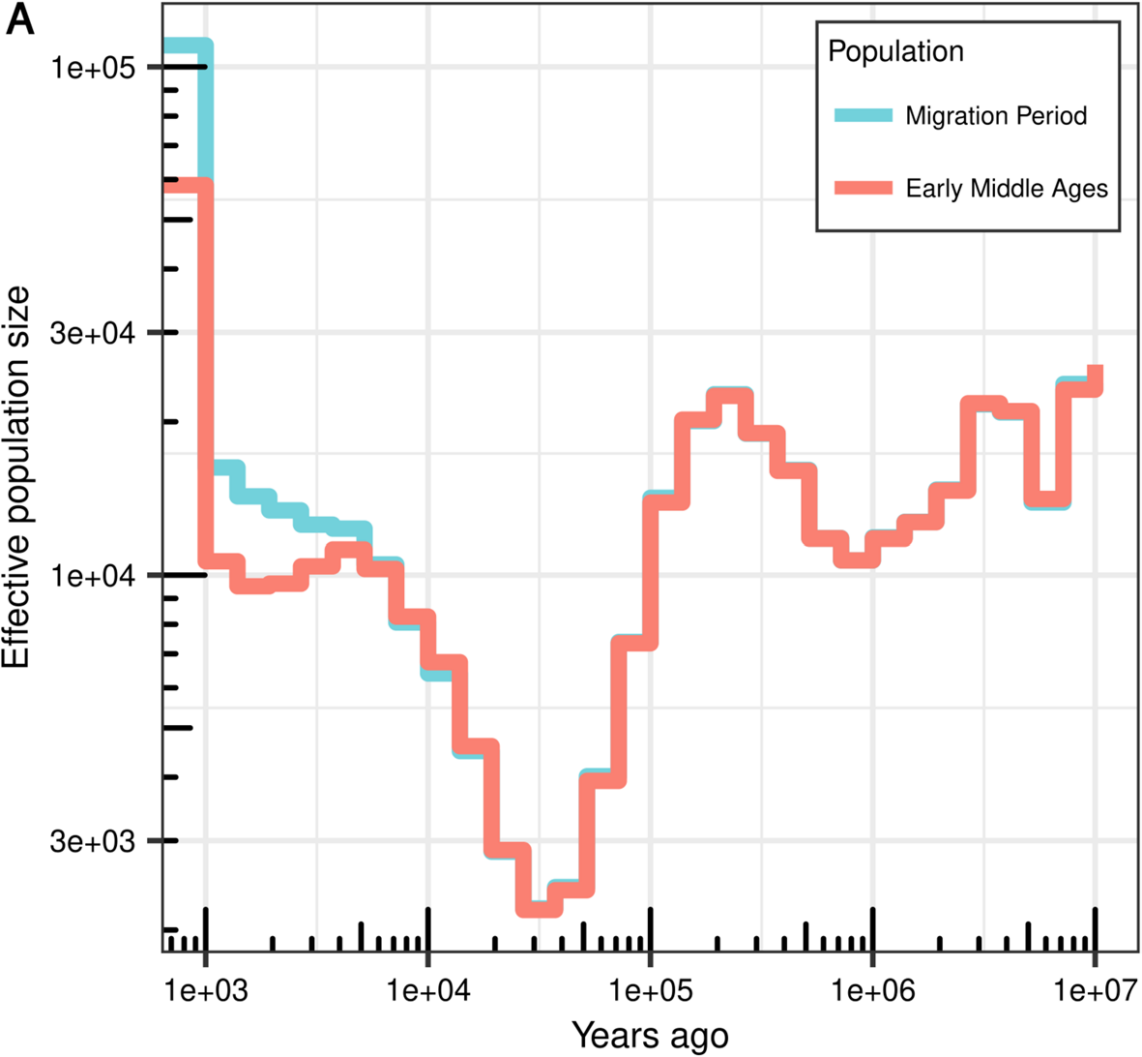
Effective population size estimates of the MMP and the MEM samples through time. Note the divergence of their trajectories at around 6000 years ago

We next inferred genome-wide genealogies with *Relate* v1.1 [73], which simultaneously estimated branch lengths, mutational ages, and past population sizes for all samples. The demographic trajectories inferred for the MMP and MEM samples are very similar for the more distant past, but diverge around 6000 BCE, after which the trajectory of the MMP samples was monotonously increasing while that of the MEM samples is characterized by a population decline (Fig. 3). Although the simple split-model of *Relate* is unlikely reflecting the complex relationship between these populations, the result does support the notion that these populations have distinct histories and rejects a simple model of continuity. Since we did not detect any difference in intra-individual heterozygosity of MMP and MEM samples (*p* = 0.21, Additional File 1: Fig. S15), for instance, the higher population size estimate for the MMP samples may be the result of the recent inclusion of individuals with different ancestries (e.g., LIB2). But we note that the longest runs of homozygosity (ROH) were inferred for two MEM samples (POH11 and POH13) and the only MMP sample with elevated ROH was LIB2 (Additional File 1: Fig. S16).

#### Relationship with other populations

The fifth century MMP samples show exceptional diversity on the PCA and span the entire cline from modern Mediterranean to Scandinavian populations (Fig. 2). A similar distribution of ancestries was previously reported for fifth and sixth century samples from Northern Italy, Hungary, and Slovakia [74–76] and may thus be representative of the genetic make-up of populations associated with Germanic settlements in the larger region at that time. However, *qpWave* does not support a clade of MMP and other fifth and sixth century populations (from each region separately, *p* < 10^−5^, see Additional File 1: Section 3.2), suggesting that the processes that lead to the observed diversity were either recent, varied in space and time or were maintained by social practices [74, 75]. However, the sixth century Lombard-period samples also do not form a clade with the MEM samples and lack the Eastern European component (Additional file 3: Dataset S3.4 and S4).

Since the MMP samples alone are not a good proxy for the ancestry of MEM samples, we used f4 statistics and *qpAdm* modeling to gain further insight into their ancestry. On the PCA (Fig. 2B), the MEM samples cluster with samples from North-Eastern Europe. In agreement, *f4(Outgroup, TEST, MEM, MMP)* tests indicate a genetic drift shared between mostly populations from Northern and Eastern Europe with MEM in respect to MMP (Additional file 3: Dataset S3.2).

Consequently, the only *qpAdm* models for MEM not rejected (i.e., with *p* ≥ 0.05) involve Eastern European precursors (e.g., *Russia_Ingria_IA*, *Poland_Roman*, *Lithuania_Marvele_Roman*, *Poland_Viking*, *Sweden_Viking*, *Russia_Viking*, *Norway_Viking*, *Estonia_IA*, Additional File 1: Section 3.2), thus suggesting a substantial genetic influx into Moravia between the fifth and seventh century. While some of the qpAdm models do also include MMP samples, e.g., 51.8% (MMP + *Lithuania_Marvele_Roman*; the possible model with the highest MMP contribution), we caution that this MMP contribution might easily arise as an artifact due to the scarcity of ancient reference populations from North-Eastern Europe. It indeed appears very plausible that the true source population for MEM had some ancestry shared with MMP that is underrepresented in many of the source populations available, including in *Lithuania_Marvele_Roman*. In line with this interpretation, we found MMP to also be a possible source for Early Medieval samples from further east, such as for instance for the Volga-Oka region [45] (Additional File 1: Section 3.2, Additional file 3: Dataset S6). Consequently, the true contribution of MMP to MEM was likely much smaller than 51.8% and even a potential complete replacement cannot be fully excluded.

Interestingly, using supervised *ADMIXTURE*, we inferred a small component of East-Asian ancestry for the two MEM samples POH3 (1.67%) and POH39 (4.88%) (Additional File 1: Section 3.3) that fall closer to Western European populations in all PCA analyses conducted (Fig. 2, Additional File 1: Figs. S11–S13). While not confirmed with f4-statistics when compared to various Avar-period groups with East-Asian ancestry ([77, 78], Additional File 1: Section 3.3, Figs. S17-20), the preferred 3-way admixture model of the MEM samples inferred by the haplotype-based admixture inference method *MOSAIC* also inferred an Central/East-Asian component of 1.5% that can be dated to 476–732 CE (95% CI 5.7–17.9 generations prior, Additional File 1: Section 3.4). Influx of East Asian Steppe people into Europe during this part of the Migration Period is well described (e.g., [77]), and although the studied sites are located outside the presumed northern periphery of the Avar Khaganate marked by the Thaya river, the border was never absolute as evidenced by historical sources (Fredegar Chronicle translated by [4, 79] and multiple finds of Avar belt fittings north of the border and Slavic culture-typical cremation burial sites south of it [80, 81] (see Additional File 1: Section 2.3). There were even suggestions that Slavs spread under the rule of Avars and were in close contact with them prior to their arrival to the region [11], but that appears unlikely, especially in the cases of the Avar-period elite class [77], since we found East-Asian ancestry in only a few and not in the earliest samples (LIB11, LIB7, PJP010) and we found no uniparentally inherited marker linked to East Asia (Additional File 1: Section 3.5, Fig. S21, Additional File 4).

We finally used the haplotype-based admixture inference method *MOSAIC* to investigate the relationship of our MEM samples to modern Slavic populations. Using modern Belarusians as an example, we found that MEM samples alone are poor proxies and that modern Slavic populations likely derive ancestry from additional, more southern sources (Additional File 1: Section 3.6, Figs. S22 and S23).

### Discussion

Here we report novel ancient genomic samples from modern Moravia, Czech Republic. We found evidence for a major genetic turnover between the fifth and early eighth centuries in that region. This turnover coincides with the arrival of a novel archeological cultural expression, referred to as Prague-Korchak culture in this region, to the late phase of which our seventh to eighth century sample PJP010 can be attributed based on directly associated archeological finds. The data presented here thus supports the notion that this shift of material culture was spread by immigrating people. Since contemporary samples from the source population are not available or have not yet been identified, it remains difficult to quantify the contributions of the immigrating versus local population to Early Medieval samples reported here, as well as the exact sociodemographic processes, including differing marriage patterns (as in [82]) or whether they unfolded rapidly or over several generations. Our estimates, however, suggest that the contribution of the immigrating population was substantial and given the scarcity of data from relevant reference populations, we note that even a scenario involving virtually complete population replacement in this region cannot be ruled out, although this represents one of several possible explanations.

The Prague-Korchak culture is often directly connected to the spread of Slavic speakers [26, 27] and it is thus tempting to interpret our findings as evidence that the Slavic language was brought to Central Europe by immigrating people. It is important to note, however, that the language spoken by the early bearers of the Prague-Korchak culture remains unknown as the earliest written sources connected to the region date to the ninth century, with the exception of an animal bone from the sixth century incised with Germanic runes [31]. Yet our data does support genetic continuity at the studied microregion from our earliest samples (PJP10 and LIB11) that dated to the seventh to eighth century up to our latest samples that dated to the tenth century, a continuity that is also apparent from the material culture [83]. Given this observed continuity from the seventh century onward, it seems plausible to interpret the preceding genetic and cultural shift as the arrival of Slavic speakers in the region.

Whether the appearance of Slavs was accompanied by the migration of people has long been debated [3]. Our results are incompatible with theories of autochthonous development and rather indicate that immigration was substantial, at least for the studied region in South Moravia. A substantial contribution of incoming people to the Early Medieval gene pool is supported by other recent genomic studies [45–47], although the inferred contribution was inconsistent. At a local scale, data from the Volga-Oka interfluve suggested a population turnover in that region between the Iron Age and Medieval times [45]. At a regional scale, Iron Age and Medieval samples from modern-day Poland were interpreted as supporting continuity [47]. And at the large scale of South-Eastern Europe [46], a noticeable genetic shift was reported between samples that predate the fifth century and those dating to the eighth century or later, suggesting a major influx of people but not a complete replacement [46].

Importantly, and in contrast to those presented here, most previously reported samples either lack Slavic-associated archeological context (e.g., Prague-Korchak culture or Early Hillfort Period) or date to later Medieval periods (e.g., tenth century), and their ancestry may have thus been shaped by additional, unrelated events, such as gene flow from neighboring populations. As an alternative explanation for the perceived inconsistency in the estimated influx, we propose a major population shift in most localities, followed by the assimilation of the remaining individuals of the previously local population, mobility from neighboring regions (e.g., from regions associated with Lombards or Franks, [84, 85]), or a combination of both. The early Slavic populations may thus have become much more heterogeneous over the following centuries, in line with the observed variation of ancestries at later periods and the findings of only incomplete replacement in samples of later periods, such as in the Balkans [46]. While the currently available samples do not allow for a careful dissection of the local demographic processes between the fifth and ninth century, in part because early bearers of the Prague-Korchak culture used cremation as their primary funerary rite [25], but also because existing samples are often geographically spread and sparse in numbers, local chronological transects such as the one reported here, provide a view into this dynamic period.

### Conclusions

Novel genomic data of individuals from South Moravia evidences a substantial genetic turnover in the region between the fifth and early eighth centuries. This observed genetic turnover, which appears to coincide with the arrival of early Slavic-culture-associated communities in the region, is inconsistent with models of strict local continuity. Instead, it supports the hypothesis that the observed cultural shift was accompanied by significant population replacement by newcomers of, as our data indicates, North-Eastern European-like genetic ancestry. While the language spoken by these incomers remains unknown, genetic as well as cultural continuity at the regional level links these communities to the Middle Hillfort Period population thought to be the Moravian Slavs mentioned in written sources.

### Methods

#### Radiocarbon dating

Radiocarbon dating of human bones from Líbivá and Pohanko was performed in radiocarbon laboratories in Poznan/POZ (Poznan Radiocarbon Laboratory, Poland), in Laval/ULA (Radiochronology Laboratory, Laval University, Canada) and Vienna/VERA (Vienna Environmental Research Accelerator) on one to two samples from every grave, such that each sample was from a different section of the skeleton. The calibration of the conventional radiocarbon ages and following sequencing were performed using the on-line version of the *OxCal* program, v4.4.4 [86], with the implemented atmospheric calibration curve IntCal20 [87]. Further processing of radiocarbon dates is described in Additional File 1: Section 2.5 using methods published in [86, 88, 89] (Additional File 1: Table S2 and S3, Figs. S2, S3 and S4).

#### Acquisition of ancient DNA sequences

Osteological material has been selected from sites of Pohansko and Líbivá in the Břeclav region that were archeologically extensively studied and contextualized ([57, 90–104], Additional File 1: Section 2"Additional File 2"). Sample preparation, extraction, and library protocols followed the methods described in [65] and references therein, if not stated differently. All laboratory procedures conducted on samples from Pohansko and Líbivá individuals except for PJP010 (see details for the analysis of that sample below) were performed in the dedicated ancient DNA facilities at the IOME, Johannes Gutenberg University, Mainz, which implements a two-lock system for pre-PCR facilities, a chemical and UV-light-based contamination of workspace, materials, and lab clothing, and a strict separation of pre- and post-PCR facilities. All work was carried out in protective trace-lab clothes and we generated blank controls for each step of the protocol.

#### Decontamination and sampling

The bones of all Pohansko and Líbivá samples were photo-documented and UV-irradiated at 254 nm for 45 min per side (two sides) before carefully removing the potentially contaminated outer layer of the bone with a sandblasting machine. For tooth samples, the crown was carefully sawn off as it contains less genetic material and holds odontological information. The bone parts were pulverized as described in [65].

#### DNA extraction

To increase sample complexity, two extractions per sample were performed. DNA extraction and purification followed the protocol described in [65] and references therein.

#### Non-UDG library preparation

Library preparation was performed following [105] with the modifications described in [65] and the following adaptations: The first library of each sample was constructed without UDG treatment to preserve the original damage pattern. For the non-UDG treated libraries, the difference in volume was accounted for with nuclease-free H_2_O. The purification steps with MinElute columns were performed, following the manufacturer’s manuals and eluting in 22 µl of preheated (65 °C) elution buffer. For initial index-PCR, 3 µl of the fill-in product was mixed with 20 µl Accuprime Pfx Supermix and 1 µl of each, the corresponding P7 and P5 index, respectively, and amplified for 6 to 8 cycles. We purified the post-PCR libraries with the MSB® Spin PCRapace kit, following the manufacturer’s manual and eluating in 11 µl of pre-heated elution buffer (65 °C).

#### Low-depth sample screening

Samples from 54 individuals from Pohansko and Líbivá underwent low-depth screening of the first PCR parallels (Additional file 2: Screening Results) on an Illumina MiSeq™ platform at StarSEQ GmbH (Mainz, Germany). Seventeen individuals covering the broadest diversity of the site and reaching the highest endogenous DNA content based on mapping the reads to a human genome reference (GRCh37/hg19) were selected for deeper whole genome sequencing.

#### UDG library preparation

For each selected sample, two to three UDG-treated libraries were generated as described in [65]. The libraries were then amplified in 12 or 18 PCR parallels (for 18 parallels we reduced the sample volume to 2 µl and added 1 µl of nuclease free H_2_O) which were partly merged before purification. Libraries were then either purified individually with the Ampure XT bead system before pooling, or pooled before purification.

#### Whole genome sequencing

Pooled libraries were sequenced on an Illumina NovaSeq S2 Flow cell with either paired-end 2 × 50 or single-end 1 × 100 cycles. The paired-end sequencing was repeated for 12 samples, adjusting the pooled library concentrations based on the first sequencing results.

Bone powder from the femur of PJP010 was obtained in the ArcheoGen sampling laboratory at the Department of Archaeology and Museology, Masaryk University, Brno [106–109], and the subsequent steps leading to data acquisition were conducted in the Ancient DNA Core Unit of the Max Planck Institute for Evolutionary Anthropology, Leipzig. A protocol of [107] was adapted for a femur and utilized to obtain the bone powder. The DNA of the PJP010 individual was extracted and a library was constructed at the Ancient DNA Core Unit of the Max Planck Institute for Evolutionary Anthropology, Leipzig, Germany, following standardized protocols [77]. The library was shotgun sequenced at low depth to explore the preservation status and due to its very low endogenous DNA content (1.69%), this genome was subjected to target enrichment for roughly 1,233,013 SNPs (“1240 K capture”, [64]). The enriched library was sequenced on an Illumina HiSeq400 platform.

#### Raw data processing and local realignment

Unless stated otherwise, programs and their versions were used as described in Additional File 1: Section 3.

The processing of raw data from the PJP010 sample followed the standard pipeline for 1240 K capture data as described in [77].

The newly generated whole-genome samples were processed with *ATLAS* (commit 06d1209, obtained from https://bitbucket.org/wegmannlab/atlas, [110]), using the *ATLAS-Pipeline* (commit 36749d9 unless stated otherwise, obtained from https://bitbucket.org/wegmannlab/atlas-pipeline, [65]) and below, along with a set of reference individuals as outlined in Additional File 2: WG Results [75, 111–115]. Screening samples were only processed with the *Gaia* module.

#### Raw data processing

The following steps were performed using the *Gaia* module of the *ATLAS-Pipeline*: Sequencing quality was checked with *fastqc* 0.11.8 (www.bioinformatics.babraham.ac.uk/projects/fastqc/). Trimming of read-through adapter contamination was performed with *Trim Galore!* 0.6.4 (https://github.com/FelixKrueger/TrimGalore), with no quality filter and a length filter of 30 bp. For paired-end sequencing, the option *–retain_unpaired* was added. A second fastqc analysis was performed within *TrimGalore!* to ensure the successful removal of adapter sequences. Reads were aligned to the human reference genome hs37d5 (ftp://ftp.1000genomes.ebi.ac.uk/vol1/ftp/technical/reference/phase2_reference_assembly.) with bwa 0.7.17 [116] using the *-mem* and *-M* options. Reads with a mapping quality below 30 were filtered out with *SAMtools* 1.9 [117] using option *-q 30*. All further sorting and indexing was performed with *SAMtools*. Read groups were added with *picard-tools* 2.21.1 (http://broadinstitute.github.io/picard/) using the *AddOrReplaceReadGroups* tool. Unmapped reads, orphans, and secondary alignments were removed with *SAMtools* using the *-F 256* option and additionally *-F 4* option for single-end and *-f 3* for paired-end sequenced files. Duplicate reads were marked with *picard-tools* using the *MarkDuplicates* tool and options *REMOVE_DUPLICATES* = *false, AS* = *true*, VALIDATION_STRINGENCY = SILENT and TAGGING_POLICY = All. Libraries from the same samples were merged using *SAMtools* with option *merge*, and duplicates were marked again as described above to tag duplicate reads of the same libraries among sequencing lanes. Read counts, sequencing depth, endogenous DNA-content, and other statistics were obtained with *ATLAS* using *task* = *BAMDiagnostics* and with *SAMtools* using option *flagstat*.

#### Local realignment around indel positions

The following analysis was performed in two steps, using the *Rhea* module of the *ATLAS-Pipeline*: We first identified potential target intervals from 12 modern and 12 ancient samples (i.e., target set specified in Additional file 5) as well as two sets of known indel sites from the 1000 Genomes project (https://storage.googleapis.com/gatk-legacy-bundles/b37/Mills_and_1000G_gold_standard.indels.b37.vcf;https://storage.googleapis.com/gatk-legacy-bundles/b37/1000G_phase1.indels.b37.vcf) with the *RealignerTargetCreator* tool of *GATK* 3.8 [118]. To reduce computational load, we parallelized this analysis per chromosome, re-formatted *GATKs* intervals output format to *picard-tools interval_list* input format (custom bash/awk command), and unified the positions found in each target with the *IntervalListTools* of *picard-tools*, resulting in one target-set per chromosome, containing regions where indels might occur within the population. We then identified individual indel positions for each sample and per chromosome with *GATK RealignerTargetCreator*, transformed the format to *picard-tools* input and unified these private positions with the target-set positions obtained above, resulting in a union of private indel positions and positions from the target list per sample and chromosome. This unified interval list was then transformed back to *GATK* format and the final local realignment step (*GATK IndelRealigner*) was performed providing this target set with option *-targetIntervals*, the known sites with *-known*, as well as a set of downsampled BAM-files (termed guidance set, Additional file 5) of 5 modern and 5 ancient samples of this study, each downsampled to 4X, to increase realignment consistency. The resulting BAM-files of each chromosome were finally merged with *SAMtools* using option *merge*.

#### Genotype likelihoods and genotype calls

The following steps in this section were performed with the *Perses* and *Pallas* modules of the *ATLAS-Pipeline*.

We merged the aligned reads from paired-end sequenced read groups to avoid double-use of bases in the overlapping part. Meanwhile, we split the reads originating from single-end sequenced read groups into two groups based on their read length. The first group consists of reads where the DNA fragment has most likely been sequenced completely, because the reads are shorter than the sequencing length minus 5 bp. The other group consists of reads that represent only part of their respective DNA fragment, because the reads are longer than or equal to the sequencing length minus 5 bp to equal out uneven trimming. This approach enables a more accurate PMD estimation, as PMD occurs towards both ends of a DNA-fragment and hence will differ at the 3’ end for reads that have not been fully sequenced. Both steps, the merging and the splitting, were performed with *ATLAS* using the options *task* = *splitMerge* and *allowForLarger*, and providing read group information with *readGroupSettings*.

Post-mortem damage (PMD) patterns were estimated for each read group with *ATLAS task* = *PMD*, providing the reference with *ref* and enabling the option *filterSoftClips* to remove reads with soft-clipping.

We learned base quality recalibration parameters (short recal-parameters) on 10 million sites highly conserved among mammals as reflected by high RS-Scores, also called GERP scores [119], calculated across the multiple sequence alignments of 88 mammals and provided by Ensembl (http://ftp.ensembl.org/pub/release-96/compara/conservation_scores/88_mammals.gerp_conservation_score/gerp_conservation_scores.homo_sapiens.GRCh38.bw). We used *ATLAS task* = *recal* and provided the calculated PMD parameters with *pmdFile*, restricted the analysis to sites with a minimum depth of two with *minDepth* = *2*, removed reads with soft-clipping using *filterSoftClips* and pooled all read groups that were sequenced on the same sequencing FlowCell using *poolReadGroups*.

We generated two additional output files for downstream analyses:

1. We inferred a VCF with bi-allelic sites by first generating genotype likelihood files (GLF files) with the *ATLAS task* = *GLF* using the options *filterSoftClips*, *trim5* = *2* and *trim3* = *2* and restricting the analysis to the chromosomes 1:24,X,Y using the *chr* option, and second using *ATLAS task* = *majorMinor* with options *method* = *Skotte* and *minSamplesWithData* = *20* and using the GLF files and input.
2. We generated pseudo-haploid calls on known alleles (1240 K sites, [64]) with *ATLAS task* = *call* with options *method* = *majorityBase*,* noTriallelic* and *printAll*, providing the known 1240 k alleles with *alleles* and the reference sequence with *ref*. We used the filters *filterSoftClips*, *trim5* = *2*, and *trim3* = *2* and printed custom format fields with *formatFields* = *GT,AD,AB,AI,DP,GQ,PL*.

#### Contamination estimation

We estimated contamination with two complementary methods (reported in Additional File 1: Fig. S8 and Additional File 2): First, we calculated the number of authentically mapping mitochondrial reads using *ContamMix* 1.0 [63] as described in [65]. Second, for male individuals, we also estimated read count statistics with *ANGSD* 0.917 [120] on haploid X-chromosomal regions (X:5,000,000–154900000) with options *-doCounts 1*,*-iCounts 1*, -minMapQ 30 and -minQ 20 followed by contamination estimation with the contamination script of ANGSD *misc/contamination*, using the HapMap-file HapMapChrx.gz provided with ANGSD.

#### Genetic sex estimation

We inferred the genetic sex of our samples with *BeXY* [66], using the task *sex* with the provided sexing parameters *wgs_hg38_default_atlas_pipeline* for all samples sequenced with whole-genome sequencing and *capture1240k_hg38* for PJP010. We set the parameter –*allowTrisomyForAutosomes 13,18,21* to test for autosomal trisomies. *BeXY* uses mapping statistics, which were obtained with *ATLAS task* = *BAMDiagnostics* (commit c24e82e) and using the option *filterSoftClips*, to infer the posterior probabilities for all sex karyotypes (XY,XX,X0,XXY,XYY,XXX,XXYY).

#### Relatedness assessment

We used *ATLAS task* = *geneticDist* to estimate the euclidean distances between all pairs of Líbivá and Pohansko samples, using the GLFs created during the estimation of genotype likelihoods. We then used a custom *R* script (https://bitbucket.org/wegmannlab/atlas/downloads/Relatedness.R) to apply the method of [121] to transform these distances into estimates of genetic relatedness. No relatedness was detected among all samples. For more details, see Additional File 1: Fig. S24.

#### Imputation

We used *GLIMPSE* 1.1.0 [122] to jointly phase and impute all newly sequenced ancient samples based on the bi-allelic VCF inferred with *ATLAS* (see above). *GLIMPSE_chunk* was first used to split the genome into chunks, after which *GLIMPSE_phase* was used to impute missing genotypes. The imputed chunks were merged using *GLIMPSE_ligate* and haplotypes generated using *GLIMPSE_sample*. All settings were left as default and the 30 × 1000 genomes dataset [72] was used as a reference panel and all samples were imputed at the sites present in that panel. This resulted in a total of 50,342,061 autosomal, phased non-missing genotypes for each ancient sample. This data was used for all haplotype based inferences.

#### Chromosome painting

We used *ChromoPainterV2* [67] to estimate the proportion of genome each newly sequenced ancient sample shared with each individual in a reference dataset of present-day European populations [123], hereafter referred to as Hellenthal & Busby (HellBus). We merged the newly sequenced ancient samples, after being phased and imputed with *GLIMPSE*, with the HellBus dataset, and retained 500 HellBus individuals from 26 European populations, keeping all SNPs common between the two datasets (*n* = 448,637). We then phased all samples again using *shapeit4* [124] and default parameters. We then performed an “all-v-all” painting with *ChromoPainterV2* in which each haplotype is compared to each other haplotype in turn, resulting in coancestry matrices which give the counts and the total length of segments each haplotype shares with each other haplotype. Truncated principle components of the resulting chunk lengths matrix were calculated using the *IRLBA* R library [125].

#### fineSTRUCTURE

We then used *fineSTRUCTURE* 0.0.5 on the chunkcounts matrix to identify genetically homogenous clusters of samples. The first stage used 10^6^ burn-in iterations and 2 × 10^6^ sample iterations with a thin interval of 10^4^. We then ran the tree building stage for 10^5^ sampling iterations. Using the *fineSTRUCTURE* library, we performed a PCA and a population dendrogram, the latter using the tree building algorithm.

#### Relate

We used *Relate* v1.1 [73] to infer population divergence times and effective population size histories following default recommendations. Estimated population sizes and cross-population coalescence rates were scaled using a mutation rate of 1.25 × 10^−8^ mutations per site per generation and a generational interval of 28 years.

#### Mosaic

We used *MOSAIC* 1.3.7 [126] to infer admixture events and dates in ancient samples. We used the full set of 610 individuals from 48 present-day European and Asian populations from the HellBus dataset as surrogates for the admixing sources. Ancient populations were modeled as either a 2 or 3 way mixture of these present-day populations and the non-target ancient population. Upper and lower quantiles for admixture dates were estimated from a bootstrap procedure implemented using the *bootstrap_chromosomes_coanc_curves* function from the *MOSAIC* API with default parameters.

#### Genetic diversity estimation

We used the method of [127] implemented as the task *theta* in ATLAS to infer genome-wide heterozygosity (*θ*) with option *thetaGenomeWide* on 17,737 neutral 1-kb autosomal loci ([128]; as in [129]) from BAM-files. We ran 100 bootstrap estimates for *θ* for all samples, provided the learned PMD and recalibration parameters (using options *pmdFile* and *recal*), removed soft-clipped reads with *filterSoftClips*, and filtered out all sites with a sequencing depth below 2 with parameter *minDepth*.

#### PCA

We used *PCAngsd* [130] with default parameters to infer a PCA based on genotype likelihoods. As input, we used the bi-allelic VCF file inferred with ATLAS (see above), which we filtered for a minimum minor allele frequency of 0.05. We did that by first inferring low frequency positions with *ATLAS task* = *alleleFreq minMAF* = *0.05* and then identified positions with *vcfTools*. The VCF file was transformed to Beagle file format using *ATLAS task* = *VCFToBeagle*.

#### Projected PCA

We obtained a projected PCA with *smartpca* from the *EIGENSOFT* v601 package [131]. To do so, we merged the pseudo-haploid calls obtained at 1240 K sites with *ATLAS* with an ancient and modern reference dataset. The ancient individuals’ genotypes were obtained by merging with the AADR dataset [132] v62.0 and the modern ones from a reference genome-wide panel of 2280 modern individuals genotyped with the microarray technology using the commercial HumanOrigins chip [68–70]. Modern individuals contained a set of Western Eurasian present-day populations as reported in [70] and further adapted in [77]. We then used the modern reference samples and inferred a PCA with *smartpca* from the *EIGENSOFT* v6.0.1 package [131]. To project the genotypes of the ancient individuals on top of the modern populations, we used the *lsqproject* and the *autoshrink* options.

#### F-statistics

We used the *f3*, *f4* [133, 134], *qpWave*, and *qpAdm* functions of the *admixr R* package [135, 136] utilizing *ADMIXTOOLS* v702 [68]. These analyses were restricted to the 1240 k sites, and for the whole-genome samples we used the pseudo-haploid calls obtained for those sites with *ATLAS*. The reference ancient populations were obtained by merging the pseudo-haploid calls obtained with *ATLAS* for the newly sequenced individuals with the AADR dataset [132] v62.0.

#### Supervised admixture analysis

We combined the pseudo-haploid calls obtained with *ATLAS* for the newly sequenced individuals with calls of the 1000 genomes project at the same sites using a custom script (see also [65]) and plink (v1.9, [137]) and converted the data set into *EIGENSTRAT* format. We excluded 57 SNPs with differing alleles between the 1000 genomes and the 1240 K data sets. We then performed supervised admixture analysis with *ADMIXTURE* v.1.3.0 [71] and performed cross validation (-cv) for multiple seeds (-s). See Additional File 1: Section 3.3 for details regarding the grouping of populations for plotting.

#### ROH analysis

We combined the pseudo-haploid calls obtained with *ATLAS* with the 1240 K reference panel using a custom script and plink (v1.9, [137]) and converted the data set into *EIGENSTRAT* format (*convertf -p*, https://reich.hms.harvard.edu/software/InputFileFormats). We then used *hapROH* (v0.3a4, [138]) with default parameters to identify runs of homozygosity (ROHs) on our samples.

#### Y-chromosome and mitochondrial haplogroups

Haplogroups were inferred with the *Y-Lineage-Tracker* [139] and *HaploGrep* v2.51 on consensus mitochondrial genomes produced by Schmutzi [140, 141]. The estimated haplogroups were compared with published haplogroups both in the past and nowadays [40, 46, 112, 114, 142–144] (Additional File 1: Section 3.5).

## Supplementary Information

Additional File 1. Supplementary Materials for Ancient genomes provide evidence of demographic shift to Slavic-associated groups in Moravia.

Additional File 2. Dataset S1.

Additional File 3. Dataset S2, Dataset S3, Dataset S4, Dataset S5, Dataset S6.

Additional File 4. Dataset S7.

Additional File 5. Dataset S8.

Additional File 6. Peer review history.

## Data Availability

The newly generated datasets supporting the conclusions of this article are available in the European Nucleotide Archive repository [145]. Datasets used as reference for genetic analyses are available in the European Nucleotide Archive repository or in the National Center for Biotechnology Information [146–151]. The source code for ATLAS [110] and the ATLAS-pipeline [65] are available at https://bitbucket.org/wegmannlab/atlas and https://bitbucket.org/wegmannlab/atlas-pipeline, respectively. The version used here was also deposited at Zenodo [152] under a GNU General Public License v3.0.
