# Supplementary material for: Ancient genomes provide evidence of demographic shift to Slavic-associated groups in Moravia
Source: Genome Biol. 2025 Sep 3;26:259. doi: 10.1186/s13059-025-03700-9 (PMC12409924; doi:10.1186/s13059-025-03700-9)
Supplement: Supplementary file 1 — Additional File 1. Supplementary Materials for Ancient genomes provide evidence of demographic shift to Slavic-associated groups in Moravia. [file 13059_2025_3700_MOESM1_ESM.pdf]

Supplementary Materials for

Ancient genomes provide evidence of demographic shift to Slavic-associated groups in Moravia

Ilektra Schulz[+,1,2], Denisa Zlámalová[+,3], Carlos Reyna[1,2], Sam Morris[4], Guido Alberto Gnecchi-Ruscone[3,5], Raphael Eckel[1,2], Renáta Přichystalová[3], Pavlína Ingrová[3], Petr Dresler[3], Luca Traverso[5], Garrett Hellenthal[6], Jiří Macháček[#,3], Daniel Wegmann[#,*,1,2], Zuzana Hofmanová[#,*,3,5]

[1]Department of Biology, University of Fribourg, 1700 Fribourg, Switzerland
[2]Swiss Institute of Bioinformatics, 1700 Fribourg, Switzerland
[3]Department of Archaeology and Museology, Masaryk University, Brno, Czech Republic
[4]Department of Genetics, Evolution and Environment, University College London Genetics Institute (UGI), University College London
[5]Department of Archaeogenetics, Max Planck Institute for Evolutionary Anthropology, Leipzig, Germany
[6]UCL Genetics Institute, Dept of Genetics, Evolution and Environment, University College London, United Kingdom

* Daniel Wegmann, Zuzana Hofmanová

[#,+] These authors have contributed equally.

Email: hofmanova@mail.muni.cz; daniel.wegmann@unifr.ch

This PDF file includes:

Supporting text

Supplementary figures S1 to S24

Supplementary tables S1 to S3

Legends for Dataset S1 to S8

SI References

Other supporting materials for this manuscript include the following:

Dataset S1 to S8

1 Theories about the origins of Slavic groups in Central Europe **2**
2 Archaeological background in Moravia **4**
2.1 The sites of Pohansko 5
2.2 Břeclav - Líbivá 7
2.3 Avar influence in the region of Břeclav 8
2.4 Description of the selected graves 8
2.5 Dating 19
3. Genetic analysis **27**
3.1 aDNA authentication 28
3.2 qpWave and qpAdm 33
3.3 Admixture analysis 35
3.4 f-statistics 38
3.5 Uniparentally inherited markers analysis 44
3.6 MOSAIC 46
4 Data availability **50**
5 Legends for Dataset S1 to S8 **50**
6 SI References **51**

1 Theories about the origins of Slavic groups in Central Europe

"Slavs" is an ethnonym used in written sources for the population that appeared in South-Eastern Europe in the 6th century and a little later also in some other parts of Europe. The rise of the Slavs represented an indisputable cultural and political shift in Europe, where the Slavic language zone was (and still is) the third major one (1). On the basis of the glottochronological test, linguists assume that Slavs lived before the 5th century north of the Carpathian Mountains and around the source of the Vistula River. As a result of migration, they spread over a wider area and split into the western Sclaveni and eastern Antes/Antae (2). According to other scholars, the Slavic language should be the result of the linguistic convergence of a wide range of related dialects (or languages), which merged into a whole language system called koine (3).

Although both linguistic theories about Slavic language are conceptually incompatible, no one doubts that, in the 7th century, somewhere between the Avar Khaganate in the Carpathian Basin, Merovingian Frankish Empire to the West (3), and the Balkan Peninsula to the South, people labeled as Slavs existed in written sources. However, a hot topic of academic debate is the nature of the process that led to the formation of this particular ethnolinguistic group. Some authors even believe that the supposed Slavic ethnicity is a mere "social construction" (3). They admit the possibility that in Central Europe earlier groups, indigenous as well as migrant, e.g. the Germanic-speaking Lombards (4), effectively "became" Slavs by virtue of a series of dramatic transformations in both economy and society, which matched an accompanying linguistic change (3). They are leaving aside the fact that, in the year 568, the Lombards, or at least a part of them, left the region and settled in the north of Italy for fear of the Avars, as written sources stated (5, 6) and archaeogenetics confirmed (7). Arguments in favour of the continuity of the local

population have been based so far on linguistic analysis of toponyms, mainly hydronyms (8, 9), on specific and rare artifacts like combs (3), or possible analyses of complete mtDNA (10, 11). Most archaeologists and historians, on the contrary, advocate the migration of Slavic-speaking people from the supposed homeland, which is usually located outside of Central Europe, usually in what is now Ukraine and Belarus (12).

The question of whether Slavs or Slavic-speaking people did physically migrate from East to West has remained hotly contested, with the debate substantially influenced by political and nationalist biases (13, 14) or, more recently, post-truth populism (15).

The archaeological expression of the Slavic social model might be the so-called “Prague culture” with Prague-type pottery. At the end of the 7th century, we can identify a characteristic set of this particular material cultural form across vast territories of Europe (16–18). Dislocation of the settlements of the Prague culture correlates with the area where a Slavic language was used in historically documented periods (19), e.g. in Great Moravia in the territory of today's Czech Republic and Slovakia in the 9th century CE.

Characteristic features of Slavic-associated settlements as defined by M. Parczewski (18) include their location along river valleys with houses typically being sunken-floored huts of a certain size and building material. The inhabitants of such settlements were self-sufficient and lived with poor standards, not having developed any handicrafts yet which is also mirrored in the undecorated pottery of so called Prague type. Cremation was their main funeral rite. These settlements have been found in South Moravia, as well as lower Austria, dating to Early Middle Ages (20).

However, the huts, pots and other archaeological finds cannot work as the only or main indicator of Slavic ethnicity (13, 21). Moreover, we have no idea what languages the bearers of Prague culture spoke. Nothing of their language has survived (14) because these people were illiterate and left no texts or inscriptions except for the runic bone from Lány (22).

There are several archaeological models which could explain the cultural and linguistic slavicisation of Central Europe. The main two competing scientific concepts – autochthonic and allochthonic – were re-formulated almost simultaneously in the 1970s (23).

According to the allochthonous (from a Central European perspective) concept, the homeland of the Slavs was the area of the upper Dnieper Ukraine, where the Proto-Slavic culture (also called the Kyiv type of post-Zarubinets culture complex) was formed in the forest zone during the 4th century CE. Later, in the 5th century, the bearers of this culture expanded southwards, to the forest-steppe zone, and westwards. Their habitus was manifested by a sedentary lifestyle and preference for agricultural activities (in contrast to the Avars and other nomadic groups) and materialized in the Prague and Penkovka archaeological

cultures. The great migration of the Slavs is believed to have begun in the 2nd half of the 5th century CE and culminated in the 7th century when the people living in the Slavic style reached the Elbe River in the West, the Baltic coast in the North and Peloponnese in the South.

According to the autochthonic (from a Central European perspective) point of view, the ethnogenesis of the Slavs began in the territory between the Odra and Dnieper rivers in present-day Poland and western Ukraine. The origin of the Slavs stemmed from the biological, cultural and linguistic substrate formed by the local prehistoric populations. It was closely linked to waves of migration of Indo-European peoples, who already spoke specific languages in the Bronze Age, between 1300 and 900 BCE. The direct predecessor of the early Slavic archaeological cultures in the western part of the presumed homeland should be the Przeworsk culture (23).

The third and fundamentally different model of Slavicisation is provided by the collective action theory, which is focused on the multiple strategies from the bottom-up perspective. A human subject is regarded as a rational social actor (24). The Slavic habitus could potentially offer more personal freedom and economic independence than the previous one and led to the successful process of assimilation of various indigenous and migrant groups (25). According to Sebastian Brather, the new Slavic ‘model’ was successful because, beyond the old polarity of ‘Romans’ versus ‘barbarians’ in the Late Antique, it offered chances for further economic and social developments (26). Some authors consider the early Slavs the most successful “hippies” Europe has ever known. However, this picture is not fully convincing because Slavic groups successfully established their political and military dominance. Already, in the 6th century, they were known for military effectiveness, through which, in the end, they imposed a new social order across Central and Eastern Europe. Nevertheless, Slavicisation certainly had its more voluntary component, at least in its early stages, since some Slavic groups were open to indigenous populations willing to adopt the new cultural forms (27). This new group of kinfolk (not related by blood) possess the right to joint control of the land. In this way, a new personal and collective identity was hereby established.

The theories described above are mutually exclusive. Unification of views on the origins of the Slavs based on archaeological or linguistic evidence alone is impossible at present (23). Contextualisation and an interdisciplinary approach are necessary to explain the origin of Slavic Europe. One of the new data sources that could bring fresh perspectives into the debate is genetic analysis.

2 Archaeological background in Moravia

Both newly studied burial sites Pohansko and Líbivá are located near the South Moravian city Břeclav (Czech Republic) (Fig. S1). The nearby swampy landscape is partly wooded and partly grassy, geologically mostly gravel, sand and clay sediments deposited by the close Thaya river (28). In the Early Middle Ages they were part of a larger settlement complex including burial sites, which formed one of the main centres of Great Moravia, one of the first Slavic polites ever (9th century CE). Presence of Early

Medieval ceramics in the territory of Břeclav district indicates the existence of other not yet discovered settlements (29–31). According to Macháček (32), Pohansko, as the main central agglomeration, could not provide for its own agricultural production and had to rely on supplies from neighboring settlements such as Líbivá.

In Late Antiquity, this area was located in the foreland of the Limes Romanus. It was part of the Barbaricum, inhabited by tribes of Germanic origin, such as the Suebi and, later, the Longobards. According to written sources, the Germanic gentes, or at least some of them, left East-Central Europe. They were replaced by a Slavic-speaking population (1).

2.1 The sites of Pohansko

The Early Medieval fortified settlement Pohansko is located 2 kilometers south of the city of Břeclav in South Moravia. The settlement lies 155–157 meters above sea level (28). More than half century of systematic research revealed signs of settlement lasting from the arrival of Slavic populations into the territory in the 6th century until the 10th century (33, 28, 34–39). The premises lined with a rampart still visible to this day consisted of multiple housing estates, sacral buildings and burial sites. However, settlements have also been found outside the fortification. The map in Fig. S1 shows the arrangement of the whole Pohansko site.

The development of Pohansko began in the 6th century CE, when the presumed Slavic invaders appeared in Central Europe. Apart from the settlement, the early Slavic period is represented in Pohansko by a cremation cemetery (55 graves). A specific case is the burial of newborn child H 205 from Northeastern Suburb, which was found in the fill of the storage pit No. 174 together with the ceramics from the 7th/8th century CE and can be assigned to the late phase of Prague culture (39). It is currently the oldest known human skeleton directly related to the material culture associated with the Slavs.

Pohansko became an important central place somewhat later, in the 9th century CE, when it was fortified and rebuilt according to a unified urban planning concept on the site of an earlier agricultural settlement. Its purpose was military protection and the control and management of long-distance trade. At the same time, professional craft production was concentrated here. The ruler's residence - the Magnate's Manor - was also located in Pohansko (32).

The graves analyzed in this study (see Supplementary Table 1) are not cremations, but later inhumations (8th to 10th century CE), which were selected in order to include multiple burial units, as diverse as possible, with a low probability of biological relatedness between the studied individuals. Here we provide a description of all the units included in this study.

From the burial ground around the first church located in the Magnate's Manor, one individual (POH44) from a grave with a sword was selected for analysis. The cemetery is located in the north-western part of the settlement inside the fortification (36). The beginning of burials is dated to the 9th century right after finishing the building of the church and the cemetery ceased its function in the 10th century after the abandonment of the manor (28). It is considered to serve as a Magnate's graveyard with 414 individuals buried there with very rich grave goods (36). Among them, 4 graves contained swords, 8 axes, 32 spurs and 46 gold and silver jewellery (32, 40).

The Lesní školka (Forest Nursery) burial ground contains 80 graves dating to the 9th century (32, 34). Graves were scattered among standard settlement features, some isolated, others in groups. Most graves had few or no grave goods. In sharp contrast to the cemetery around the church in the Magnate Court, there were more skeletons of women than of men. This part of the settlement is considered to be a craft area and its inhabitants presumably were only ordinary members of the Pohansko community (32, 34, 41). We analyzed five graves from the described burial unit.

Excavation in the so-called Lesní hrúd (Forest Dune) unearthed 105 settlement features, 34 inhumation graves and one horse burial. The spectrum of finds roughly matches the findings from the Lesní školka with the exception of three graves with silver earrings. The remaining graves, mostly with few grave goods, fall within the standard known from the other excavated locations inside the stronghold. One skeleton was analyzed (POH3) (32).

The settlement in the Southern Suburb, where extensive salvage excavations were carried out between 1975 and 1979. The exploration of this huge section of the site covering an area of 8.3 has revealed a fairly dense occupation. A total of 480 settlement features and around 205 inhumation graves were unearthed. As typical finds from the Southern Suburb include weapons and horse gear, it is more likely that at least some of those who lived there were members of a military retinue, living in separate quarters together with their families, and possibly servants. That the Southern Suburb was indeed inhabited by warriors results from the analysis of finds from associated graves, which, unlike those from the craftsmen‘s quarter, have produced a significant quantity of weapons (sword, spearheads, axes, arrow tips) and horse gear (spurs). Some of those graves appear to have been clustered in two small burial grounds, with others scattered among the settlement structures, either isolated or in small groups (35, 38).

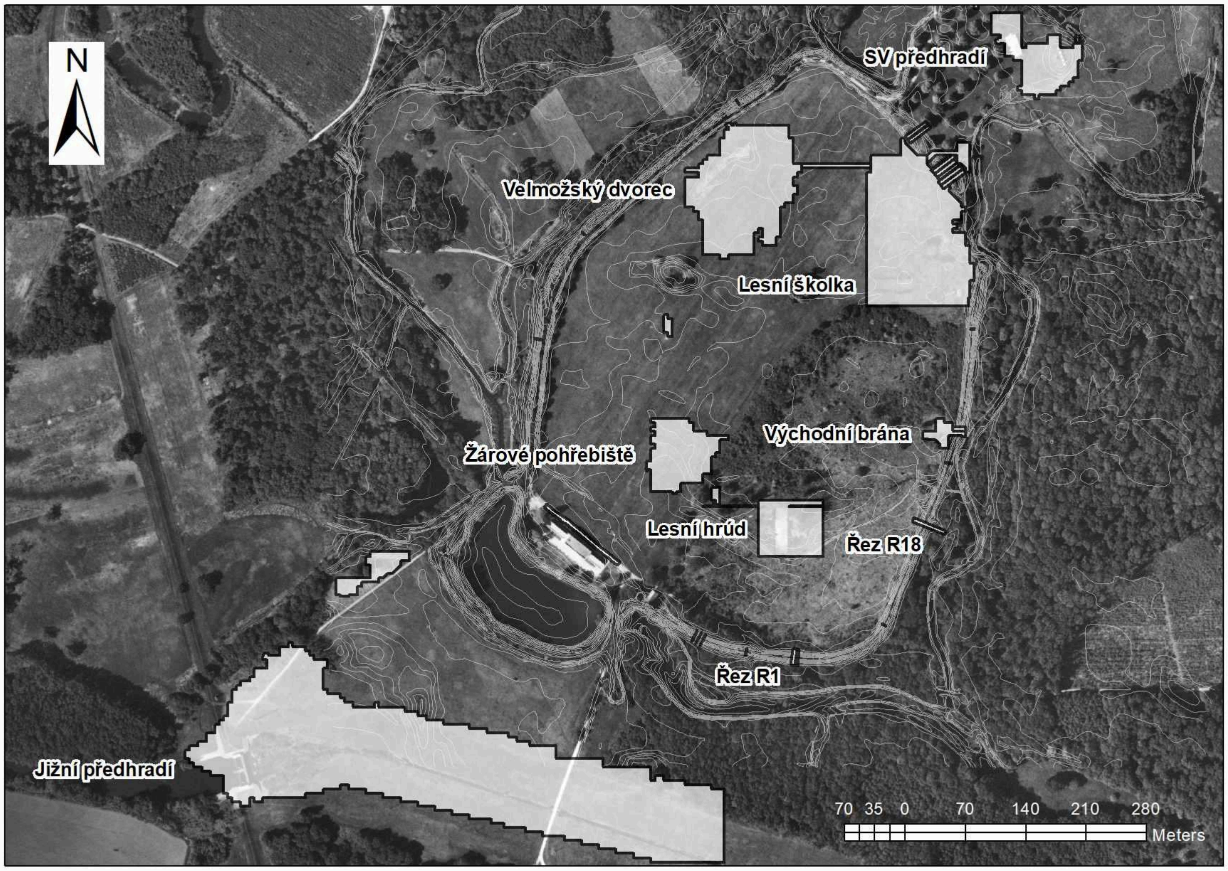

Fig. S1: Map of the arrangement at Pohansko burial sites. The largest unit within the palisade lining was the Magnate's Manor (Velmožský dvorec) containing a church and a large burial ground located around. There was another burial site south of the Manor containing cremated remains (Žárové pohřebiště). The other settlements were located both inside the palisade lining: Lesní hrúd and Lesní školka, and outside: Jižní předhradí and Severovýchodní předhradí. The second church and its associated burial ground was found within the premises of the SV předhradí (32).

2.2 Břeclav - Líbivá

Břeclav - Líbivá is one of the settlements in the agricultural hinterland of Pohansko. It is located approximately 5.5 kilometers northwest of Pohansko. The site is considered to be multicultural since traces of settlements dating from prehistory to the Great Moravian period were discovered within the 1,285 m2 studied ground (42). The burial site consists of 15 graves and two housing estates with 16 human remains. Only 6 graves were furnished with culture-distinguishable artifacts that helped date the burial site into the 5th century in the Migration Period (43). According to Tejral (44), individuals from Líbivá probably belong to the indigenous Sueibi population that got under pressure from the expanding Eastern Germanic people from the Carpathian Basin, who were allies of Attila's Huns. Settlement existed here from the 5th century until the Great Moravian period, which is evidenced by the superposition of excavated graves and settlement features from various periods (42). However, it is assumed that there was a gap of approximately a century between the Migration Period and the Early Medieval phase of settlement.

2.3 Avar influence in the region of Břeclav

Our area of interest on the lower bank of the river Dyje/Thaya borders the northern periphery of the Avar Khaganate. Even at the beginning of the early Middle Ages - in the second half of the 6th and 7th centuries - it is hardly possible to speak of political or cultural borders in this region. The entire area between South Moravia and the Danube Valley in Lower Austria was sparsely but continuously populated by a population whose material culture manifests itself in Prague-type pottery, traditionally associated with early Slavs.

However, as early as the 8th century, the first diversification occurred here. Two spheres of influence developed here: a "Slavic" and an "Avar" one. South of the Thaya/Dyje river, inhumations with typical Avar belt fittings made of bronze are found, e.g., Mistelbach and Dolní Dunajovice. On the other hand, cremation cemeteries of Slavic character are found to the north, e.g. Pohansko, Lanžhot/Trnava Forest or Přítluky. The Avar Khaganate was thus apparently demarcated in the north by the Thaya/Dyje River, whereby the marshes along the river's course could be considered a secure and natural border. However, the fact that the border was blurred and permeable is evident from several cremation burials dating to the 7th and 8th centuries on the southern bank of the Dyje. On the other hand, solitary but numerous finds of Avar belt fittings come from the Slavic area, as they are known from strongholds and other sites not only in Moravia but also in Bohemia, Slovakia and southern Poland (45, 46).

2.4 Description of the selected graves

The overview of grave numbers and laboratory IDs can be found in Supplementary Table 1.

Locality Břeclav – Pohansko, site Lesní hrúd (Forest Dune):

Grave 6

Excavation year: 2000

Square: C07-40

Burial pit: irregularly rectangular in plan; tub-shaped cross-section; tub-shaped longitudinal section; smooth straight walls; irregularly convex bottom; dimensions: 207 x 61 x 29 cm.

Burial pit design / container: burial container was not archaeologically attested; the position of collarbone and shoulder blade suggests that the body rested in a narrow hollow space.

Buried individual – sex, orientation and position: female; orientation towards W–E; position of skeletal remains: supine; the skull resting on the left temple; the right upper extremity is stretched, distal parts of the forearm bones are laid across the collum femoris; left upper extremity is stretched along the body; right lower extremity is stretched, slightly turned to the left; left lower extremity in stretched position; dislocated foot bones.

Grave goods: silver earring of so-called Veligrad type; iron object, probably a knife, and below it a fragment of a crystal pebble.

Anthropological characteristics: skeletal remains of an adult female (morphoscopic traits of the pelvis, postpartum alterations), about 45–55 years old (according to dental abrasion), stature 166.5±4.49 cm.

Literature: (36).

Locality Břeclav – Pohansko, site Lesní školka (Forest Nursery):

Grave 23

Excavation year: 1978

Square: B71-62

Burial pit: trapezoidal in plan, narrowed towards the head; sections – not examined; bottom – not examined; dimensions: 205 x 90 x 30 cm.

Buried individual – sex, orientation and position: female; orientation towards SW–NE; position of skeletal remains: supine; the skull resting on the occipital region, slightly turned to the left, mandible is dislocated on cervical vertebra; upper extremities are stretched along the body, the forearm bones of the left upper extremity are dislocated to the area between the foreleg bones of both lower extremities; lower extremities in stretched position.

Grave goods: none.

Anthropological characteristics: skeletal remains of an adult female (postpartum alterations); 40–50 years old (according to dental abrasion); stature 172.1 cm (calculated according to femur dx.).

Literature: (34, 36).

Grave 25

Excavation year: 1979

Square: B79-69, B79-70

Stratigraphic relation: eastern corner of the burial pit of Grave 25 intruded the western corner of the pit of Grave 32.

Burial pit: rectangular in plan; tub-shaped cross-section; tub-shaped longitudinal section; perpendicular walls; inclined even bottom; dimensions: 185 x 55 x 15 cm.

Buried individual – sex, orientation and position: male; orientation towards NNW – SSE; position of skeletal remains: supine; the skull resting on the occipital region, opened jaws; upper and lower extremities in stretched position.

Grave goods: large iron tongue-shaped strap-end; prong of a belt buckle; iron knife; three-pointed antler case; two iron strap-ends; animal bone – probably a remnant of food offerings.

Anthropological characteristics: skeletal remains of an adult male (morphoscopic traits of the pelvis); 35–40 years old (according to dental abrasion); stature 158.6 cm (calculated according to femur dx.).

Literature: (34, 36).

Grave 29

Excavation year: 1979

Square: B77-71

Stratigraphic relation: north-western part of the burial pit was embedded in the fill of settlement feature No. 123; the grave was intruded by a power cable trench.

Burial pit: rectangular in plan; tub-shaped cross-section; tub-shaped longitudinal section; perpendicular walls; flat even bottom; dimensions: 230 x 70 x 50 cm.

Buried individual – sex, orientation and position: male; orientation towards NW–SE; position of skeletal remains: supine; the skull is dislocated above the left shoulder blade; the extremities originally were stretched; dislocation of the rib cage, the left upper extremity and the foreleg bones of both lower extremities.

Grave goods: iron knife; pointed bone object, so-called weaving sword; two iron fragments.

Anthropological characteristics: skeletal remains of an adult male (morphoscopic alterations on the pelvis, metrical data of the talus); 35–40 years old (according to dental abrasion); stature 177.8 cm (calculated according to femur dx.).

Literature: (34, 36).

Grave 32

Excavation year: 1979/80

Square: B79-70, B79-69

Stratigraphic relation: eastern corner of the burial pit of Grave 25 intruded the western corner of the pit of Grave 32.

Backfill: in no way different from the subsoil, the only dark spot is visible in the foot region.

Buried individual – sex, orientation and position: female; orientation towards SW–NE; position of skeletal remains: supine; the skull is slightly turned to the left; upper and lower extremities in stretched position.

Grave goods: none.

Anthropological characteristics: skeletal remains of an adult female (postpartum alterations); 20–24 years old (according to dental abrasion); stature 146.9 cm (calculated according to femur dx.).

Literature: (34, 36).

Locality Břeclav – Pohansko, site Velmožský dvorec (Magnate's Manor), cemetery at the first church:

<u>Grave 174</u>

Excavation year: 1959

Square: A13-58

Stratigraphic relation: Grave H 174 intruded a trench of an older church enclosure; the grave itself was intruded by later graves H 101, 123 and 124.

Burial pit: the ground plan is rectangular with rounded corners; sections – not examined; bottom – not examined; dimensions: 280 x 125 x 140 cm.

Burial pit design / container: traces of a mouldered wooden case with presumed dimensions 200 x 53 cm.

Buried individual – sex, orientation and position: male; orientation towards SW–NE; position of skeletal remains: supine; the skull is fragmented; upper and lower extremities in stretched position.

Grave goods: Type X iron sword; sword suspension fitting; iron knife; two razors, fire steel, two strike-a-light stones, needle, key, two iron knives, bone handle of an iron object; axe; a pair of spurs with remnants of spur straps (buckle, loop); fragment of iron sheet.

Anthropological characteristics: skeletal remains of an adult male (according to metrical data of the talus); 40–50 years old (according to dental abrasion); stature undetermined; fracture of nasal bones (ossa nasalia).

Literature: (40, 36).

Locality Břeclav – Pohansko, site Jižní předhradí (Southern Suburb):

<u>H 38</u>

Excavation year: 1975

Square: L35-98, L36-98

Burial pit: rectangular in plan; tub-shaped cross-section; longitudinal section – not examined; flat bottom; dimensions: 253 x 126 x 66 cm.

Buried individual – sex, orientation and position: male; orientation towards NW–SE; position of skeletal remains: supine; the skull was crushed, probably in the occipital region; upper extremities are stretched along the body; lower extremities are stretched; thoracic bones are decomposed, the preserved ribs are dislocated.

Grave goods: iron axe; iron razor or folding knife; four amorphous iron fragments; a pair of iron spurs.

Anthropological characteristics: skeletal remains of an adult male (according to fragments of a femur); 35–40 years old (according to dental abrasion); stature 159.7 cm (according to femur sin.); dental enamel hypoplasia.

Literature: (36, 47).

<u>H 42</u>

Excavation year: 1976

Square: L39-98

Burial pit: rectangular in plan; cross-section with two steps, probably artificially made in search for the original shape of the burial pit; longitudinal section with two steps, probably artificially made; flat bottom; dimensions: 238 x 105 at a depth of 36 cm from the cleaned subsoil; 200 x 58 at a depth of 46 cm from the subsoil.

Burial pit design / container: burial pit was stepped (it is not entirely clear whether or not this adjustment is original), in approximately one half of the northern wall there was a posthole of 37 cm in diameter, which already showed up on the cleaned subsoil; according to photographic documentation it reached the depth of the first step, that is 36 cm from the subsoil; on the basis of preserved documentation it cannot be clearly decided whether the posthole is connected with construction of the burial pit.

Buried individual – sex, orientation and position: male; orientation towards W–E; position of skeletal remains: supine; the skull resting on its base, mandible is situated in the place of presumed cervical vertebrae; upper and lower extremities are stretched.

Grave goods: iron knife; two iron buckles and presumed fragment of a third buckle; a pair of spurs; fragment from (maybe) an iron strap-end; three small iron fragments.

Anthropological characteristics: skeletal remains of an adult male (according to the pelvis); 35–40 years old (according to dental abrasion and structure of facies symphysialis); stature 169.0 cm (according to femur dx.); postcranial skeleton exhibits palaeopathological alterations on vertebrae (osteophytic lipping, spinal disc herniation, Schmorl's nodes); deformation of the left collarbone is caused by a malunion fracture.

Literature: (36, 47).

<u>H 129</u>

Excavation year: 1977

Square: C69-98

Stratigraphic relation: individual JP/129 was buried in the defunct sunken-featured building No. 205 – the skeletal remains rested about 11–15 cm above the bottom of the pit dwelling, along its north-eastern wall; the head originally lied across the remnants of a stone oven in the eastern corner of the building; it is probably an impious deposition of human remains.

Burial pit: not detected.

Buried individual – sex, orientation and position: male; orientation towards SE–NW; position of skeletal remains: prone; the skull is flattened, resting on the right temple; right upper extremity is stretched at an acute angle from the body; left upper extremity is bent at a right angle at the elbow, hand bones are placed below the lumbar spine; incomplete lower extremities are stretched, slightly straddled.

Grave goods: none.

Anthropological characteristics: skeletal remains of an adult male (according to the pelvis); 35–40 years old (according to dental abrasion); stature 166.7 cm (according to femur dx.); Os frontale – deformation of the left upper orbital rim, despite partial post-mortem damage there are visible healed arched lines, which are moderately sunken and go out approximately from one place very close to the medial rim of the orbit, it probably is a healed fracture (maybe an injury from a blunt object); Radius sinister – small extension of the distal end of the bone with moderate dorsal shift of the distal fragment – unverified fracture.

Literature: (36, 48).

H 207

Excavation year**: 1991**

Square: C97-53

Stratigraphic relation: the southwest shorter side of the grave pit H 207 disturbed the cut of the well (settlement feature O545); the bottom of the grave pit partially sank into the fill of the older settlement feature;

Burial pit: rectangular in plan; dimensions: 200 x 68 cm; depth 25 cm from the level of the lowered surface; pit orientation SW-NE; profiles: not recorded; bottom: flat.

Buried individual – sex, orientation and position: male; orientation towards SW–NE; position of skeletal remains: not discovered; the skull was dislocated, sinking into the fill of the settlement feature 454, lying on the right side and facing the shorter northeast side of the grave pit; the position of the skeleton cannot be determined - the postcranium is missing (except for a few ribs and vertebrae).

Grave goods: none.

Anthropological characteristics: skeletal remains of an adult male (according to the aDNA); age over 55 years (according to dental abrasion).

Literature: (36, 38).

Locality Břeclav – Pohansko, SV předhradí (North-Eastern Suburb):

H 205

Excavation year**: 2013**

Square: B62-41

Stratigraphic relation: a small child (newborn) was deposited in the fill of an Early Slavic storage pit - settlement feature No. 174; the deposit of human bones is marked as grave H 205.

Burial pit: not recorded; an irregular oval pit was created artificially around the bones of the child; it was located at a depth of 60 cm from the level of the uncovered subsoil; the skeleton was located in the southeastern half of the storage pit, at a level below the neck of the pit; dimensions of the artificial grave pit: 43 × 18 × 60 cm.

Buried individual – sex, orientation and position:child, age NB / Infans I (max. 2 weeks); orientation and position of the body undifferentiated; non-anatomically arranged remains of the skeleton of a newborn in the fill of the storage pit (the skeleton was partially disturbed by bioturbation and also during archaeological research).

Grave goods: none.

Anthropological characteristics: –

Literature: (39).

Locality Břeclav – Líbivá:

<u>Grave 5</u>

Excavation year: 1996

Square: 2

Burial pit: rectangular in plan; sections – not examined; bottom – not examined; dimensions: 183 x 69 x 167 cm.

Buried individual – sex, orientation and position: female; orientation towards SWW–NEE; position of skeletal remains: supine; the skull resting on the occipital region; upper and lower extremities in stretched position.

Grave goods: two bronze earrings, the loop is terminated on one side with a square (polyhedral) plate; iron knife; amorphous, heavily corroded bimetallic fragment.

Anthropological characteristics: skeletal remains of a young female; 18–20 years old; stature about 161.4 cm; the skeleton exhibits distinct traces of heavy physical load.

Literature: (43).

<u>Grave 8</u>

Excavation year: 1996

Square: 11, 1

Stratigraphic relation: northern part of Grave 8 was damaged by an early medieval sunken settlement feature.

Burial pit: rectangular in plan; sections – not examined; bottom – not examined; dimensions: 273 x 90 x 130 cm.

Buried individual – sex, orientation and position: male; orientation towards SWW–NEE; position of skeletal remains: supine; the skull resting on the left side; right upper extremity is stretched along the body; right lower extremity is stretched; left upper and lower extremities were damaged by a later sunken settlement feature.

Grave goods: large piece of stone with knapping marks (strike-a-light?); iron knife; bronze buckle; bronze fittings; amorphous iron fragments.

Anthropological characteristics: skeletal remains of an adult male; 50–60 years old; stature 172.2 cm; the skeleton exhibits palaeopathological alterations induced by bad health of the individual.

Literature: (43).

Grave 15

Excavation year: 1996

Square: 22

Burial pit: irregularly rectangular in plan; longitudinal section – not examined; cross-section – tub-shaped; bottom – not examined.

Buried individual – sex, orientation and position: undetermined individual; orientation towards NW–SE; position of skeletal remains: supine; poor preservation state of skeletal remains; recent dislocation (by ploughing) affected the whole skeleton.

Grave goods: none.

Anthropological characteristics: skeletal remains of a small child; category Infans I/II; palaeopathological finding on the internal surface of flat skull bones – evidence of suffered intracranial inflammation (meningitis?).

Literature: (49).

Grave 3

Excavation year: 1996

Square: 2

Burial pit: not recorded; grave was destroyed by heavy machinery during forest landscaping; only accumulations of bone residues were found.

Buried individual – sex, orientation and position: male; body orientation and position was not able to record.

Grave goods: none.

Anthropological characteristics: skeletal remains of an adult male; 25–30 years old.

Literature: (43).

Grave 6

Excavation year: 1996

Square: grave H6 was located between the old excavation trench from 1995 and the explored area in 1996, about 2 m from the southwestern sector of the square No. 2.

Stratigraphic relation: overlapping of the older grave H7.

Burial pit: rectangular in plan; dimensions: 204 x 70 cm; depth 16 cm from the level of the lowered surface; pit orientation SW-NE; profiles: not recorded; bottom: flat.

Buried individual – sex, orientation and position: male; orientation towards SW–NE; position of skeletal remains: supine; right upper extremity was strongly flexed, lying on the chest; left upper extremity was slightly flexed to the pelvis; lower extremities extended; dislocation of the chest and pelvis bones.

Grave goods: on the left side of the skull: two pieces of the iron items (small chisel? or nail?, fragment of buckle), chipped stone item; under the right ulna: iron artefact, chipped stone item; in the pelvis – iron knife, chipped stone item; at the left foot on the left side: ceramic sherd, chipped stone item.

Anthropological characteristics: skeletal remains of an adult male; 40–50 years old; stature about 170 cm.

Literature: (43).

Grave 9

Excavation year: 1996

Square: 11/12.

Stratigraphic relation: grave was disturbed by the great Moravian / early medieval settlement feature 25.

Burial pit: rectangular in plan; dimensions: 217 x 73 cm; depth 74 cm from the level of the lowered surface; pit orientation NW-SE; profiles: not recorded; bottom: flat.

Buried individual – sex, orientation and position: female; orientation towards NW–SE; position of skeletal remains: supine; right upper extremity was flexed, forearm bones on the pelvis; left upper extremity extended; lower extremities extended; chest, vertebrae, pelvis, hand and foot bones were decomposed.

Grave goods: on the right side of the skull: bronze earring; by the right humerus: bronze clasp; by the right hand: iron bracelet; in the pelvis – iron buckle; by the left femur: fragments of iron knife.

Anthropological characteristics: skeletal remains of an adult female; 30–40 years old.

Literature: (43).

Grave 13

Excavation year: 1997

Square: 15, northeastern part

Stratigraphic relation: none.

Burial pit: rectangular in plan; pit orientation W–E; profiles: trapezoid shaped; bottom: flat.

Buried individual – sex, orientation and position: ?; orientation towards W–E; position of skeletal remains: likely supine (skeleton was dislocated); right upper extremity was probably extended; left upper extremity likely flexed to the pelvis; bones of chest, pelvis and legs are incomplete.

Grave goods: none.

Anthropological characteristics: not analysed.

Literature: –

Lab ID	Site	Country	Grave Number
LIB2	Břeclav – Líbivá	Czech Republic	Hrob 8
LIB3	Břeclav – Líbivá	Czech Republic	Hrob 5
LIB4	Břeclav – Líbivá	Czech Republic	Hrob 3
LIB5	Břeclav – Líbivá	Czech Republic	Hrob 9
LIB7	Břeclav – Líbivá	Czech Republic	Hrob 6
LIB11	Břeclav – Líbivá	Czech Republic	Hrob 15
LIB12	Břeclav – Líbivá	Czech Republic	Hrob 13
POH3	Pohansko – Lesní hrúd	Czech Republic	H 6
POH11	Pohansko – Lesní školka	Czech Republic	H 25
POH13	Pohansko – Lesní školka	Czech Republic	H 32
POH27	Pohansko – Jižní Předhradí	Czech Republic	H 38
POH28	Pohansko – Jižní Předhradí	Czech Republic	H 42
POH36	Pohansko – Jižní Předhradí	Czech Republic	H129
POH39	Pohansko – Jižní Předhradí	Czech Republic	H 207
POH40	Pohansko – Lesní školka	Czech Republic	H 23

POH41	Pohansko – Lesní školka	Czech Republic	H 29
POH44	Pohansko – Pohřebiště U Kostela	Czech Republic	H174
PJP010	Pohansko - Severovýchodní Předhradí	Czech Republic	H 205

Table S1: Newly sequenced individuals and grave numbers.

2.5 Dating

The grave goods at Líbivá indicate that the furnished burials belong to the early stages of the Migration Period, conventionally dated to the 5th century CE (Additional File 2: Screening Results, 43, 44) but the graves without artifacts cannot be dated by archaeological methods, especially since it is known that the occupation of the region of Líbivá site continued into the early Middle Ages. In Pohansko, most of the inhumations from which samples were taken have been archaeologically dated to the 9th century CE (32, 38, 50). The only exception is the skeleton of a newborn child (grave H 205), which was found in the fill of the storage pit No. 174 together with the ceramics from the 7th/8th century CE (39).

According to radiocarbon dating results (Table S2 and S3, Fig. S2, S3 and S4), five of the graves sampled from Líbivá date from the 3rd to 6th century CE and two from the 7th to 9th century CE. Graves from Pohansko date to the Early Middle Ages, mostly from the 8th to 10th century CE. Only the grave H 205 of the new-born child, which is currently the oldest known human skeleton directly related to the material culture associated with the Slavs (in this case, to the pottery and the settlement remains), dates back to the 7th–8th century. The pottery complex from the fill of the settlement pit, where the child's skeleton was found, consists of undecorated and decorated vessels (Fig. S5). Typologically, these ceramics belong to the third phase of the Prague Culture in Moravia according to D. Jelínková (51) or to the R. III phase in Bohemia according to N. Profantová and M. Kuna, dated between 600 and 680 CE (52). According to G. Fusek, the temporal scope of Prague culture, which is synonymous with the Early Slavic period, spans the extended period from approximately 500 to 700 AD (see Fig. 1 in the main text) in Slovakia and neighbouring regions (53).

We ordered the two studied populations using *OxCalc* into a chronological sequence (54). Bayesian modeling of multi-site radiocarbon dataset provides tight chronometric support for the transition between Migration (Germanic-associated) and Early Mediaeval (Slavs-associated) burials. A new function *KDE_Plot* in *OxCal* has been applied to provide KDE plots, which summarize the distribution of events within our two groupings in a way that both retains signal and suppresses noise (55) (Fig. S2). KDE models are generally better suitable for summarizing radiocarbon data than the summed probability models, which contain significant calibration noise due to plateau in the calibration curve, specifically between CE 600 and 1000 (56).

The final chronological model of the transition between Germanic- and Slavic-associated burials, comprising 22 samples from 18 graves, offers good *OxCal* diagnostic statistics with Amodel values of 102.8%. The result of the modeling (at a 95.4% probability level, 2-sigma range) is that the Germanic-associated burials from Líbivá ended between 421 and 589 calAD, whereas the radiocarbon dates from Pohansko and Líbivá indicate that the Early Mediaeval Slavic-associated inhumations began between 659 and 775 calAD and continued until 900 and 1024 calAD (Fig. S3 and S4). These intervals coincide with historical milestones. The people buried in Líbivá could be residues of local Germanic Suebi, who survived in Moravia after the majority of Suebi migrated to Hispania in the early 5th century, where they founded in 406 CE an independent kingdom (1, 44, 57). Slavs appeared in Central Europe already in the second half of the 6th century CE, after the last Germanic-speaking population of Longobards had left their territory in (present-day) Bohemia, Austria, Slovakia and Hungary in 568 CE and settled in northern Italy in present-day Lombardy (6). Nevertheless, the funerary ritual of the Early Slavs was cremation, which changed to inhumation only in the late 8th and 9th centuries CE (37).

	Site	Country	Grave Number	Lab. no.	Age 14C	var	%N	%C	%coll
LIB2	Břeclav – Líbivá	Czech Republic	H008	Poz-108443	1570	30	1,7	5	3,5
LIB3	Břeclav – Líbivá	Czech Republic	H005	Poz-109465	1550	30	4,2	9,9	6,3
LIB4_1	Břeclav – Líbivá	Czech Republic	H003	ULA-9314	1625	15	9,3	27,6	1,2
LIB4_2	Břeclav – Líbivá	Czech Republic	H003	VERA-1921	1570	40	-	-	-
LIB5	Břeclav – Líbivá	Czech Republic	H009	ULA-9316	1660	15	13,2	37,5	1,5
LIB7	Břeclav – Líbivá	Czech Republic	H006	ULA-9315	1190	15	13,8	39,4	2,1
LIB11_1	Břeclav – Líbivá	Czech Republic	H015	Poz-108442	1245	30	3,5	9,7	9
LIB11_2	Břeclav – Líbivá	Czech Republic	H015	Poz-112114	1280	30	2	4,2	6,4
LIB12	Břeclav – Líbivá	Czech Republic	H013	MAMS-35201	1616	17	8,52	19,6	4,6
Not analyzed	Břeclav – Líbivá	Czech Republic	H014	ULA-9317	1630	15	12,6	36,2	1,6

POH3	Pohansko – Lesní hrúd	Czech Republic	H006	Poz-108493	1085	30	1,4	5,1	3,8
POH11	Pohansko – Lesní školka	Czech Republic	H025	Poz-108485	1230	30	1,9	7,2	6,7
POH13	Pohansko – Lesní školka	Czech Republic	H032	Poz-108492	1155	30	2	7	5,8
POH27	Pohansko – Jižní Předhradí	Czech Republic	H038	Poz-108486	1120	30	1,7	6,1	5,7
POH28_1	Pohansko – Jižní Předhradí	Czech Republic	H042	Poz-87831	1205	30	3,7	11,2	7,8
POH28_2	Pohansko – Jižní Předhradí	Czech Republic	H042	Poz-87832	1165	30	4,5	14,4	10,7
POH36	Pohansko – Jižní Předhradí	Czech Republic	H129	Poz-112116	1150	30	2,7	9,0	6,1
POH39	Pohansko – Jižní Předhradí	Czech Republic	H207	-	-	-	-	-	-
POH40	Pohansko – Lesní školka	Czech Republic	H 23	Poz-108489	1105	30	4,5	11	9,2
POH41	Pohansko – Lesní školka	Czech Republic	H029	Poz-108491	1160	30	3	9	10,4
POH44_1	Pohansko – Pohřebiště U Kostela	Czech Republic	H174	Poz-101781	1205	30	0,9	13,6	6,5
POH44_2	Pohansko – Pohřebiště U Kostela	Czech Republic	H174	Poz-101782	1200	30	2,1	12,9	10
PJP010	Pohansko – Severní předhradí	Czech Republic	H205	ULA-9520	1295	15	15,7	42,5	14,6

Table S2: Results of radiocarbon dating.

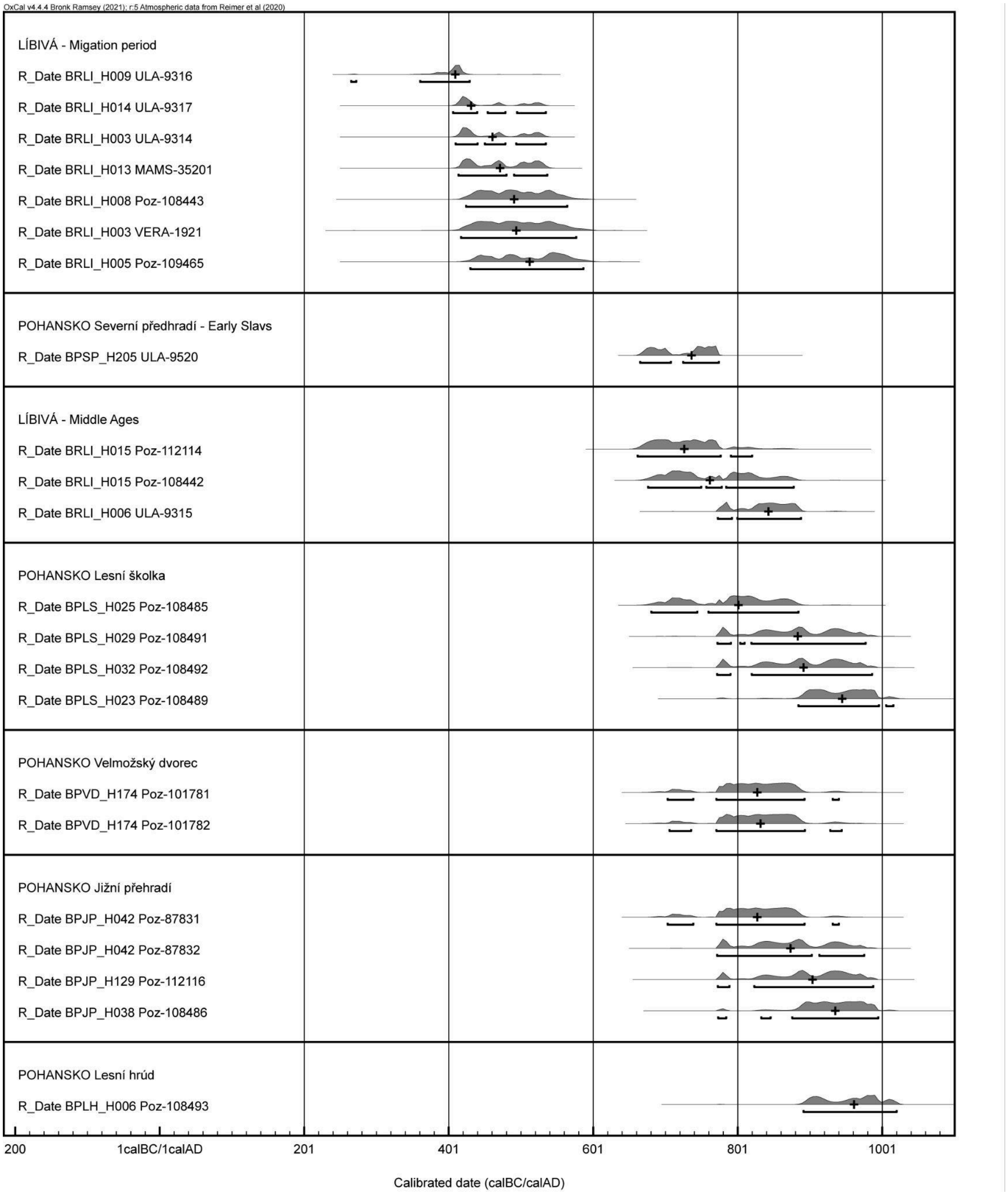

Fig. S2: Calibrated dates probabilities for every sample tested.

Name	Laboratory code	Unmodeled (BC/AD)		Modeled (BC/AD), Amodel 102.8, Aoverall 101.9		Indices	
		from_95_4	to_95_4	from_95_4	to_95_4	A	C
Boundary Start LÍBIVÁ_Migration_period				328	523		98,70
KDE_Plot Migration_period LÍBIVÁ							99,60
LÍBIVÁ_Migration_period							
R_Date BRLI_H009	ULA-9316	266	430	384	527	101,00	99,70
R_Date BRLI_H014	ULA-9317	407	535	407	531	120,30	99,80
R_Date BRLI_H003	ULA-9314	410	535	410	531	119,50	99,80
R_Date BRLI_H013	MAMS-35201	414	537	412	531	109,00	99,80
R_Date BRLI_H008	Poz-108443	425	565	416	533	95,20	99,30
R_Date BRLI_H003	VERA-1921	418	577	413	533	98,20	99,30
R_Date BRLI_H005	Poz-109465	431	587	417	548	80,80	99,40
Boundary End LÍBIVÁ_Migration_period				421	589		97,50
Sequence							
Boundary Start POHANSKO_LÍBIVÁ_Early_Middle_Ages				659	772		98,90
KDE_Plot Early_Middle_Ages POHANSKO_LÍBIVÁ							99,90
POHANSKO Severní předhradí		Early Slavs					
R_Date BPSP_H205	ULA-9520	666	775	685	778	111,10	99,90
LÍBIVÁ-Early Middle Ages							

R_Date BRLI_H015	Poz-112114	662	821	684	873	87,00	99,70
R_Date BRLI_H015	Poz-108442	677	878	707	881	89,50	99,70
R_Date BRLI_H006	ULA-9315	773	888	773	888	98,70	99,80
POHANSKO Velmožský dvorec							
R_Date BPVD_H174	Poz-101781	704	941	713	938	105,00	99,90
R_Date BPVD_H174 Poz-101782		706	945	770	944	104,40	99,90
POHANSKO Jižní předhradí							
R_Date BPJP_H042	Poz-87831	704	941	714	937	104,90	99,90
R_Date BPJP_H042	Poz-87832	772	976	772	955	102,40	99,90
R_Date BPJP_H129	Poz-112116	773	988	772	974	97,70	99,80
R_Date BPJP_H038	Poz-108486	774	995	775	987	96,30	99,80
POHANSKO Lesní školka							
R_Date BPLS_H025	Poz-108485	681	885	709	885	103,30	99,80
R_Date BPLS_H029	Poz-108491	773	978	772	960	101,00	99,80
R_Date BPLS_H032	Poz-108492	772	987	773	969	99,40	99,80
R_Date BPLS_H023	Poz-108489	885	1016	778	991	98,10	99,50
POHANSKO Lesní hrúd							
R_Date BPLH_H006	Poz-108493	892	1021	886	991	95,80	99,70
Boundary End POHANSKO_LÍBIVÁ_Early_Middle_Ages				900	1024		99,00

Table S3: Unmodeled and modeled year of death for the tested samples.

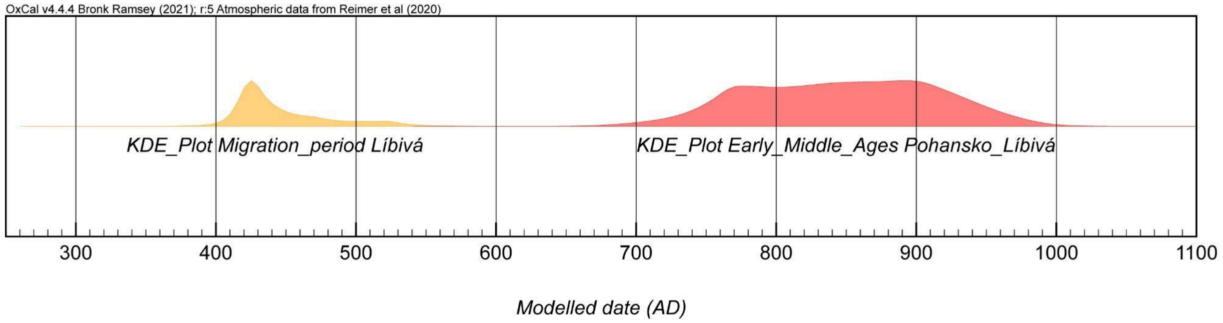

Fig. S3: Summary probabilities of modeled dates for all individuals from the given population.

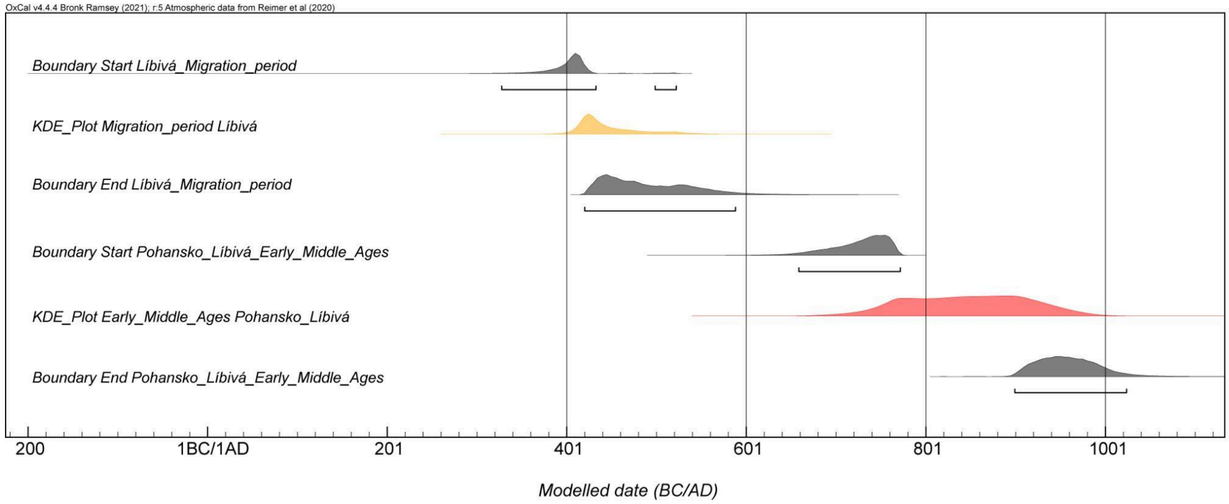

Fig. S4: Summary probabilities of modeled dates for all individuals from the given population and boundary starts and ends for the modeled summary dates.

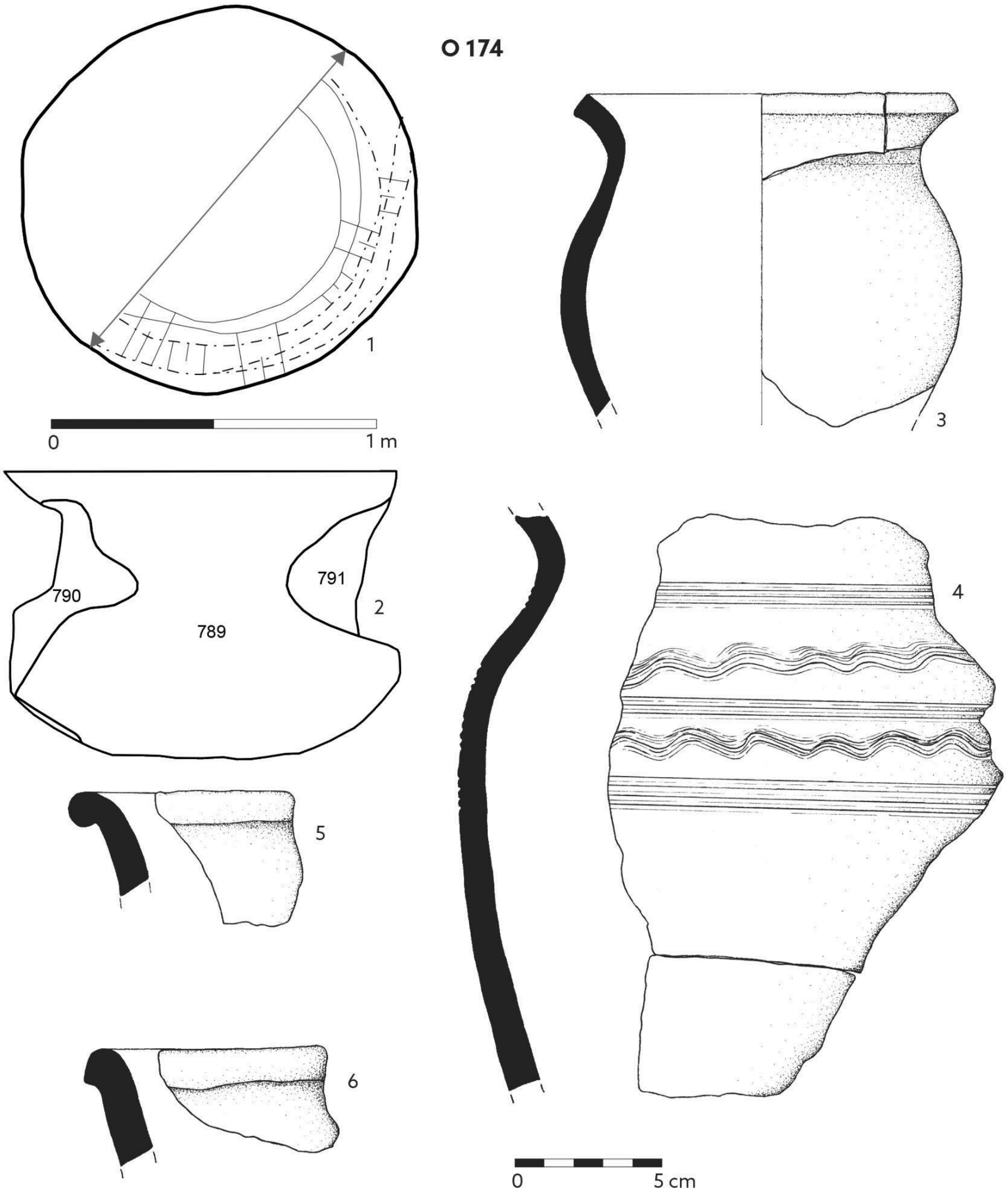

Fig. S5: The settlement pit No. O 174, where the grave H 205 of the new-born child was found, and the pottery associated with this pit.

3. Genetic analysis

The following versions of software tools were used, unless stated otherwise:

- *fastqc* - version 0.11.5, (www.bioinformatics.babraham.ac.uk/projects/fastqc/)
- *Trim Galore!* - version 0.6.5, (https://github.com/FelixKrueger/TrimGalore)
- *bwa - Burrows-Wheeler Alignment Tool* - version 0.7.17 (58)
- *SAMtools* - version 1.9 (59)
- *Picard-tools* - version 2.21.1, http://broadinstitute.github.io/picard/
- *GATK* - version 3.8 (60)
- *ATLAS* - version 1.0, commit 06d1209
- *ContamMix* - version 1.0 (61)
- *MIA (Mapping Iterative Assembler)* - version 1.0 https://github.com/mpieva/mapping-iterative-assembler
- *ANGSD* - version 0.917 (62)
- *GLIMPSE* - version 1.1.0 (63)
- *ChromoPainter* - version 2 (64)
- *fineSTRUCTURE* - version 0.0.5 (64)
- *MOSAIC* - version 1.3.7 (65)
- *irlba* - version 2.3.1 (https://github.com/bwlewis/irlba)
- *BeXY* - version 1.0, commit ac0d333 (66)

3.1 aDNA authentication

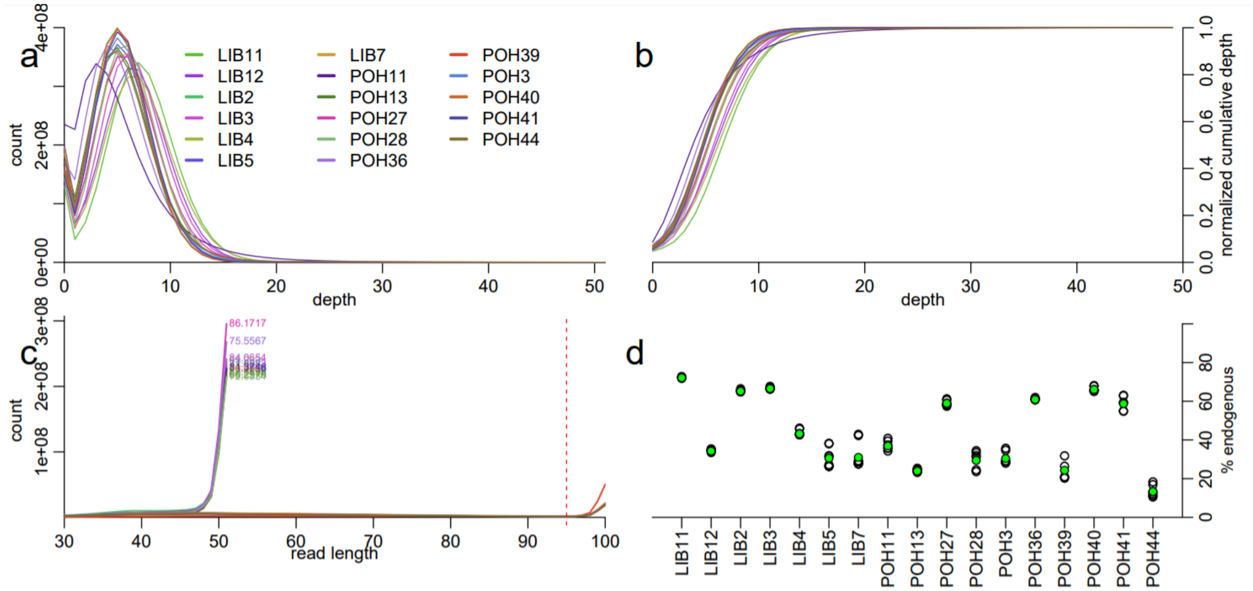

Fig. S6: Sample stats. a) Read depth distribution b) normalized cumulative depth distribution c) Read length distribution. Paired-end sequenced samples were sequenced up to 50 base pairs, while single-end samples were sequenced up to 100 base pairs. The vertical dashed red line indicates where single-end sequenced read-groups were split for post-mortem damage estimation. Longer reads are assumed to not have been sequenced to their full length and miss the PMD pattern on the 3' side. Fragment lengths of paired-end samples are indicated as numbers within the plot. d) percentage of endogenous reads among sequenced reads for libraries (black circles) and pooled whole samples (green circles), respectively.

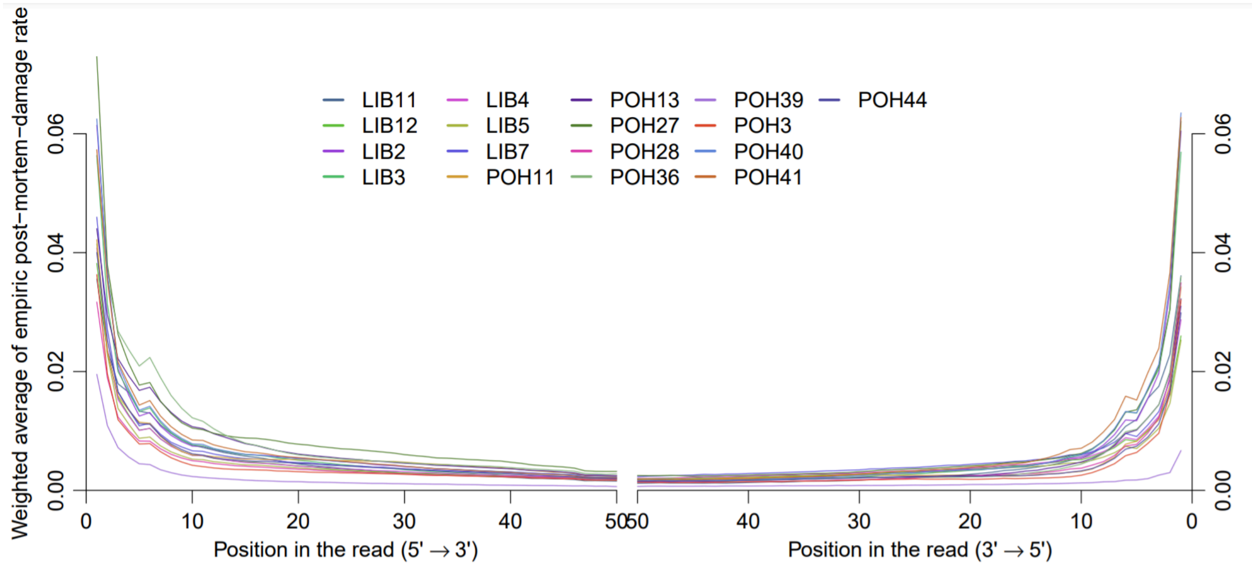

Fig. S7: Post-mortem damage patterns for newly sequenced genomes. All samples show elevated PMD towards the ends of the fragments. Partial UDG-treatment was performed on all samples For POH39, more of the libraries were treated with UDG, resulting in a lower PMD pattern.

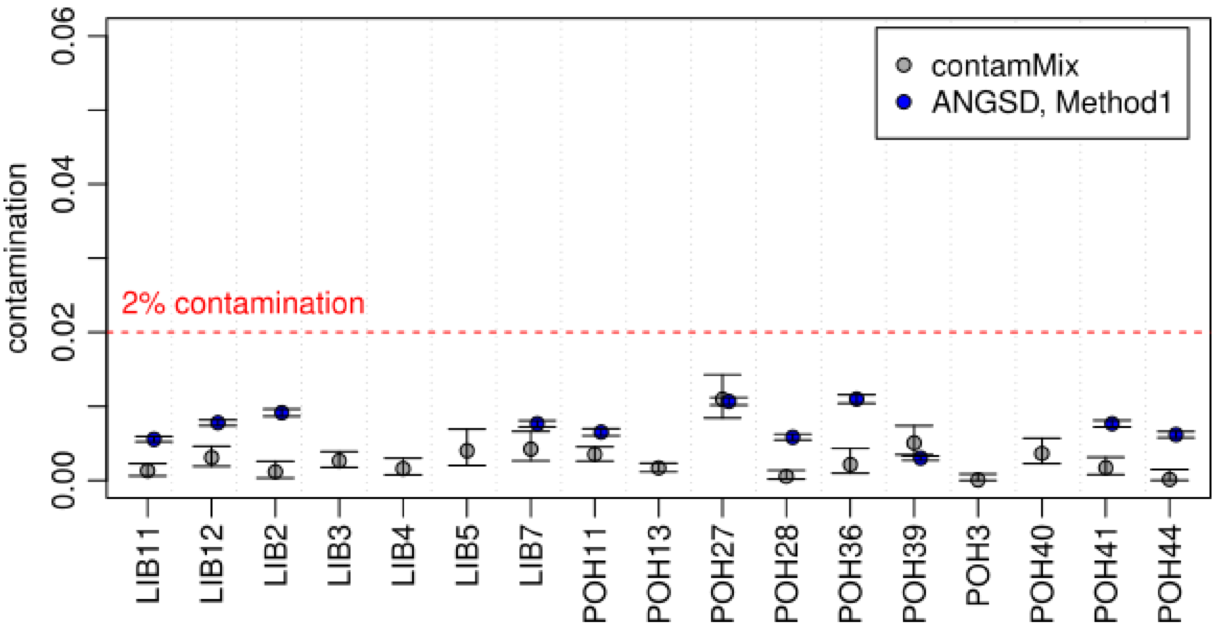

Fig. S8: Contamination estimation. Based on mitochondrial regions (*contamMix*, grey) and X-chromosomal regions for male samples (*ANGSD*, Method1, blue). All samples show less than 2% of contamination.

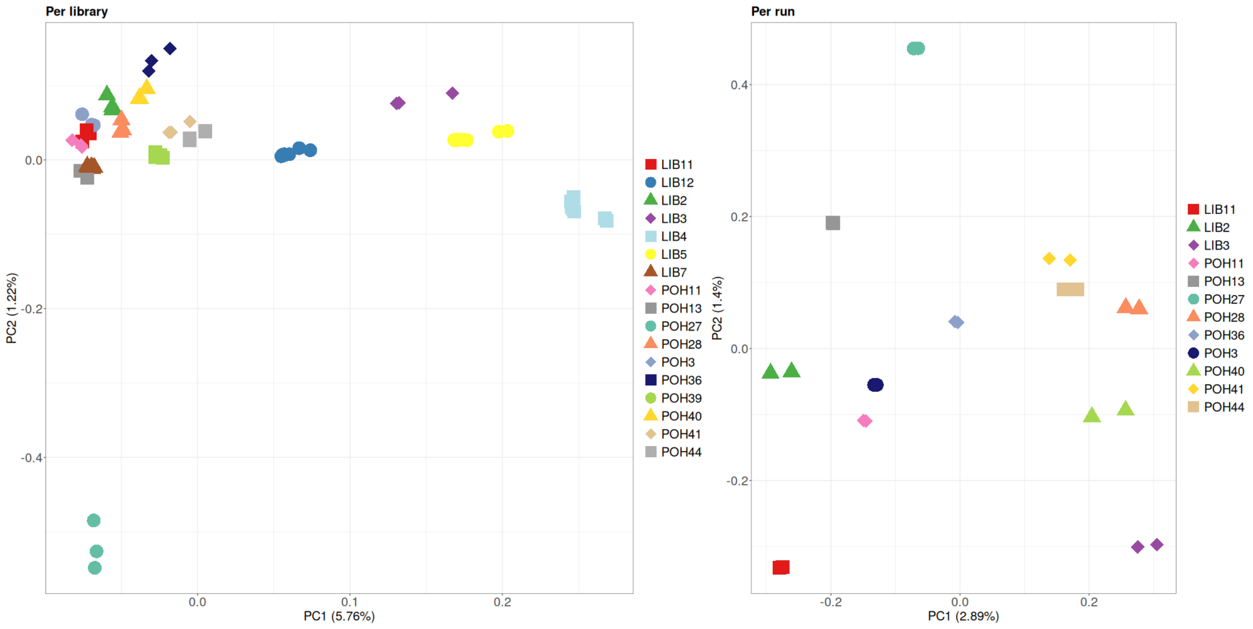

Fig. S9: Principal component analysis per library and per sequencing run, to verify sample integrity. Differences between samples are generally larger than differences among samples.

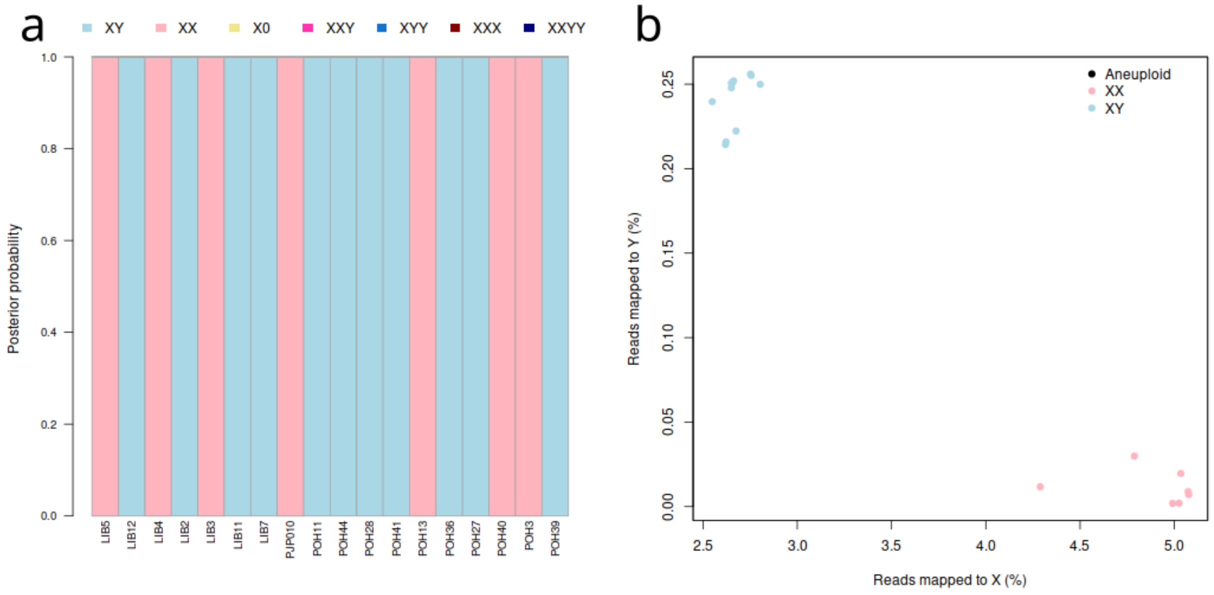

Fig. S10: Inferred genetic sex. a) Posterior probabilities for different sex karyotypes as obtained with *BeXY*. b) Fractions of reads that map to the X- and Y-chromosomes for all individuals

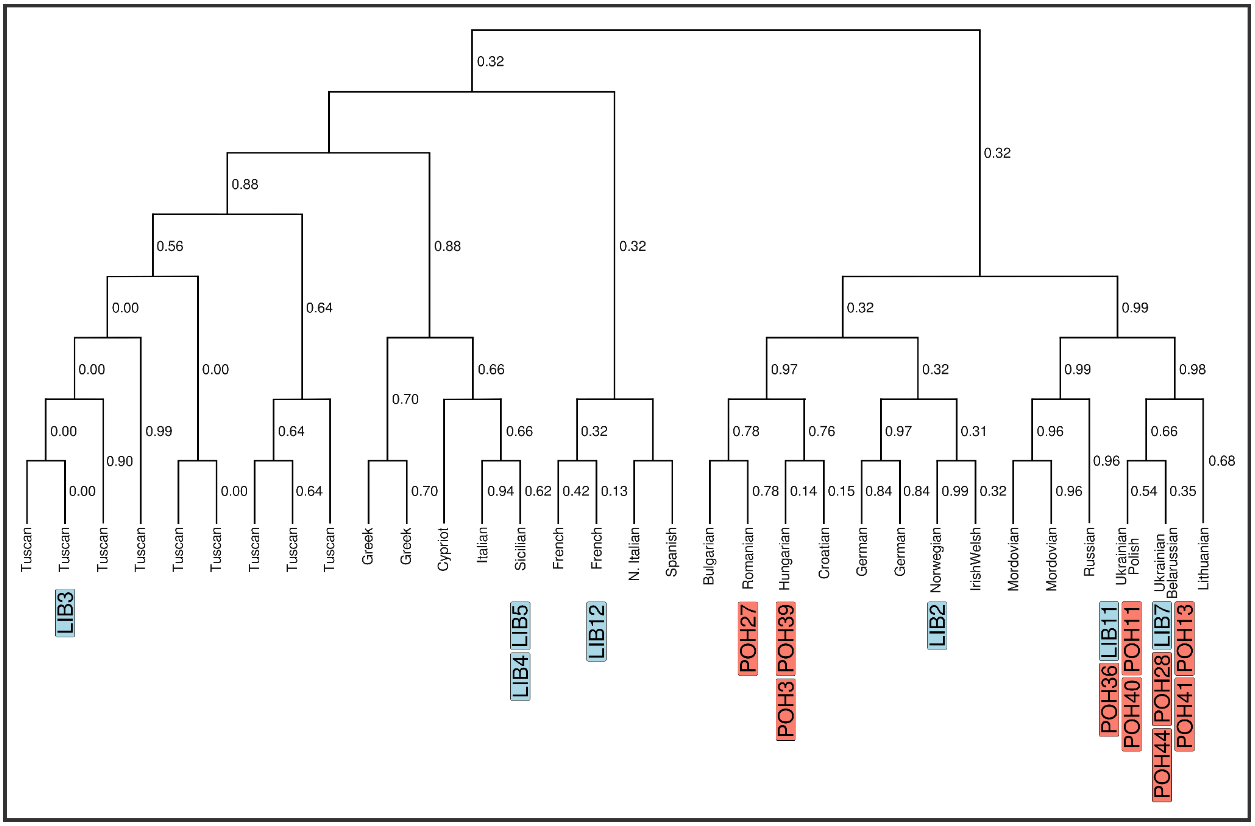

Fig. S11: Population dendrogram generated by the *fineSTRUCTURE* tree building algorithm. Labeled tips refer to the primary population(s) represented in that clade. Migration period individuals superimposed in blue and Early Middle Age samples superimposed in red.

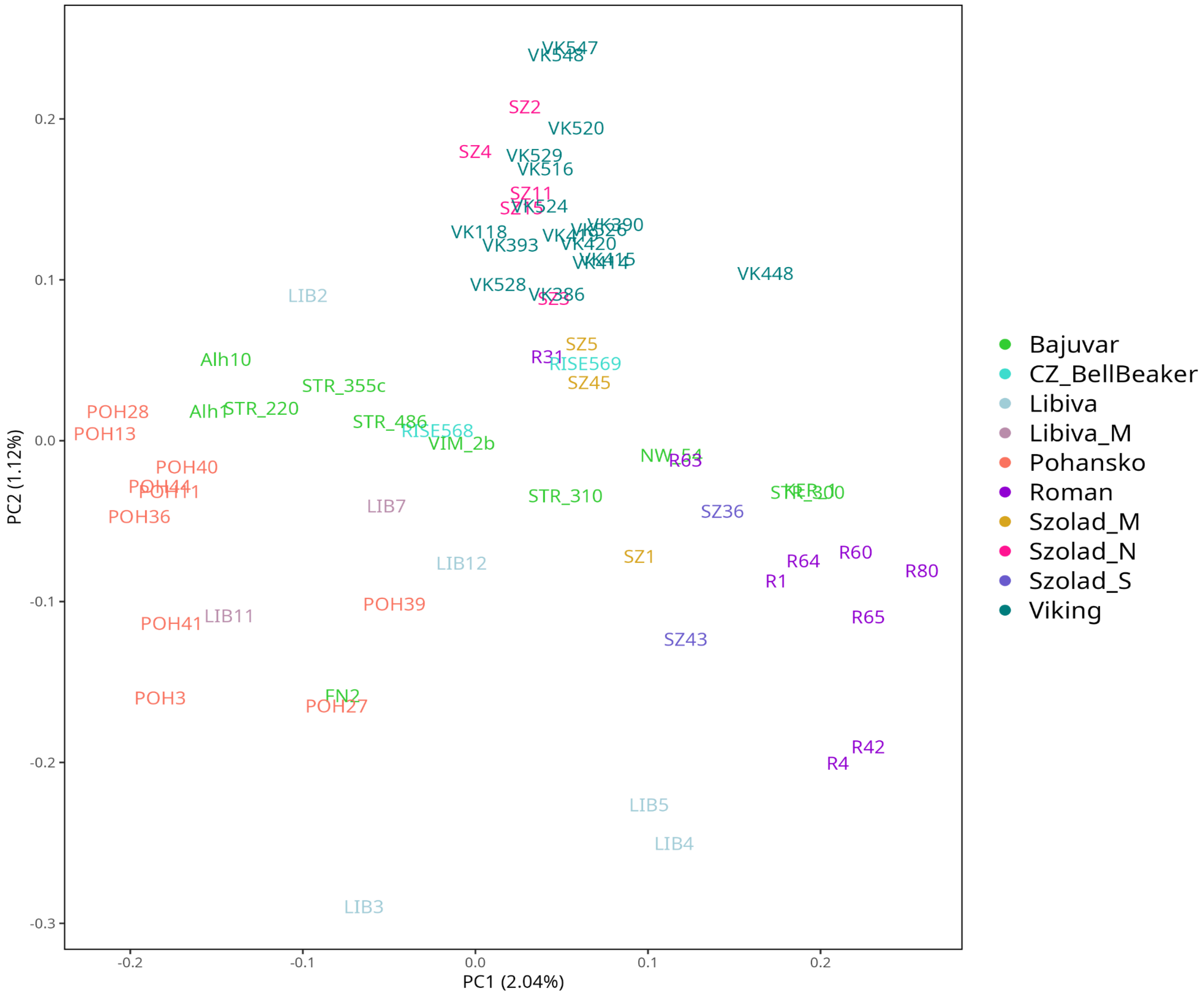

Fig. S12: *PCAngsd* PC1 vs PC2: *PCAngsd* preserves the cline of 5th century Líbivá, with apparent north affinity of LIB2. LIB7 could also be part of the cline here, however, due to chunklength PCA and radiocarbon dating, the Early Medieval origin of this individual can be assumed. Pohansko is less homogenous than on projected PCA, but clearly separated from the Lombard period cline.

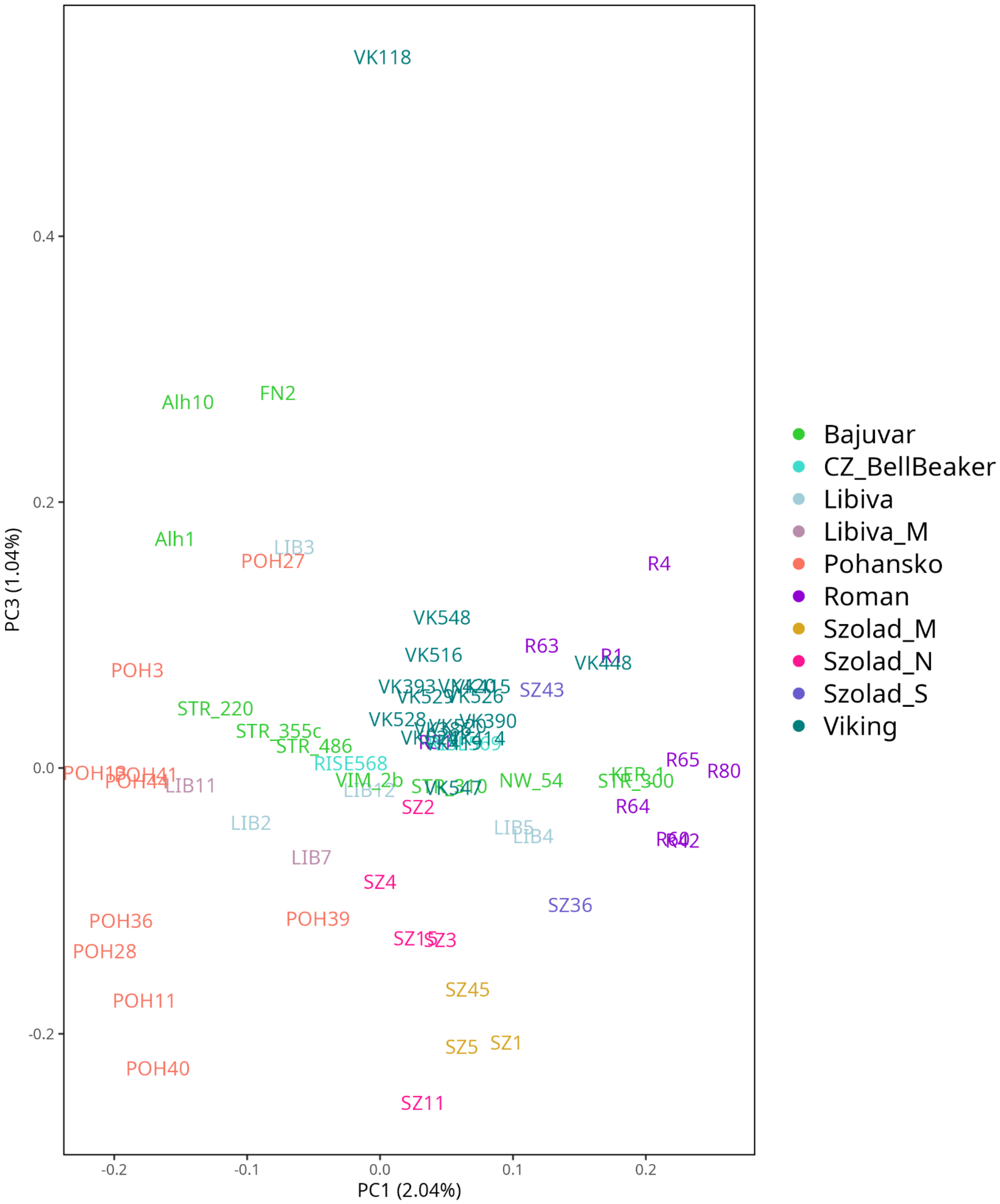

Fig. S13: *PCAngsd* PC1 vs PC3

3.2 qpWave and qpAdm

Genetic ancestry modeling was performed using the *qpWave* and *qpAdm* functions of the *admixr* package (67) in *R* (v4.0.5 (68)) utilizing *ADMIXTOOLS* v702 (69). The set of outgroups included representatives of Eurasian and relevant non-Eurasian ancient lineages: *West_Africa_ancient, Tianyuan*, *Iron_Gates_HG*, *Levant_N*, *Anatolia_N*, *Iran_N*, *Yamnaya_Samara*, *Alalakh_MLBA*, *Iberia_IA*, *Mycenaean*, *Steppe_IA*. Results are in the Additional file 3: Dataset S4, S5 and S6.

To get a better understanding of population change in Moravia between the Migration Period and Early Middle Ages through *qpAdm* modeling, we formulated the following questions:

To what extent can continuity between MMP and MEM be observed?

To answer the question of continuity or discontinuity, we tested cladding of studied populations with *qpWave* which ruled out MMP as the only genetic source of MEM ($p = 9.0566163^{-56}$). This also applies when low-depth samples are added to their respective groups ($p = 1.52^{-26}$)(Additional file 3: Population Definitions). Even when MEM is split into MEM_early and MEM_late (Additional file 3: Population Definitions), neither of these groups form a clade with MMP (Additional file 3: Dataset S4).

Would there be continuity between MEM samples from different sites?

Further *qpWave* modeling showed some continuity between similarly dated Pohansko and Líbivá populations: *qpWave* test with Pohansko and a group consisting of LIB7 and LIB11 did not disprove their cladding, therefore Pohansko can be modeled with Early Medieval Líbivá on its own ($p > 0.12$), and absolute continuity between MMP and Early Medieval Líbivá populations can be disproven ($p < 10^{-16}$). Therefore they are grouped together for the population-genetic analysis in this study. The sample PJP010 dated to the late phase of Prague culture is also not cladding with the preceding MMP samples ($p < 10^{-4}$) while it is cladding with 8th and 9th century samples from Pohansko ($p > 0.74$) and Early Medieval Líbivá samples ($p > 0.28$).

In case there is no direct continuity between MMP and MEM, can MEM be modeled as a mixture of MMP and other European populations? To what extent did each such population contribute to the MEM population?

Some plausible models of the MEM population contain a contribution from MMP (Additional file 3: Dataset S5). To investigate the proportion of MMP ancestry in the MEM population, we modeled MEM as a mixture of MMP and various Bronze Age, Iron Age and Early Medieval European populations (Additional file 3: Dataset S5). Proportion of its ancestry in two-way admixture models that passed the estimated p-value (≥ 0.05) and contained only the predating populations with more than 1 individual per group ranged between 21.1% and 51.8%. Populations that could have contributed to genetic variability of MEM with MMP include *Russia_Ingria_IA, Poland_Roman, Lithuania_Marvele_Roman, Poland_Viking and Estonia_IA.* However, it is possible that MMP only represents a genetically similar population and the true source is absent from the reference populations explored. Similarly, MMP acts like a proximal source population when modelling some of the post-dating populations from the Volga-Oka region (Additional file 3: Dataset S6).

What populations could be considered sources of the MMP?

We found several two-way models explaining the genetic variability of MMP, all of them included at least one population with Southern European ancestry (Additional file 3: Dataset S5).

What is the genetic relationship between the MMP and Szólád communities that represent the Lombard period?

When exploring the ancestry of the MMP, we focused on the published groups that lie on the same cline on projected PCA. The *qpWave* results show that MMP alone does not explain the whole genetic variability of *Szólád* ($p < 10^{-11}$), however, when divided into groups labeled "north", "south" and "others" (depending on the ancestry proportions in (7)), p-value for the intermediate group "others" and MMP is 0.079 which does not reject their cladding. We found other populations that also clade with *Szólád*: *NE_Iberia_RomP, NE_Iberia_c.6-8CE_ES* and *Hungary_Sarmatian_Transtisza*. In the other direction, *Szólád* as a whole cannot be modeled with a two-way model containing MMP, however, there is a plausible model for Szólád-north containing MMP and *Russia_Ingria_IA* ($p = 0.187$). However, we note that the low number of MMP samples could impact the ancestry estimates of this group.

Other published populations from the Lombard Period include Collegno (Italy), Tesárské Mlyňany (Slovakia), and Balatonszemes, Fonyód and Hács (Hungary). *qpWave* p-values ($p < 10^{-5}$) indicate that neither of them alone explain the genetic variability of MMP (Additional file 3: Dataset S3).

3.3 Admixture analysis

Supervised *ADMIXTURE* (v.1.3.0) analysis was performed with cross validation (-cv) for multiple seeds (-s). We used populations provided by 1000Genomes as source populations. Utah residents with Northern and Western European ancestry (CEU) and British (GBR), as well as Iberians (IBS) and Toscani (TSI), were marked as different source populations for supervised admixture runs, but their respective ancestry proportions were combined afterwards for Fig. S14. The same was done for the Dai Chinese (CDX), Han Chinese (CHB), Southern Han (CHS), Japanese (JPT) and Kinh Vietnamese (KHV), whose ancestry proportions were combined into the East Asian (EAS) source population. While the populations associated with Early Slavs (Pohansko, LIB7 and LIB11) and the predating MMP group both contain Central European (CEU-GBR) as well as Southern European (IBS-TSI) ancestry, they differ in the heterogeneity of their ancestry components. Most individuals in the predating MMP group possess only one of the two main ancestry components, with the exception of LIB12, whose ancestry profile seems to match an F1 generation hybrid. The populations Pohansko and Early Medieval Líbivá demonstrate a homogenization of these ancestry components, as well as an introduction of Finnish ancestry. Slight levels of East Asian ancestry can be observed in the latest Pohansko individual, POH3, as well as individual POH39, which could not be dated. Results are in Fig. S14.

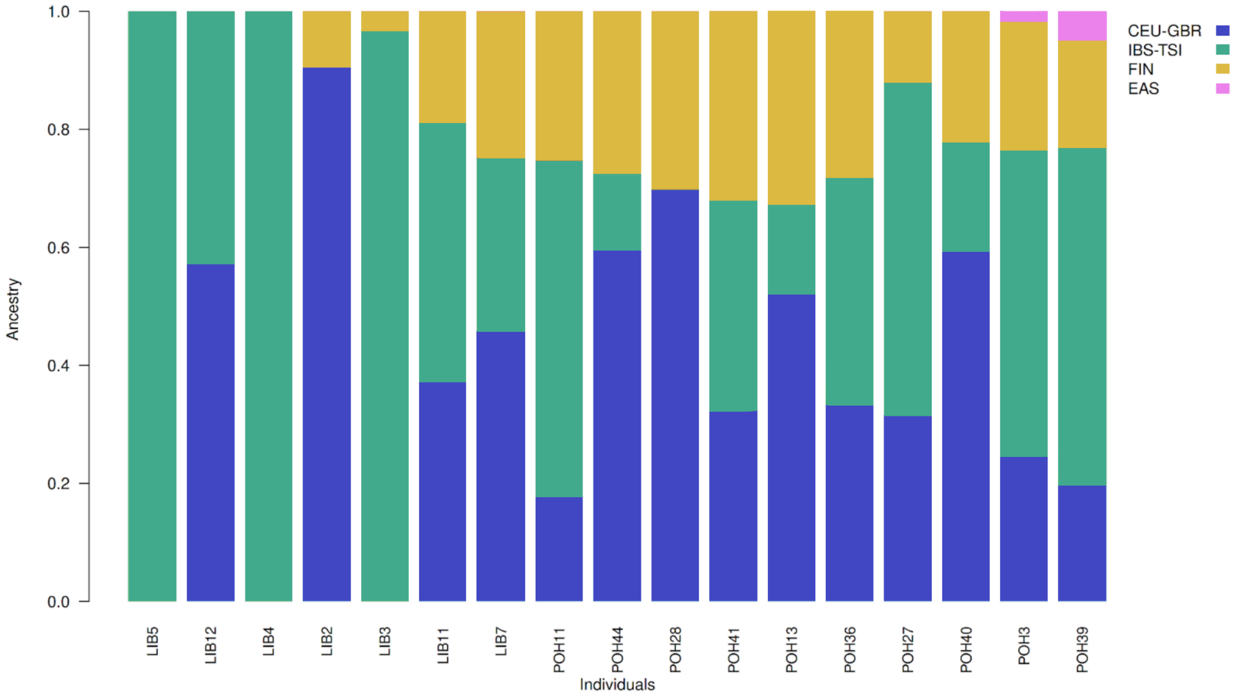

Fig. S14: Supervised *ADMIXTURE* analysis. CEU-GBR are Utah residents with Northern and Western European ancestry and British in England and Scotland, IBS-TSI are Iberians in Spain and Toscani in Italy, FIN are Finnish in Finland and EAS represents East-Asian ancestry. Overall, MMP samples have a lower proportion of the FIN component. Individuals POH3 and POH39 differ from other MMP individuals by a small EAS component.

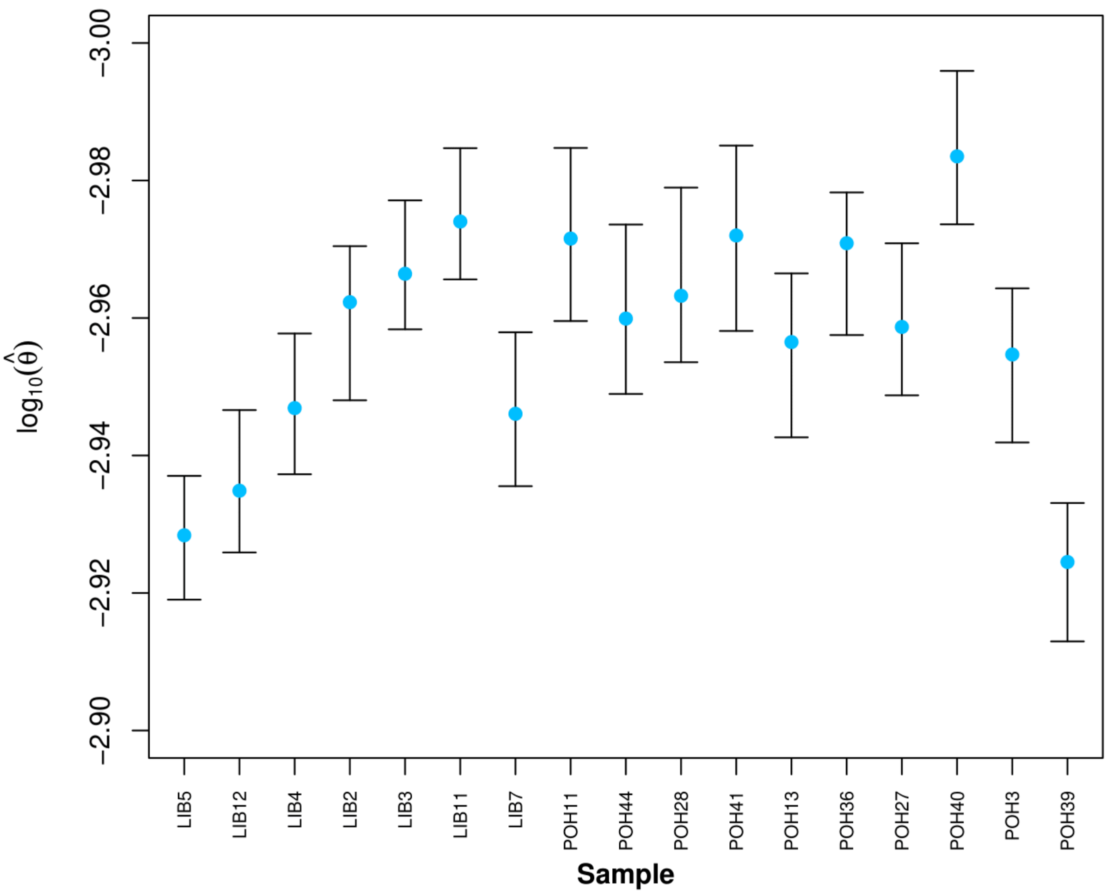

Fig. S15: Genome wide heterozygosity. There is no statistically significant difference in heterozygosity between MMP and MEM individuals. Individual POH39 was more UDG-treated which might have influenced their inferred heterozygosity levels.

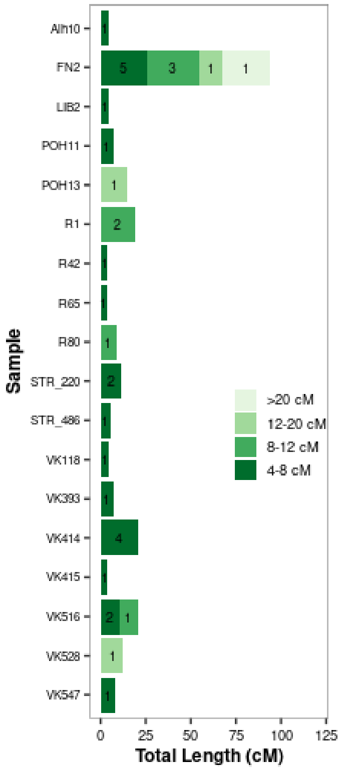

Fig. S16: Runs of homozygosity in the genome of newly analysed and reference samples. Only individuals with at least one ROH segment of minimum length 4 cM are plotted. Two individuals from Pohansko have ROHs longer than 8-12 cM, whereas only LIB2 individual, representing MMP, has ROH 4-8 cM long.

3.4 f-statistics

To explore the affinity of the studied South Moravian populations to published reference groups, the outgroup-f3- and f4-statistics analysis (70, 71) were applied with f3- and f4-functions of the *admixr* package (67) in *R* (v4.0.5 (68)) utilizing *ADMIXTOOLS* v702 (69). The population *Mbuti.DG* was chosen as an outgroup (as this population presumably shared no gene flow with the test populations).

First, we conducted outgroup-f3-statistics on MEM and MMP groups to determine what ancient European populations shared most gene flow with them (Fig. S17 and S18, Additional File 3: Dataset S2.2). We tested populations representing the genetic variability in Europe from Iron Age to Early Middle Ages. However, some regions were underrepresented due to absence of published samples, hence an older population representing the region was chosen.

Overall, both tested populations are most proximal to *Poland_Viking, Sweden_Viking, Russia_Viking* and *Lithuania_BA.* The difference between them lies mainly in MMP's greater proximity to *Collegno* and *Sardinia_BA*.

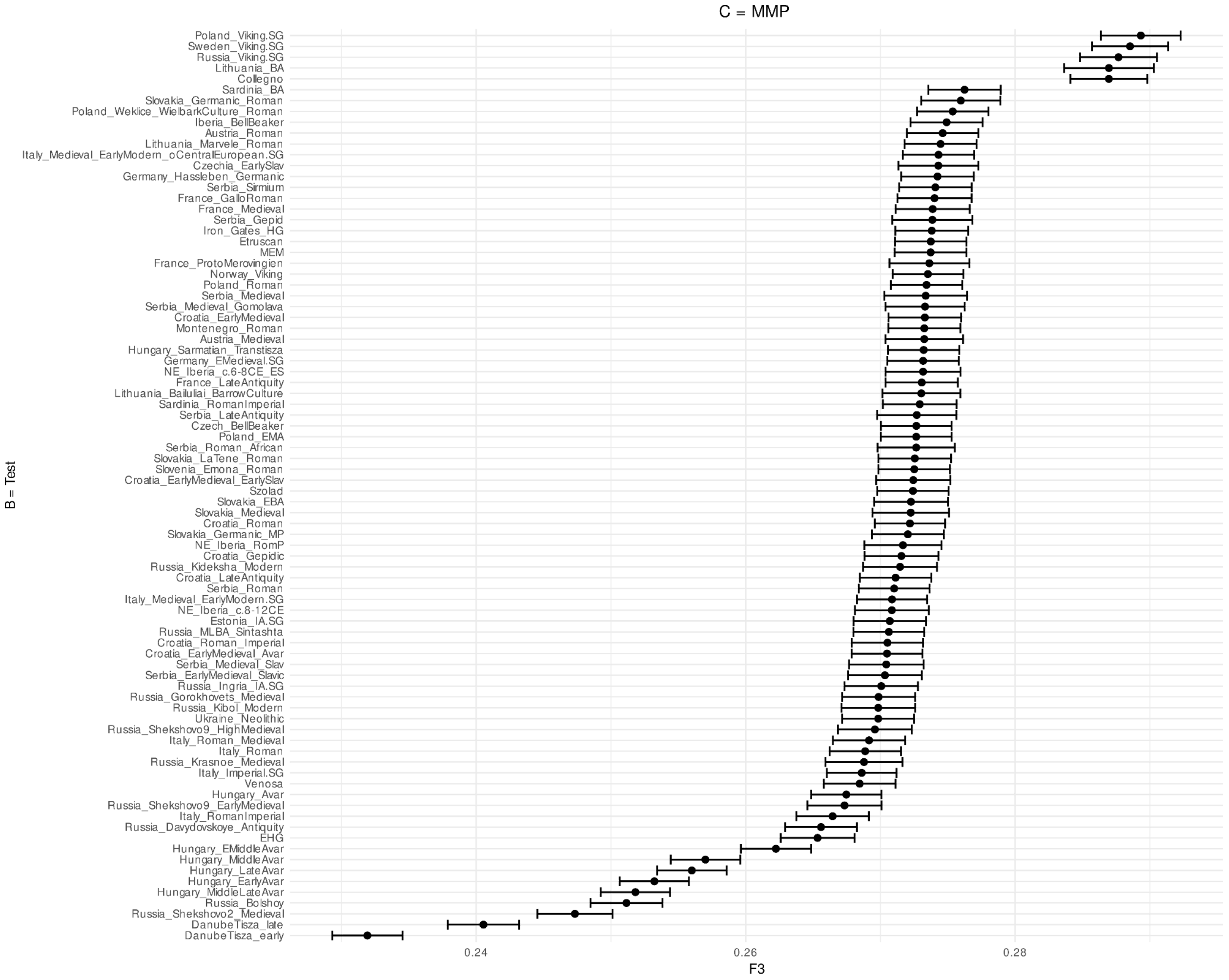

Fig. S17: Outgroup-f3-statistics, test = MMP

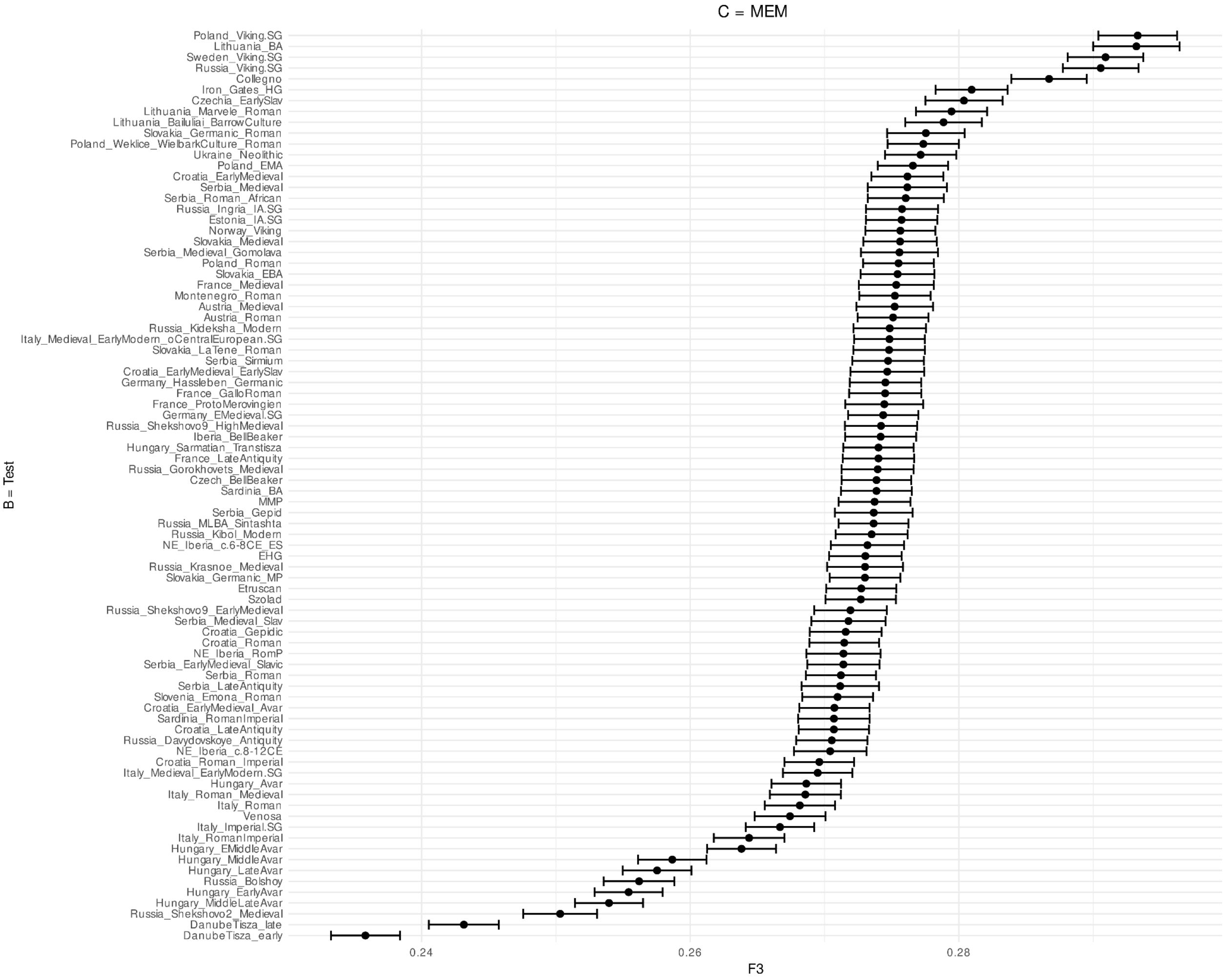

Fig. S18: Outgroup-f3-statistics, test = MEM

Analysis of f4-statistics was conducted to investigate the relationship of populations in South Moravia to each other. With respect to MMP, cladding of Pohansko and two later dating samples from Líbivá (LIB11, LIB7) or PJP010 is not broken which is in line with their common dating to Early Middle Ages and supports their grouping as MEM (Additional File 3: Dataset S3.1).

The populations that break the cladding of MEM and MMP (in a form *f4(Outgroup,test;MEM,MMP)*) are in Fig. S17. The similarity of populations from Northern and Eastern Europe to MEM supports a Northern and Eastern shift. These observations are similar when all low-depth sequenced samples are added to their respective groups (Additional file 3: Population Definitions), since groups with Eastern European ancestry break the clade in favour of MEM (Fig. S20).

f-statistics-based modeling suggests a close relationship between MEM and the early Slavic populations from Volga-Oka interfluve: the cladding of MEM with some of the early Slavic samples (*Kideksha, Kibol 3, Krasnoe 3*) is broken with only a very small number of groups (*DanubeTisza_early,*

Hungary_MiddleLateAvar, France_GalloRoman, France_Medieval, Germany_Hassleben_Germanic, Italy_Imperial, Italy_Roman, Italy_RomanImperial, Russia_Bolshoy, Russia_Shekshovo2_Medieval, Sardinia_RomanImperial) (Additional file 3: Dataset S3.5) suggesting small levels of higher Southern European ancestry in MEM and Eastern-like influences in some Volga-Oka groups.

To explore the Avar genetic influence in the region of Moravia, we conducted f-statistic tests and split Pohansko into two groups. The first one, *POH_Avar*, contains individuals with an outlying ancestry on Chunklength PCA (Fig. 2A) and Projected PCA (Fig. 2B), Asian admixture component in Supervised *ADMIXTURE* (Fig. S14) or forming a separate group on the *fineSTRUCTURE* population dendrogram (POH3+POH27+POH39). *POH_N* contains the rest of the individuals (POH11+POH13+POH28+POH36+POH40+POH41+POH44). f4 in the form of *f4*(*Outgroup,DanubeTisza_early;POH_N,POH_Avar*) does not provide a significant Z score (Z score = 1.125). This is also true when *POH_N* is substituted for *Lithuania_BA*, *Estonia_IA, Poland_EMA* or *Russia_Ingria_IA* and *DanubeTisza_early* substituted for *DanubeTisza_late, Hungary_MiddleLateAvar, Hungary_EarlyAvar, Hungary_LateAvar, Hungary_MiddleAvar, Hungary_EMiddleAvar, Hungary_Avar,* or *Croatia_EarlyMedieval_Avar*. Outgroup-f3 in the form of *f3(Outgroup, POH_N/POH_Avar, DanubeTisza_early)* show a higher attraction of *POH_Avar* to *DanubeTisza_early* than for *POH_N* though the values with standard error overlap (Additional file 3: Dataset S2.1). Overall, from f-statistics alone, it can be concluded that the Avar ancestry in some Pohansko individuals is not significantly greater than in the rest of them or the populations proximal to Pohansko (Additional file 3: Dataset S3.3).

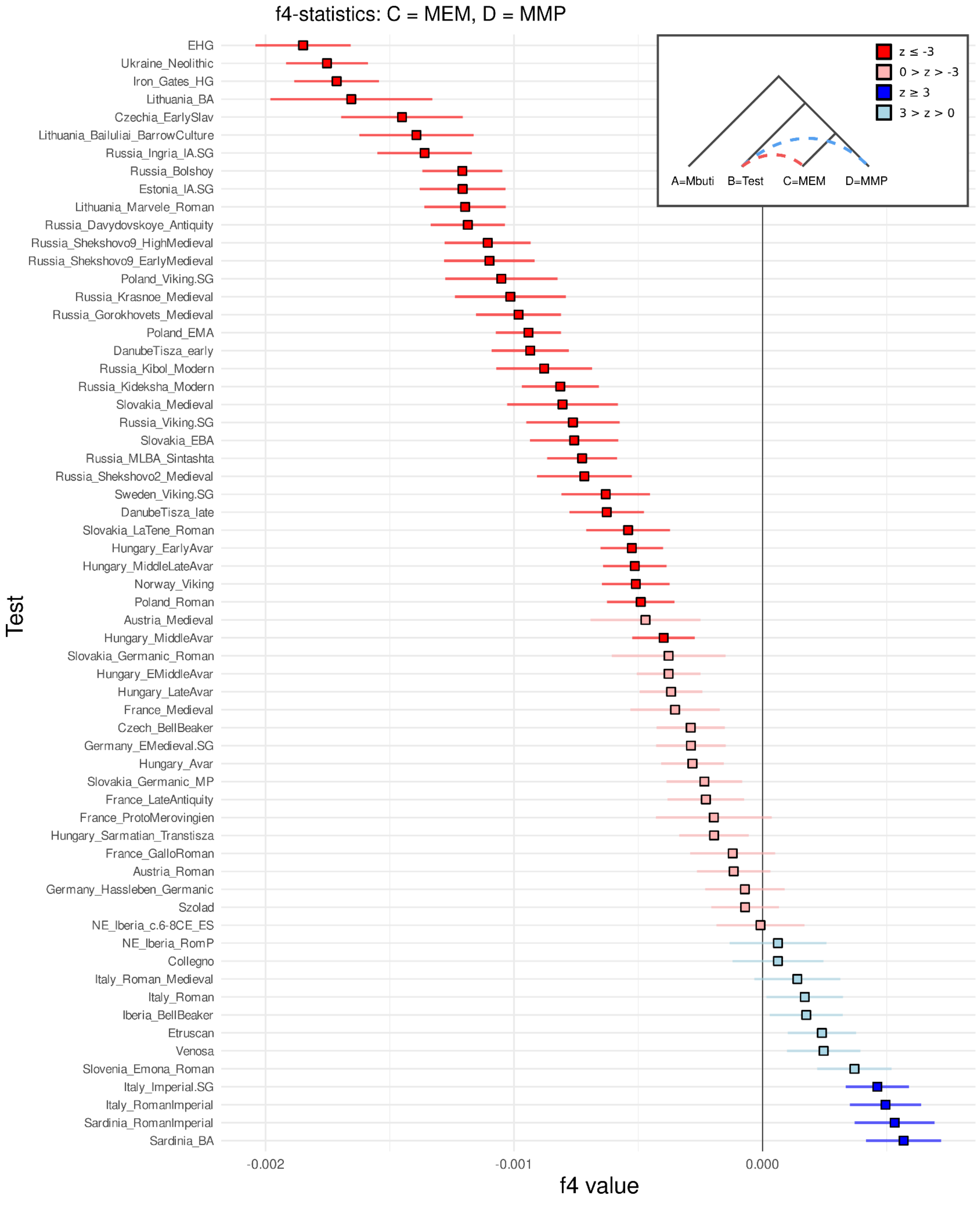

Fig. S19: f4-statistics in the form of *f4(Outgroup,test;MEM,MMP)*).

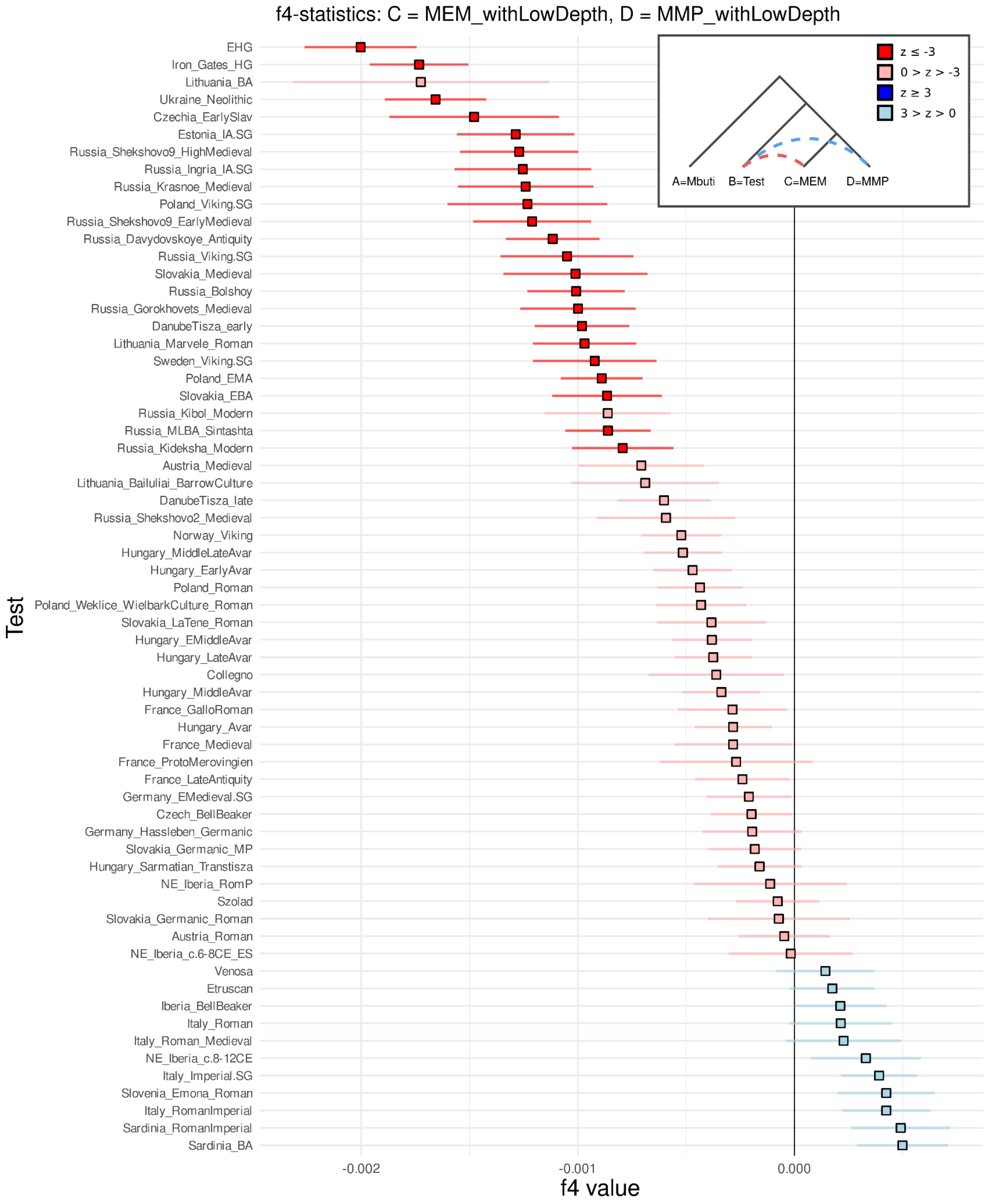

Fig. S20: f4-statistics in the form of *f4(Outgroup,test;MEM,MMP)*) including the low-depth sequenced samples.

3.5 Uniparentally inherited markers analysis

Y-chromosome haplogroups were inferred with the *Y-Lineage-Tracker*'s subcommand "*classify*" (72) using Y-chromosome genotype calls produced taking into consideration sequencing biases and degradation from PMD.
This was done by estimating PMD patterns with *ATLAS*'s PMD and sequencing biases with the tool's PMD estimator as described in SI 4.2.5.2.
Genotypes were estimated like in chapter 5 for each individual separately, with the difference that *ATLAS*'s call tool was run with the "*allelePresence*" call instead of "*MLE*" *(task=call method=allelePresence*).
Y-Lineage-Tracker's "*classify*" was then run using the Y-chromosome genotype calls as input, using the *ISOGG* Y-haplogroup tree v.15.73 (https://isogg.org/tree/) as reference and default parameters. The "Key Haplogroup" from the program's output is reported in Additional file 4: Dataset S7, together with the leaf classification reporting the most derivative mutation that is phylogenetically relevant.

Mitochondrial DNA haplogroups were estimated with *HaploGrep* v2.51 (73) using the individual consensus mitochondrial genome produced by Schmutzi (74). Haplogroups with less than 0.8 quality from the *HaploGrep*'s output were excluded from the plot but included together with the high quality haplogroups in Additional file 4: Dataset S7.

Figures summarizing the analyses of uniparentally inherited markers results were plotted with "*treemap*" version 2.4-3 and manually adapted with *Inkscape* v1.3.2 (https://inkscape.org), and are presented in Fig. S21. Colours for the treemap graphs were randomly sampled from the "*viridis*" package's colours palette version 0.6.4.

The uniparentally inherited markers analysis yielded mainly deep diverging haplogroups for both mtDNA and Y-chromosome, and both for samples from the MMP samples and the MEM samples. There are only two exceptions, with two couples of MEM individuals sharing the same Y chromosomal haplogroups (I2a-A815 and R1a-CTS3402).

All the haplogroups described have their highest frequencies in the Western Eurasia both in the first millennium CE (7, 75–78) and nowadays (79–81).

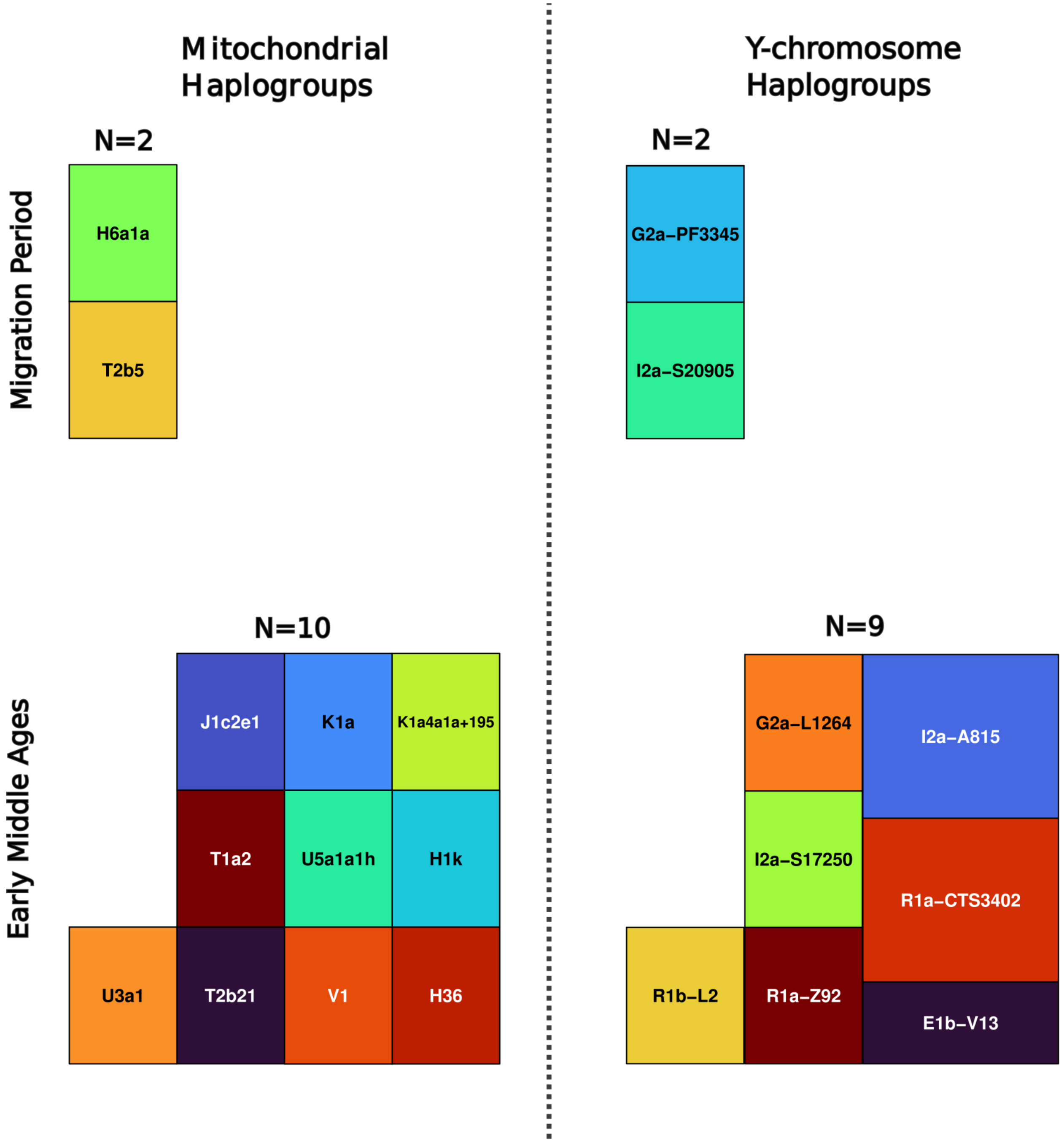

Fig. S21: Summarization of the inferred Y-chromosome and mitochondrial haplogroups

3.6 MOSAIC

MOSAIC v.1.3.7 was used to infer admixture events and dates in ancient samples, using an expanded set of 610 individuals from 48 present-day European and Asian populations from the HellBus dataset as surrogates for the admixing sources. Ancient populations were modeled as either a 2 or 3 way mixture of 48 present-day populations and the non-target ancient population. Upper and lower quantiles for admixture dates were estimated from a bootstrap procedure implemented using the *bootstrap_chromosomes_coanc_curves* function from the *MOSAIC* API. Other than changing the number of mixing sources, all other parameters were left as default. The results are in Fig. S22 and S23. Based on *MOSAIC* results of present day populations modeling, a model of mixture between sources close to the MMP, MEM (including only whole genome samples) and East-Asians seems plausible (Fig. S23).

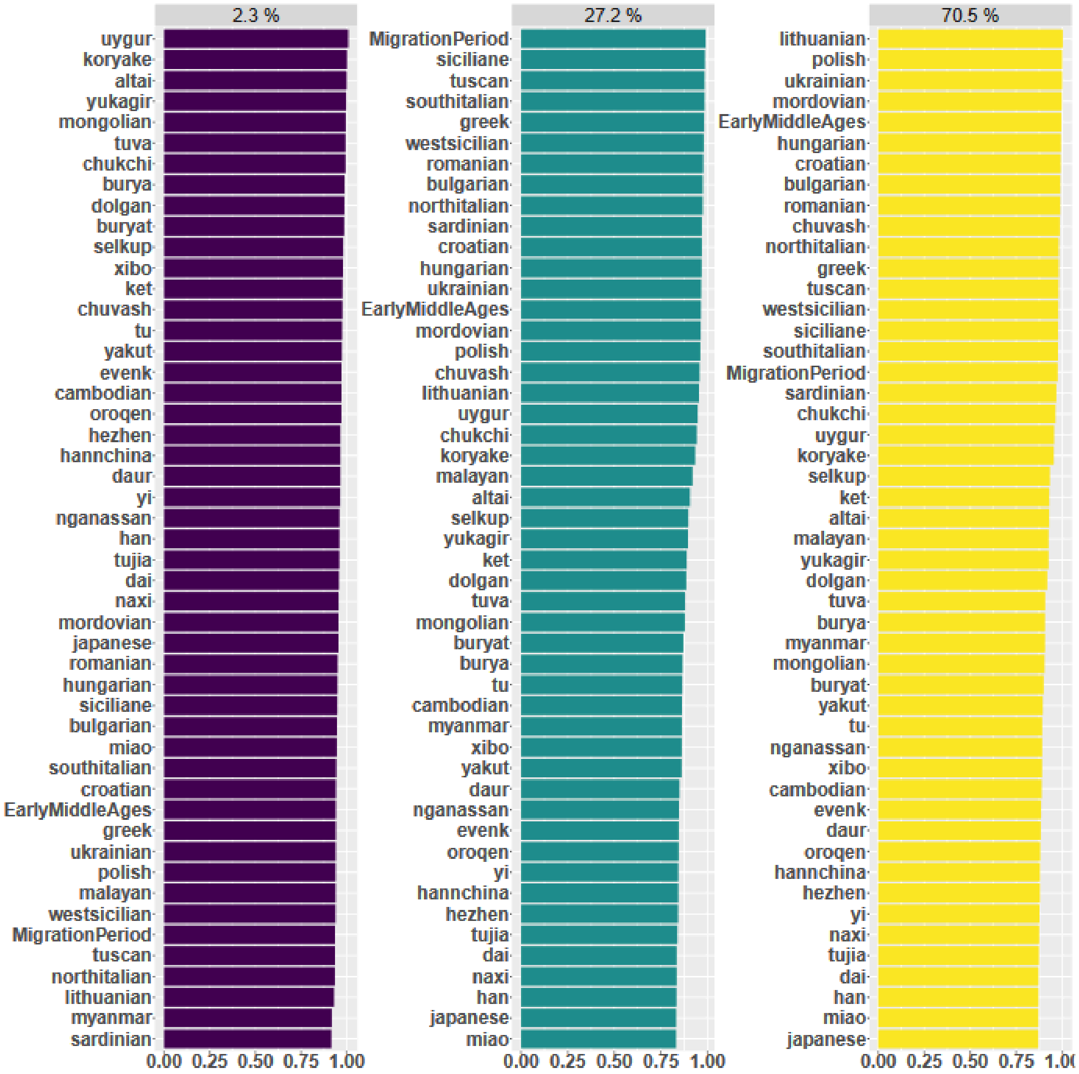

Fig. S22: 1-Fst between 3 inferred mixing sources for present-day Belarussians. Each panel represents a different mixing source. Each bar gives the value 1-Fst between sample population and the mixing source. Higher values of 1-Fst suggest that source is well represented by a particular population.

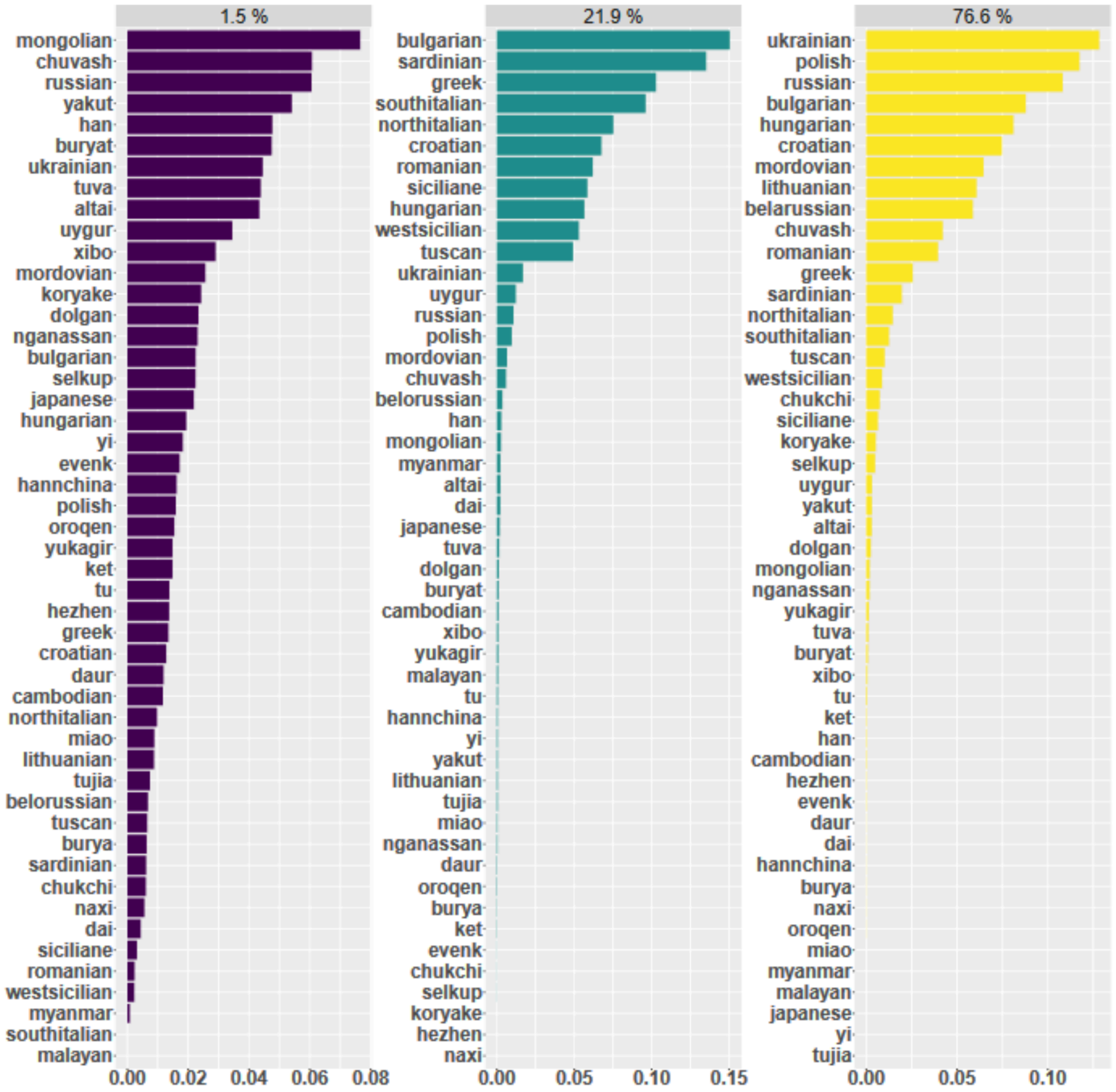

Fig. S23: Copying matrix plot for sources in three-way admixture event for MEM ancient samples. Each panel represents one of the three putative mixing sources. Labels above each panel give the proportion that mixing source contributed to the MEM samples. Length of the bars within each panel reflects how to best represent the relative haplotype composition of that source using the surrogate populations.

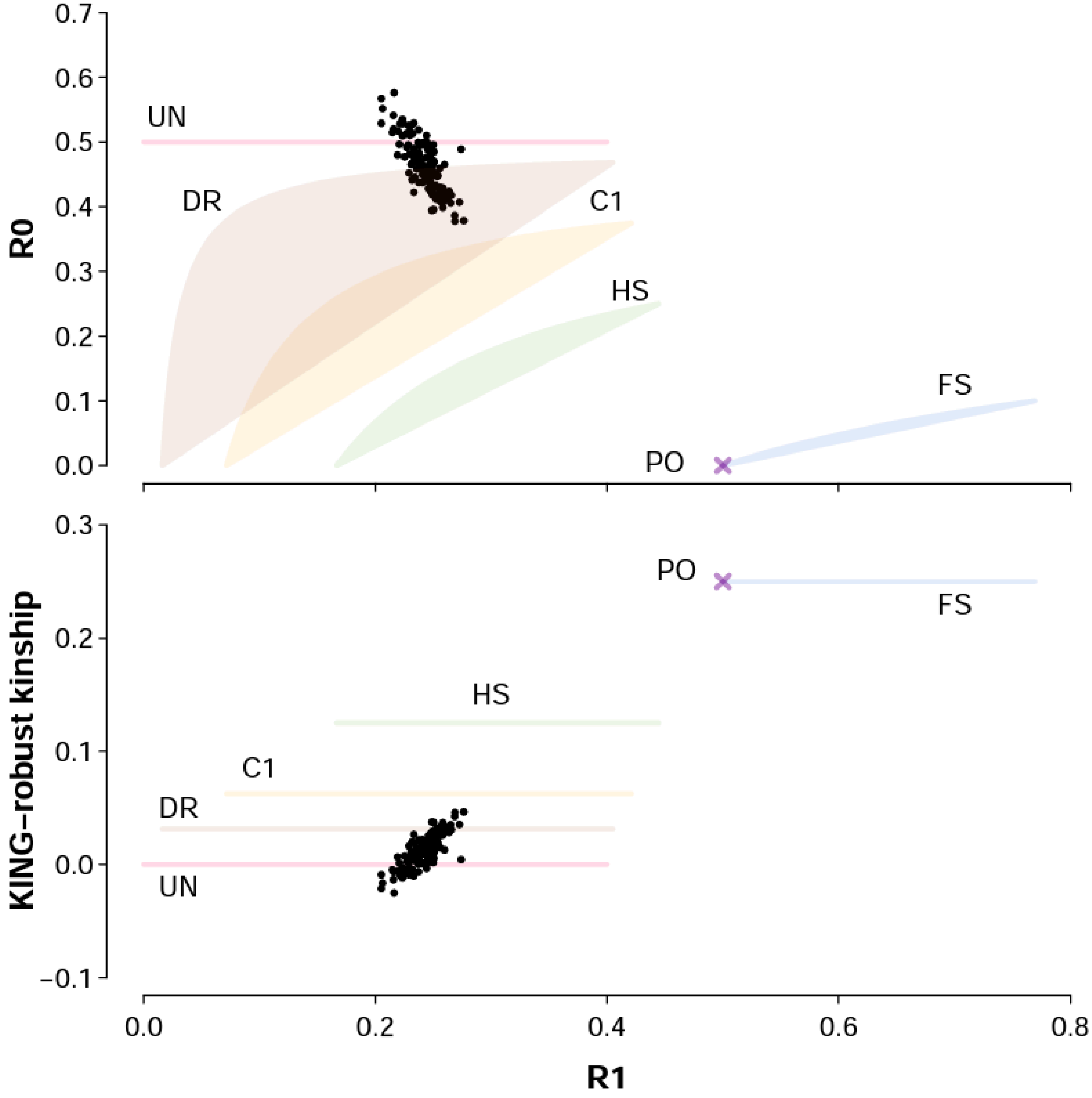

Fig. S24: Biological relatedness. No relatedness closer than distant relatives was found among the samples from Pohansko and Libiva presented in this study.

.

4 Data availability

The newly produced aligned sequence data are deposited in the European Nucleotide Archive (ENA) under the accession number PRJEB39997.

5 Legends for Dataset S1 to S8

- Dataset S1: Metadata for all processed samples.
  - Sheet 1: Legend for all sheets.
  - Sheet 2: Summary statistics per analysed sample initial low-depth shotgun screening.
  - Sheet 3: Summary statistics per analysed sample - deeper shotgun sequencing.
  - Sheet 4: Summary statistics per fastq file.
  - Sheet 5: Summary statistics for PJP010.
  - Sheet 6: Contamination estimation of the WG samples.
- Dataset S2: Outgroup-f3 statistic
- Dataset S3: f4 statistic
- Dataset S4: qpWave
- Dataset S5: qpAdm
- Dataset S6: Testing qpAdm plausible models
- Dataset S7: Uniparentally inherited markers analysis
- Dataset S8: Target set for local realignment

6 SI References

1. P. Heather, Why Did the Barbarian Cross the Rhine? *J. Late Antiq.* **2**, 3–29 (2009).

2. V. Blažek, CLASSIFICATION OF SLAVIC LANGUAGES: EVOLUTION OF DEVELOPMENTAL MODELS. *Slavia Occident.* 33–64 (2020). https://doi.org/10.14746/so.2020.77.3.

3. F. Curta, *Slavs in the making: history, linguistics and archaeology in Eastern Europe (ca. 500-ca. 700)* (Routledge, Taylor & Francis Group, 2021).

4. R. Nedoma, “Schrift und Sprache in den südgermanischen Runeninschriften” in *Das Fuþark Und Seine Einzelsprachlichen Weiterentwicklungen*, A. Bammesberger, G. Waxenberger, Eds. (DE GRUYTER, 2006), pp. 109–156.

5. W. Menghin, *Die Langobarden: Archäologie und Geschichte* (K. Theiss, 1985).

6. W. Pohl, P. Erhart, *Die Langobarden: Herrschaft und Identität* (Verl. der Österr. Akad. der Wiss, 2005).

7. C. E. G. Amorim, *et al.*, Understanding 6th-century barbarian social organization and migration through paleogenomics. *Nat. Commun.* **9**, 3547 (2018).

8. H. Birnbaum, On the Ethnogenesis and Protohome of the Slavs: The Linguistic Evidence. *J. Slav. Linguist.* **1**, 352–374 (1993).

9. T. Klír, V. Boček, N. Jansens, *New perspectives on the Early Slavs and the rise of Slavic: contact and migrations* (Universitätsverlag Winter, 2020).

10. M. Mielnik-Sikorska, *et al.*, The History of Slavs Inferred from Complete Mitochondrial Genome Sequences. *PLoS ONE* **8**, e54360 (2013).

11. A. Juras, *et al.*, Ancient DNA Reveals Matrilineal Continuity in Present-Day Poland over the Last Two Millennia. *PLoS ONE* **9**, e110839 (2014).

12. M. Parczewski, Origins of Early Slav Culture in Poland. *Antiquity* **65**, 676–683 (1991).

13. F. Curta, *The making of the Slavs: history and archaeology of the Lower Danube Region, ca. 500-700* (Cambridge University Press, 2001).

14. F. Curta, The early Slavs in Bohemia and Moravia: a response to my critics. *Archeol. Rozhl.* **61**, 725–745 (2009).

15. D. Černín, Faking a Collision Course: When History Clashes with Populism. *Filozofia* **77**, 97–111 (2022).

16. M. Gojda, *The ancient Slavs: settlement and society* (Edinburgh University Press, 1991).

17. P. M. Barford, *The early Slavs: culture and society in early medieval Eastern Europe* (Cornell University Press, 2001).

18. M. Parczewski, “Slavs and the early slav culture” in *Ancient Europe 8000 B.C.--A.D. 1000: Encyclopedia of the Barbarian World*, P. I. Bogucki, P. J. Crabtree, Eds. (Thompson/Gale, 2004).

19. J. Schneeweiß, *Zwischen den Welten. Archäologie einer europäischen Grenzregion zwischen Sachsen, Slawen, Franken und Wikingern*, Seminar für Ur- und Frühgeschichte der

Georg-August-Universität Göttingen, Ed. (Wachholtz Verlag, 2020).

20. S. Eichert, J. Macháček, N. Brundke, “Frontier – Contact Zone – No Man’s Land : The March/Morava – Thaya/Dyje. Borderregion during the Early Middle Ages” in *Power in Landscape : Geographic and Digital Approaches on Historical Research*, S. Popovic Mihailo St; Polloczek, Veronika; Koschicek, Bernhard; Eichert, Ed. (Eudora-Verlag, 2019), pp. 45–63.

21. F. Curta, Pots, Slavs and ‘imagined communities’: Slavic archaeologies and the history of the early Slavs. *Eur. J. Archaeol.* **4**, 367–384 (2001).

22. J. Macháček, *et al.*, Runes from Lány (Czech Republic) - The oldest inscription among Slavs. A new standard for multidisciplinary analysis of runic bones. *J. Archaeol. Sci.* **127**, 105333 (2021).

23. M. Kara, Archaeology, mainly polish, in the current discussion on the ethnogenesis of the Slavs. *Slavia Antiq. Rocz. Poświęcony Staroż. Słow.* 65–128 (2022). https://doi.org/10.14746/sa.2022.63.3.

24. M. Furholt, Re-integrating Archaeology: A Contribution to aDNA Studies and the Migration Discourse on the 3rd Millennium BC in Europe. *Proc. Prehist. Soc.* **85**, 115–129 (2019).

25. A. Pleterski, *The invisible Slavs: Župa Bled in the prehistoric Early Middle Ages* (Inštitut za Arheologijo ZRC SAZU, Založba ZRC, 2013).

26. S. Brather, The Western Slavs of the Seventh to the Eleventh Century - An Archaeological Perspective: Western Slavs of the Seventh to the Eleventh Century. *Hist. Compass* **9**, 454–473 (2011).

27. P. J. Heather, *Empires and barbarians: the fall of Rome and the birth of Europe* (Oxford University Press, 2010).

28. B. Dostál, *Břeclav-Pohansko. IV, Velkomoravský velmožský dvorec* (Universita J.E. Purkyně, 1975).

29. B. Dostál, *K prehistorii a protohistorii Břeclavi* (Musejní Spolek, 1968).

30. E. Klanicová, *Archeologické památky na katastrálním území Břeclavi* (Město Břeclav, 2001).

31. P. Dresler, J. Macháček, The history of settlement and the cultural landscape in the lower Dyje (Thaya) River region in the Early Middle Ages. *Archeol. Rozhl.* **65**, 663–705 (2013).

32. J. Macháček, *The rise of medieval towns and states in East Central Europe: early medieval centres as social and economic systems* (Brill, 2010).

33. F. Kalousek, *Die grossmährische Burgwallstadt Břeclav-Pohansko* (Sborník prací Filozofické fakulty brněnské univerzity. E, Řada archeologicko-klasická, 1960).

34. B. Dostál, *Drobná pohřebiště a rozptýlené hroby z Břeclavi-Pohanska* (Sborník prací filozofické fakulty brněnské univerzity, 1982).

35. J. Vignatiová, *Břeclav-Pohansko* (Masarykova univerzita, 1992).

36. E. Drozdová, *Břeclav-Pohansko: demografická a antropometrická studie*, vyd. 1 (Masarykova univerzita, 2005).

37. J. Macháček, *Kostelní pohřebiště na Severovýchodním předhradí* (Verlag nicht ermittelbar, 2017).

38. R. Přichystalová, K. Kalová, K. Boberová, J. Nováček, *Pohřební areály z Jižního předhradí: archeologicko-antropologická studie* (Masarykova univerzita, 2019).

39. J. Macháček, A. Balcárková, P. Dresler, M. Prišťáková, *Sídelní areál na Severovýchodním předhradí: archeologické výzkumy v letech 2006-2016* (Masarykova univerzita Brno, 2021).

40. F. Kalousek, *Břeclav-Pohansko. I, Velkomoravské pohřebiště u kostela : archeologické prameny z pohřebiště* (Universita J.E. Purkyně, 1971).

41. J. Macháček, Raně středověké Pohansko u Břeclavi: munitio, palatium, nebo emporium moravských panovníků? *Archeol. Rozhl.* **LVII** (2005).

42. J. Macháček, "Zpráva o archeologickém výzkumu Břeclav-Líbivá 1995-1998" in *Archaeologia Mediaevalis Moravica et Silesiana I*, Masarykova univerzita v Brně, Ed. (2015).

43. J. Macháček, E. Klanicová, in *Neue Beiträge zur Erforschung der Spätantike im mittleren Donauraum: Materialien der Internationalen Fachkonferenz "Neue Beiträge zur Erforschung der Spätantike im mittleren Donauraum", Kravsko 17.-20. Mai 1995*, Spisy Archeologického Ústavu AV ČR Brno., J. Tejral, Archeologický ústav, Eds. (Archäologisches Inst. der Akad. der Wiss, 1997).

44. J. Tejral, "Archäologisch-kulturelle Entwicklung im norddanubischen Raum am Ende der Spätkaiserzeit und am Anfang der Völkerwanderungszeit" in *L'Occident romain et l'Europe centrale au début de l'époque des Grandes Migrations*, Spisy Archeologického Ústavu AV ČR v Brně., J. Tejral, C. Pilet, M. Kazanski, Basse-Normandie, Eds. (Archeologický Ústav Akademie Věd České Republiky Brno, 1999).

45. N. Profantová, A. Šilhová, Early Medieval "kaptorgas" in Bohemia. Analysis of the find from grave no. 22 at the Klecany II burial grounds. *PAMATKY Archeol.* **101**, 283–310 (2010).

46. J. Macháček, S. Eichert, V. Nosek, E. Pernicka, Copper-alloy belt fittings and elite networking in Early Medieval Central Europe. *J. Archaeol. Sci.* **161**, 105895 (2024).

47. P. Dresler, J. Macháček, R. Přichystalová, in *Burg--Vorburg--Suburbium: zur Problematik der Nebenareale frühmittelalterlicher Zentren*, Spisy archeologického ústavu AV ČR Brno., I. Boháčová, L. Poláček, Archeologický ústav Akademie věd České republiky v Brně, Eds. (Archäologisches Institut der Akademie der Wissenschaften der Tschechischen Republik, 2008).

48. R. Přichystalová, "Nerituálny pohreb muža z južného predhradia na Pohansku pri Břeclavi" in *Zaměřeno Na Středověk. Sborník k 60tým Narozeninám Prof. Zdeňka Měřínského*, (Nakladatelství Lidové noviny, 2010).

49. L. Horáčková, L. Vargová, J. Macháček, Antropologicko-lékařský výzkum kosterních pozůstatků z doby stěhování národů (Líbivá u Břeclavi) (Předběžné sdělení) in *Archaeologia mediaevalis Moravica et Silesiana I*, (Masarykova univerzita v Brně, 1999), pp. 63–69.

50. J. Košta, J. Hošek, P. Dresler, J. Macháček, R. Přichystalová, Velkomoravské meče z Pohanska u Břeclavi a okolí-nová revize. *Památky Archeol.* **110** (2019).

51. D. Jelínková, "K chronologii sídlištních nález\uu s keramikou pražského typu na MoravěK chronologii sídlištních nálezů s keramikou pražského typu na Moravě – Zur Chronologie der Siedlungsbefunde mit Keramik des Prager Types in Mähren" in *Pravěké a Slovanské Osídlení Moravy. Sborník k*, V. Nekuda, J. Unger, M. Čižmář, Eds. (1990), pp. 251–281.

52. M. Kuna, *et al.*, Počátky raného středověku v Čechách/The Onset of the Early Middle Ages in Bohemia. (2005).

53. G. Fusek, *Slovensko vo včasnoslovanskom období* (Archeologický Ústav Slovenskij Akadémie Vied, 1994).

54. C. B. Ramsey, Deposition models for chronological records. *Quat. Sci. Rev.* **27**, 42–60 (2008).

55. C. Bronk Ramsey, OxCal v4. 4.4. *Available Retrieved Httpsc14 Arch Ox Ac Ukoxcal Html* (2021).

56. T. R. McLaughlin, On Applications of Space–Time Modelling with Open-Source 14C Age Calibration. *J. Archaeol. Method Theory* **26**, 479–501 (2019).

57. J. Tejral, "Suebi north of the Middle Danube" in *In tempore sueborum: el tiempo de los suevos en la Gallaecia (411-585): el primer reino medieval de Occidente*, J. López Quiroga, A. M. Martínez Tejera, Eds. (Deputación Provincial de Ourense, 2017).

58. H. Li, R. Durbin, Fast and accurate short read alignment with Burrows–Wheeler transform. *Bioinformatics* **25**, 1754–1760 (2009).

59. P. Danecek, *et al.*, Twelve years of SAMtools and BCFtools. *GigaScience* **10**, giab008 (2021).

60. G. A. Van der Auwera, B. D. O'Connor, *Genomics in the cloud: using Docker, GATK, and WDL in Terra* (O'Reilly Media, 2020).

61. Q. Fu, *et al.*, A Revised Timescale for Human Evolution Based on Ancient Mitochondrial Genomes. *Curr. Biol.* **23**, 553–559 (2013).

62. T. S. Korneliussen, A. Albrechtsen, R. Nielsen, ANGSD: Analysis of Next Generation Sequencing Data. *BMC Bioinformatics* **15**, 356 (2014).

63. S. Rubinacci, D. M. Ribeiro, R. J. Hofmeister, O. Delaneau, Efficient phasing and imputation of low-coverage sequencing data using large reference panels. *Nat. Genet.* **53**, 120–126 (2021).

64. D. J. Lawson, G. Hellenthal, S. Myers, D. Falush, Inference of Population Structure using Dense Haplotype Data. *PLoS Genet.* **8**, e1002453 (2012).

65. M. Salter-Townshend, S. Myers, Fine-Scale Inference of Ancestry Segments Without Prior Knowledge of Admixing Groups. *Genetics* **212**, 869–889 (2019).

66. M. Caduff, R. Eckel, C. Leuenberger, D. Wegmann, "Accurate Bayesian inference of sex chromosome karyotypes and sex-linked scaffolds from low-depth sequencing data" (Genomics, 2023).

67. M. Petr, B. Vernot, J. Kelso, admixr—R package for reproducible analyses using ADMIXTOOLS. *Bioinformatics* **35**, 3194–3195 (2019).

68. R Core Team, *R: A Language and Environment for Statistical Computing* (R Foundation for Statistical Computing, 2021).

69. N. Patterson, *et al.*, Ancient Admixture in Human History. *Genetics* **192**, 1065–1093 (2012).

70. D. Reich, *et al.*, Reconstructing Native American population history. *Nature* **488**, 370–374 (2012).

71. W. Haak, *et al.*, Massive migration from the steppe was a source for Indo-European languages in Europe. *Nature* **522**, 207–211 (2015).

72. H. Chen, Y. Lu, D. Lu, S. Xu, Y-LineageTracker: a high-throughput analysis framework for

Y-chromosomal next-generation sequencing data. *BMC Bioinformatics* **22**, 114 (2021).

73. H. Weissensteiner, *et al.*, HaploGrep 2: mitochondrial haplogroup classification in the era of high-throughput sequencing. *Nucleic Acids Res.* **44**, W58–W63 (2016).

74. G. Renaud, V. Slon, A. T. Duggan, J. Kelso, Schmutzi: estimation of contamination and endogenous mitochondrial consensus calling for ancient DNA. *Genome Biol.* **16**, 224 (2015).

75. M. L. Antonio, *et al.*, Ancient Rome: A genetic crossroads of Europe and the Mediterranean. *Science* **366**, 708–714 (2019).

76. J. Gretzinger, *et al.*, The Anglo-Saxon migration and the formation of the early English gene pool. *Nature* **610**, 112–119 (2022).

77. A. Margaryan, *et al.*, Population genomics of the Viking world. *Nature* **585**, 390–396 (2020).

78. I. Olalde, *et al.*, A genetic history of the Balkans from Roman frontier to Slavic migrations. *Cell* **186**, 5472-5485.e9 (2023).

79. A. Kushniarevich, *et al.*, Genetic Heritage of the Balto-Slavic Speaking Populations: A Synthesis of Autosomal, Mitochondrial and Y-Chromosomal Data. *PLOS ONE* **10**, e0135820 (2015).

80. S. Rootsi, *et al.*, Phylogeography of Y-Chromosome Haplogroup I Reveals Distinct Domains of Prehistoric Gene Flow in Europe. *Am. J. Hum. Genet.* **75**, 128–137 (2004).

81. O. Semino, *et al.*, The Genetic Legacy of Paleolithic *Homo sapiens sapiens* in Extant Europeans: A Y Chromosome Perspective. *Science* **290**, 1155–1159 (2000).
