# Supplementary material for: Ancient genomes provide evidence of demographic shift to Slavic-associated groups in Moravia
Source: Genome Biol. 2025 Sep 3;26:259. doi: 10.1186/s13059-025-03700-9 (PMC12409924; doi:10.1186/s13059-025-03700-9)
Supplement: Supplementary file 6 — Additional File 6. Peer review history. [file 13059_2025_3700_MOESM6_ESM.docx]

Review History

**First round of review**

**Reviewer 1**

**Were you able to assess all statistics in the manuscript, including the appropriateness of statistical tests used?**

Yes, and I have assessed the statistics in my report.

**Comments to author:**

In the paper "Ancient genomes from Moravia evidence demographic spread of Slavic-associated groups", Schultz et al analysed high-quality genomic data of 18 individuals from South Moravia, from a crucial region and time frame to shed light and to understand the genetic features of the Slavic expansions.
The samples are very interesting and their high quality allows to perform interesting analyses, that the Authors indeed performed, but not always adequately discussed. However, the way the manuscript is presented now makes it difficult to understand the work and its main results and in some cases the Authors' conclusions seem not to be supported by the data (or maybe the link between the data and the conclusions is not sufficiently highlighted). In addition, often the relevant supplementary data are not cited in the main text, so it is really hard to check the discussed results.
Overall, the manuscript seems to lack coherent organisation and it is hard to follow the logical steps from data analysis to results to discussion.
For these reasons, I believe that this paper can be suitable for publication after major revisions under both the presentation and the interpretation point of view.

MAJOR POINTS
1) The Authors state that they analysed 54 individuals, but then increased the coverage for 17 of them. However, they do not report the sequencing information for all the 54 individuals: this is not acceptable, both in general in the aDNA field and especially since the Authors claim that the samples to analyse at higher coverage have been selected among those with an endogenous DNA content > 20%. How can this be checked if no details are reported?
2) In addition to the previous point, the Authors should give some more information about their choices regarding the samples to analyse at higher coverage. In the text, they say that the 17 samples represent a cross-section of the social structure but no sample-by-sample information about this is provided (or can be easily found). Adding this to Dataset S5 would be useful.
Moreover, dataset S5 includes three sheets: what is the difference between sheet 1 and 2? Some more explanations as table caption should be added (this is true for all the tables and datasets). And why are some published samples present too? I could not find any mention to them in the main text and there is just a brief mention in the supplementary.
3) The supplementary information includes a huge amount of data, some necessary to understand the results reported in the main text, some others no mentioned at all in the main text (e.g. all the section about uniparental markers). Since the journal does not have a strict word count, I suggest to the Authors to move some data in the main text to let the readers find the relevant information in a faster/easier way, especially regarding the methods.
Moreover, if the data in supplementary are not mentioned at all in the main text, maybe the Authors should decide if they are really relevant (and then some words in the main text should be added - e.g. can the Y chromosome haplogroups give any additional input regarding the Eastern Asian ancestry reported in lines 18-37, page 8?) or if they should be deleted. Finally, some plots seem to be duplicated between the main text and the supplementary file (e.g. Suppl Fig 22 and Fig 2B but other instances may be present): this must be avoided and fixed.
4) The Authors' choice to relegate the "standard" projected PCA in the supplementary is curious: what is the reason? The "standard" PCA is something quite common in the field that can help the readers to visualise the data in a known context: maybe the authors can consider including both PCAs (projected and haplotype-based) as main figures.
5) The low coverage samples can indeed be used for some analyses, including the projected PCA (in some papers, a coverage as low as 0.04x or even 0.02x is considered sufficient): since the Authors did this analysis, why don't they include as many samples as possible? Although low-coverage samples cannot be used for haplotype-based analyses, including them in the frequency-based analyses can help to elucidate the general picture. For example, based on 7 samples the Authors claim that the MMP samples are really diverse along a cline. This is reasonable according to the literature, but how can you state it from only 7 samples? Making an extreme example, it is possible that you only collected outliers by chance. The positions of the other samples in the PCA can give you additional support. The same is true for f3, f4, qpWave/qpAdm, etc.
6) In the Discussion, the Authors often support the hypothesis of a complete population replacement in the region but this is not supported by their own data. In the first paragraph of the discussion, the Authors claim: "Conservative estimates given existing reference populations, however, suggest that the contribution of the immigrating population was dominant or even completely replaced the local population in this region" (lines 54-58, page 8). Not only there are no results supporting this, but the data support a contribution of MMP to MEM (as the Authors themselves state a few lines before, lines 4-5, same page), thus clearly rejecting the hypothesis of a complete replacement. Similarly, in lines 32-54 page 9, they state that the Slavic became more heterogeneous over time, but their data show more ancestry diversity in previous periods (MMP) rather than in later ones (MEM). All the Discussion needs to be rewritten to take into account what the results show (or this section needs to be better explained, if my interpretation is not what the Authors meant).

MINOR POINTS
Introduction
Lines 41-48, page 5: It would be useful to cite Fig 1 here already.
Lines 46-52, page 4: This sentence seems incomplete (from "indigenous").
Lines 19-20, page 5: A dot is missing after Balkans.

Results
Line 19, page 6: What do the authors mean with "shallow whole genome sequencing"? A different term should be used instead of "shallow".
Line 22, page 6: Dataset S5 is mentioned, but no mention to Datasets S1-4 has been made before: the supplementary should be named in the order they appear in the text (and all the supplementary should be cited, but for example I could not find any mention to dataset S1).
Line 11, page 7: "albeit noiser due to lower power", what do the Authors mean?
Line 23-24, page 7: the expression "direct continuity" is misleading here, because MEM seem to descend from MMP + additional sources (and so some sort of "direct" continuity can be indeed observed). The Authors should rephrase this sentence.
Lines 26-41, page 7: Basing the differentiation between MMP and MEM on the Relate data seems to be an overinterpretation of this result. The difference date back to 6000 BCE (so, approximately 7 millennia before the period under study) and, as the Authors themselves state, the higher Ne of MMP can result from individuals of different ancestries. I wonder if including more samples (i.e. using tools - such as ASCEND https://github.com/sunyatin/ASCEND - that can deal with low coverage aDNA data and including the other MMP and MEM individuals) can give different results. If so, I suggest removing this part from the main text (especially the figure 3) and/or tuning it down.
Lines 43-56, page 7: I cannot understand the logic behind this. Why should they form a clade if they are so diverse including individuals from different ancestries? Is it not exactly what we would expect? Maybe f3 and f4 statistics would be a better tool to investigate the relationship between Longobard, MMP and MEM, both by groups or specific individuals. Also a comparison with Avars can be informative?
Line 59: again, the expression "direct descendants" should be rephrased.
Lines 31-32, page 8: a closing bracket is missing.
Lines 11-13, page 8: since the Authors performed chromopainter/fineSTRUCTURE, can the MOSAIC results be confirmed by GLOBETROTTER/SOURCEFIND?

Figures
Figure 1, panel B: I am not an expert of the Moravia historical periods. Is the chronological order: Migration period, Prague culture, Early Hillfort and Middle Hillfort? Considering the importance of this for the data interpretation, maybe it would be helpful to add also this information to the timeline in this panel (in addition to the useful Migration period/Early Middle Ages labels already present).
Figure 2, panel C: its utility and meaning is not clear, I suggest to remove it; a sentence in the text is sufficient.
Supplementary Data
Supplementary figures 11-13: the images are at very low resolution and the colours are hardly distinguishable.
Supplementary figure 14: the symbols for LIB7, LIB11 and MMP seems to have been switched in the legend.

**Reviewer 2**

**Were you able to assess all statistics in the manuscript, including the appropriateness of statistical tests used?** Yes, and I have assessed the statistics in my report.

**Comments to author:**

This manuscript provides an important contribution to the debate regarding the demographic history of Slavic-associated populations in Central Europe, presenting genomic data from two burial sites in South Moravia. While the study introduces new data to an underexplored period, there are several concerns regarding the interpretation of results, particularly in terms of the proposed genetic turnover and population movement.

Key Points of Concern:

The conclusions regarding a major genetic turnover between the Migration Period and Early Medieval populations are drawn from a relatively small sample size of 18 individuals. While these samples are of high quality, the limited number of individuals, all from two sites, restricts the broader applicability of the findings. Given the small sample size, it is difficult to generalize these results to broader population dynamics in Central Europe, let alone infer a large-scale genetic turnover. A more extensive dataset from a wider range of sites would provide a stronger basis for the conclusions drawn.

The study presents a substantial temporal gap, particularly in the 6th century, a period critical for understanding the early phases of Slavic expansion. Most of the individuals in the Early Medieval sample date to after the 7th century, while the Migration Period samples date to the 5th century. Without sufficient representation from the 6th century, it becomes difficult to determine whether the genetic changes occurred gradually or as a result of abrupt events such as migration. This lack of temporal continuity limits the study's ability to trace the exact nature of population shifts, and additional sampling from this gap would enhance the analysis.

One significant issue is the broad temporal range (7th to 10th century CE) over which the Early Medieval samples are spread. Despite this, the study treats the Early Medieval individuals as a largely homogeneous group, which may oversimplify the genetic and social changes that occurred over this 300-year period. These centuries were marked by significant political and cultural shifts, and pooling the Early Medieval samples together risks overlooking important intra-period variation. Differentiating between the earlier and later Early Medieval samples could offer a more nuanced understanding of genetic continuity and change over time, avoiding overly broad conclusions about population stability or replacement.

The observed genetic differentiation between the Migration Period and Early Medieval individuals is interpreted as evidence of a major population movement. However, this interpretation might be somewhat overextended. Genetic shifts can result from a variety of factors, including internal social dynamics, marriage patterns, or selective gene flow, rather than outright population replacement. While migration is one possible explanation, the dataset does not provide unequivocal evidence for this scenario, and alternative processes should be considered more carefully. A more cautious interpretation of the data, acknowledging other potential drivers of genetic change, would strengthen the study.

The connection drawn between genetic findings and the spread of Slavic-associated groups remains speculative. The study suggests that the genetic changes observed could be linked to the arrival of Slavic-speaking populations, but the evidence for this claim is indirect. Material culture, such as Prague-type pottery, does not necessarily imply linguistic or ethnic continuity. Without concrete historical or linguistic data directly tying the individuals in the study to Slavic identities, the conclusion that this represents the spread of Slavic-speaking groups should be considered carefully and framed as a hypothesis rather than a definitive finding.

The study notes the challenge posed by the lack of reference populations from North-Eastern Europe, which limits the ability to precisely contextualize the findings. The absence of comparable ancient DNA data from nearby regions makes it difficult to determine whether the genetic shifts observed in South Moravia were part of a broader regional trend or a localized event. Incorporating more comparative datasets from surrounding regions and time periods would provide a clearer understanding of how the genetic changes in South Moravia fit within the broader population history of Europe.

This manuscript provides valuable insights into the genetic history of early medieval populations in South Moravia, contributing new data to the study of Slavic-associated groups. However, the small sample size, significant temporal gaps, and lack of differentiation among Early Medieval samples complicate the interpretation of genetic turnover. The conclusions regarding large-scale migration and population replacement should be approached with caution, given the multiple potential drivers of genetic shifts. More robust sampling and the inclusion of additional comparative data would help clarify the nature of population dynamics in this region. Further research is needed to fully understand the genetic and cultural transformations of early medieval Central Europe.

**Reviewer 3**

**Were you able to assess all statistics in the manuscript, including the appropriateness of statistical tests used?** Yes, and I have assessed the statistics in my report.

**Comments to author:**

I would thank authors for putting together a nice, structured and concise manuscript. I enjoyed reading it!

I have a few small comments and one concern. I deeply hope that they might help to strengthen the conclusion authors make.

Title:

I suggest editing the title to specify the region the authors are referring to when they mention 'demographic spread.' In its current form, it could be interpreted as applying to the entire Slavic area. While this might be true for the studied microregion in Central Europe, there is not yet sufficient data to generalize this to a wider area.

Introduction:

Line 9 (However, archaeological evidence …): This statement is questionable because there is no consensus among archaeological schools about which archaeological culture should be associated with the Slavic tribes. Additionally, the Prague culture is not homogenous.

Results:

Line 2 (North-Western Slavs such as ..): Lithuanian are Baltic-speaking population, not Slavs.

PCA projection and PCA on likelihoods: could authors provide their motivation in using these two approaches to confirm the differentiation between the two groups (besides adding PJP010)? The resolution power of these two is expected to be lower as compared to haplotype-based CP analysis.

Relationship with other populations:

Lines 45-55: MMP individuals demonstrate high genetic diversity, which somewhat contradicts the description in the Introduction, particularly the characterization of this group as a local, sedentary, non-mobile population. The genetic findings from this study suggest that additional analyses (e.g., stable isotopes) may be needed to determine whether the 'outliers' in the PCA show signs of recent immigration into the region. If so, the observed 'turnover' in ancestry in this area may be called into question.

Lines 18-37:

East Eurasian ancestry in MEM individuals: perhaps, authors could use individuals from the Gnecchi-Ruscone 2024 to further explore the suggested "Avar" role in the origin of the East Asian component.

Discussion:

Line 45: If the Prague culture arrived in the Moravia region (as stated here), could the authors clarify from where, based on archaeological findings. The genetic findings are also interpreted in the context of an 'arrival' of people from other (eastern) region.

Lines 54-57: could Authors add here a reference to the analyses (of this study) that supported the complete replacement of the ancestry in MEM individuals. qpADM (in the form it is presented in the manuscript) seem not to support this statement.

Lines 17-18: The authors studied individuals from only two sites, where they observed clear signs of genetic discontinuity between them. Therefore, the findings of this study are not compatible with the idea of autochthonous development in this region. It is important to emphasize that this applies specifically to the studied region. I doubt that the data from this study is sufficient to make broader generalizations.

Methods:

Haplotype-based analyses:

Line 7: what reference panel was used to impute genotypes?

Fig. 2 (both plot and legend): check the population names spelling (e.g. Belarusians)

Replies to the questions from the journal:

- Are the methods appropriate to the aims of the study, are they well described, and are necessary controls included? If not, please specify what is required. YES

- Are the conclusions adequately supported by the data shown? If not, please explain. PARTLY. The main concern is the 'sedentary' nature of the MMP, which is based on the archaeological context. The authors detected high genetic heterogeneity among these individuals, comparable to the Lombards, a group known for its mobility. Therefore, additional data supporting the sedentary nature of the MMP population (e.g., stable isotope analysis) is needed. Alternatively, if some MMP individuals are confirmed to be recent arrivals, this site may not be suitable to claim about ancestry change related to Slavic expansion.

- Are sufficient details provided to allow replication and comparison with related analyses that may have been performed? If not, please specify what is required. YES

- Does the work represent a significant advance over previously published studies? POTENTIALLY YES (but see the comment above)

- Is the paper of broad interest to others in the field, or of outstanding interest to a broad audience of biologists? YES

**Reviewer 4**

**Were you able to assess all statistics in the manuscript, including the appropriateness of statistical tests used?**

Yes, and I have assessed the statistics in my report.

**Comments to author:**

Schulz et al. report novel ancient genomic data of 18 individuals from two sites in South Moravia spanning the migration period and early Medieval, to investigate the formation of the Slavs. The main result of the study is evidence for a substantial genetic shift between the individuals representing the local community during the Migration period and the later Early Medieval period individuals associated with Early Slavic culture. The authors conclude that this shift supports the notion of population movement being a major driver for the Slavic expansion.

Overall, the data and analyses presented support the conclusions of the authors. While genetic shifts associated with Slavic expansions have previously been shown in other archaegenetic studies in both Eastern (Peltola et al Curr Biol 2023, Stolarek et al Genome Biol 2023) and Southeastern Europe (Olalde et al Cell 2023), the novelty of this study is the first reporting of ancient genomes directly associated with early Slavic culture. Furthermore, the authors present high quality genomes which allow them to perform more fine-scale inferences using haplotype-based methods. The results are for the most part presented in a concise and clear way, however I do have a few concerns / comments which I will outline below.


Dataset

The genomic data presented is of high quality, with genomic coverage ranging from 5.1X - 9.4X. I am a bit surprised though that the authors used bwa-mem for read alignment, instead of the normally used bwa-aln. What was the reason for this? Also, as bwa-mem performs local alignment which can lead to spurious mappings for due a smaller number of read bases aligning with the remainder masked out with soft clipping. How were those situations treated?

I was also a bit confused on the different datasets being used in different analyses. In section 4.2.1, the authors describe a set of reference samples that appear to have been processed in the same way as the newly generated samples, i.e. re-mapping from read data in fastq format. The full list in Dataset S5 contains a total of 215 reference samples processed in this way. From what I can gather, the only analyses carried out where those samples are included is the PCAngsd analysis presented in Supp Fig 11-13, but that does not include the Damgaard et al 2018 individuals which appear not be used at all.
For analyses presented in 5.2 (Projected PCA), the authors the use a different panel including also capture samples, on a pulldown of 1240K sites. Confusingly, in section 5.3 (ADMIXTURE) there is again a dataset description that includes a 1240K + HO panel generated by merging the AADR dataset. Presumably that the same merge that has been used for 5.2? Also, despite being described in 5.3, this 1240K + HO panel doesn't seem to have been used for any ADMIXTURE analyses, but mainly for the f-statistics.

It would thus be very helpful if the authors could clarify/streamline those sections, and also include a table with metadata for the public data used in the analyses. The f-statistic section uses population labels like DanubeTisza_early throughout, but there's no straightforward way to link those to the individuals included.

Genetic shifts

This section describes multiple analyses providing evidence for a genetic shift between MMP and MEM samples. The distinction is already quite clearly visible in the projected PCA (Fig. S14), where the MMP samples form a cline towards the eastern Mediterranean, whereas most of the MEM diversity falls within NE Europe/Baltic region. The haplotype-based analyses using chromopainter/fine-structure provide a more fine-grained resolution, clearly demonstrating high diversity within MMP spanning N Europe to S Europe. I am confused about figure S23, which is a fineSTRUCTURE PCA but contains only ancient samples, and from groups not described at all in other analyses (e.g. Upper_Paleolithic_Neolithic). This relates to my previous comments on the different datasets, please clarify whether this analysis was done using haplotype data and if so which individuals were included and how they were imputed/phased.

Within the MEM group, structure is less pronounced but there does seem to be some difference in affinity to SE Europe/Balkans, seen in PCA, finestructure clustering and also in the MOSAIC analyses. It's not really discussed in the main text, but would be worth commenting on. From the chunklength PCA it appears to be the later samples of the MEM group, is this consistent with later gene flow from the Balkan region?

The relate analysis is interesting, but as the authors mention the difference observed is likely a consequence of the higher diversity of ancestry in the MMP group which would be expected to manifest itself in elevated Ne.

Relationships with other populations
This section provides a more in-depth examination of the relationships between MMP / MEM samples and other groups. Using qpAdm, the authors find that at model of >40% contribution of NE European populations is needed to fit MEM as descendants from MMP. While I don't have concerns about this result, the authors state in the supplement that " The only plausible qpAdm models that explain the genetic variability of MEM must comprise MMP as models without MMP and only other populations could be rejected (Dataset S4)". However this statement seems to be not supported in Dataset S4, as all models with MEM as target have MMP as one of the sources (Left population 1). I also don't quite follow how a lack of ancient reference populations from NE Europe would lead to an overestimation of that ancestry.
For the MOSAIC analyses of modern Slavic-speaking populations, why were modern Slavs also included as sources in Fig. S25?

General

My only other general concern is the lack of inclusion of the samples from Stolarek et al in the 1240K analyses. Given the number of individuals and close geographic location of origin, their inclusion with the newly presented data would provide valuable context to the broader regional impact of the Slavic expansions.

Minor comments
The reference panel used for imputation should be mentioned. From the file names it looks like 1000G but not explicitly stataed

Figure 2A is missing a legend for the color scale

The symbols for the modern samples in Fig 2B are very small

In fig S10 it is very hard to distinguish samples due to small symbols and/or similar colors
Similary, figs S11-13 would benefit from larger labels and less white space. Using the same colors for the new samples as in the main figures would also be helpful

In Fig S14 the legend seems to be switched for the LIB7,11 and other MMP samples. Labels for the individuals of the new samples would also be helpful

Figs S16/S17 show outgroup f3, but the scale seems to be wrong? I.e. the f3 value for the highest sharing population discussed (e.g. Lithuania_BA for MEM) is the lowest on the axis

In the analyses of Avar ancestry, POH_Avar samples are defined as "outlying ancestry" in PCA, does that refer to the projected PCA? As mentioned above, labels would be helpful there.

**Authors Response**

In the following our responses to reviewers *start with ** and the directly referenced parts of the main text are in ***quotes “..”***.

**Reviewer #1**

In the paper "Ancient genomes from Moravia evidence demographic spread of Slavic-associated groups", Schultz et al analysed high-quality genomic data of 18 individuals from South Moravia, from a crucial region and time frame to shed light and to understand the genetic features of the Slavic expansions.

The samples are very interesting and their high quality allows to perform interesting analyses, that the Authors indeed performed, but not always adequately discussed.

**We are grateful for this positive feedback and happy to see that the reviewer sees merit in our samples and analyses.*

However, the way the manuscript is presented now makes it difficult to understand the work and its main results and in some cases the Authors' conclusions seem not to be supported by the data (or maybe the link between the data and the conclusions is not sufficiently highlighted). In addition, often the relevant supplementary data are not cited in the main text, so it is really hard to check the discussed results. Overall, the manuscript seems to lack coherent organisation and it is hard to follow the logical steps from data analysis to results to discussion.

For these reasons, I believe that this paper can be suitable for publication after major revisions under both the presentation and the interpretation point of view.

**We are sorry to read that the reviewer found our manuscript difficult to follow and have implemented a series of the suggestions received to improve on that.*

MAJOR POINTS

1) The Authors state that they analysed 54 individuals, but then increased the coverage for 17 of them. However, they do not report the sequencing information for all the 54 individuals: this is not acceptable, both in general in the aDNA field and especially since the Authors claim that the samples to analyse at higher coverage have been selected among those with an endogenous DNA content > 20%. How can this be checked if no details are reported?

**We fully agree that all data produced needs to be presented and shared and apologize that the screening data was not readily available. We now added a new supplementary data table providing complete information regarding all samples processed and also made the screening results available on ENA.*

2) In addition to the previous point, the Authors should give some more information about their choices regarding the samples to analyse at higher coverage. In the text, they say that the 17 samples represent a cross-section of the social structure but no sample-by-sample information about this is provided (or can be easily found). Adding this to Dataset S5 would be useful.

**Thank you for this remark. We have amended Dataset S5 (now renamed S1) with additional information, in particular there are now columns for archaeological ID, the sub-site the grave has been discovered at and an archaeological summary of the context. From the presented information, it is clear that the selection of the samples largely driven by the preservation also represents a balanced selection between different archaeological subsites (interpreted as potentially linked to different sections of the society) and between different levels of social strata (as based on the grave goods attached to the skeletal remains).*

Moreover, dataset S5 includes three sheets: what is the difference between sheet 1 and 2? Some more explanations as table caption should be added (this is true for all the tables and datasets).And why are some published samples present too? I could not find any mention to them in the main text and there is just a brief mention in the supplementary.

**The two sheets provide summary statistics per sample (first sheet, now called WG Results) and per fastq file (second sheet, now called WG Results Per File). The two sheets are thus identical for samples processed from a single fastq file, but contain complimentary information for samples processed from multiple files. In addition, the files list the same statistics for all samples we re-processed from raw files. We have now made that more clear in the description of the resource. The list of additional files and their description can be found after References in the Main text (as suggested by the journal).*

3) The supplementary information includes a huge amount of data, some necessary to understand the results reported in the main text, some others not mentioned at all in the main text (e.g. all the section about uniparental markers). Since the journal does not have a strict word count, I suggest to the Authors to move some data in the main text to let the readers find the relevant information in a faster/easier way, especially regarding the methods.

**We agree with this suggestion and have now integrated all method related descriptions into the method section of the main text. We also added summaries of all previously undescribed analyses to the main text.*

Moreover, if the data in supplementary are not mentioned at all in the main text, maybe the Authors should decide if they are really relevant (and then some words in the main text should be added - e.g. can the Y chromosome haplogroups give any additional input regarding the Eastern Asian ancestry reported in lines 18-37, page 8?) or if they should be deleted. Finally, some plots seem to be duplicated between the main text and the supplementary file (e.g. Suppl Fig 22 and Fig 2B but other instances may be present): this must be avoided and fixed.

**We have now mentioned the results of uniparental markers analysis in the main text: “…****and we found no uniparentally inherited markers linked to East Asia (SI 3.6, Supplementary Fig. 26, Additional file 4)”.*** *Only the results of analyses essential for the conclusions of the study are now included in the supporting text. We have further deleted all duplicated figures from the supplementary file.*

4) The Authors' choice to relegate the "standard" projected PCA in the supplementary is curious: what is the reason? The "standard" PCA is something quite common in the field that can help the readers to visualise the data in a known context: maybe the authors can consider including both PCAs (projected and haplotype-based) as main figures.

**While the ‘standard’ projected PCA does not provide enough power in the regional context considered here, we agree with the reviewer that it is common practice to show it and have hence added it to the new Fig. 2.*

5) The low coverage samples can indeed be used for some analyses, including the projected PCA (in some papers, a coverage as low as 0.04x or even 0.02x is considered sufficient): since the Authors did this analysis, why don't they include as many samples as possible? Although low-coverage samples cannot be used for haplotype-based analyses, including them in the frequency-based analyses can help to elucidate the general picture. For example, based on 7 samples the Authors claim that the MMP samples are really diverse along a cline. This is reasonable according to the literature, but how can you state it from only 7 samples? Making an extreme example, it is possible that you only collected outliers by chance. The positions of the other samples in the PCA can give you additional support. The same is true for f3, f4, qpWave/qpAdm, etc.

**One would always wish to have a larger sample size. However, some extreme events are indeed detectable with a low sample size. In our case, we had a total of 17 samples, of which 5 date to an earlier period and form their own genetic group (MMP). The reviewer was concerned that such a genetic grouping could just occur by chance. However, the probability that all five samples dating to the migration period are outliers is actually only 0.0001616 (only one out of 6188 ways to partition the 17 samples into a group of 5 and 12 samples). This value is equivalent to the p-value under a X^2^-test on these two groupings (time and genetics). It is thus safe to conclude that these results are not a chance event with p=0.0001617.*

**That said, we do agree that additional samples provide additional confirmation and insight. Unfortunately, our screening data only resulted in a mean depth of 0.0008x per sample (or 0.004x if you include the samples that underwent additional sequencing later on), which does not allow for per-sample analysis (none of these tests were significant).*

**But in light of this comment, we repeated two analyses using all samples that could be reliably attributed to either group (dated securely with archeology or were ^14^C dated):*

1) *We re-ran the qpWave analysis indicating that our MMP and MEM samples draw from distinct ancestries, qpWave(MMP_WithLowDepth and MEM_WithLowDepth), which was significant* *(p = 1.52E-026)(Population definitions, Additional file 2)****.***
2) *We re-ran f4 statistics to identify the populations that break the MMP / MEM cladility. Results were very similar and are now shown in the SI. Affinities to the same or very similar populations.*

**In both cases showing that our major conclusions are supported also with the larger dataset (though likely the results are mainly based on the originally analysed data). We now mention these results in the SI 3.3 and 3.4.*

6) In the Discussion, the Authors often support the hypothesis of a complete population replacement in the region but this is not supported by their own data. In the first paragraph of the discussion, the Authors claim: "Conservative estimates given existing reference populations, however, suggest that the contribution of the immigrating population was dominant or even completely replaced the local population in this region" (lines 54-58, page 8). Not only there are no results supporting this, but the data support a contribution of MMP to MEM (as the Authors themselves state a few lines before, lines 4-5, same page), thus clearly rejecting the hypothesis of a complete replacement.

**In our case, qpWave clearly rejected complete continuity between MMP and MEM samples. We therefore used qpAdm to explore different models to explain the ancestry found in our MEM samples. It is important to note, however, that qpAdm can never be used to prove a contribution. Instead, it can only be used to reject models that lack an important ancestral source. The reason is that the true source is likely absent from the reference populations explored. The fact that the only qpAdm models for MEM samples that were not rejected included MMP could indeed be due to a contribution of MMP, but also arise because we were lacking the right source for the MEM samples. Given the few reference populations available, it appears very likely that we lack the proper source and that the true source was closer to MMP samples than the distant sources we had available in the particular setting. To illustrate this, we now conducted additional apAdm analyses (including recently published papers studying different regions in adjacent periods) and found additional models for MEM that were not rejected but did not include MMP samples.*

**Hence, a reasonable interpretation of the qpWave results is that the 44% (51.8% with newly found plausible model) MMP contribution is the upper bound of the uncertainty regarding that contribution, to be expected once better source populations become available. So while qpWave analyses rule out direct continuity from MMP to MEM, they can actually not be used to rule out a complete replacement and we need to consider other analyses. Taking all evidence together, we stand behind our interpretation of “****major population shift in most localities, followed by the assimilation of few remaining individuals of the previously local population, mobility from neighboring regions (e.g. from regions associated with Lombards or Franks, 70, 71), or a combination of both.****”*

Similarly, in lines 32-54 page 9, they state that the Slavic became more heterogeneous over time, but their data show more ancestry diversity in previous periods (MMP) rather than in later ones (MEM).

**We fail to understand the contradiction in this. Clearly, MMP samples were more heterogeneous than MEM samples, but that does not rule out that MEM samples themselves became more heterogeneous over time (albeit never reaching MMP levels).*

All the Discussion needs to be rewritten to take into account what the results show (or this section needs to be better explained, if my interpretation is not what the Authors meant).

**As argued above, we are convinced that our results do support our conclusions.*

MINOR POINTS

Introduction

Lines 41-48, page 5: It would be useful to cite Fig 1 here already.

**Done.*

Lines 46-52, page 4: This sentence seems incomplete (from "indigenous").

**We rephrased this sentence slightly to make it more readable.*

Lines 19-20, page 5: A dot is missing after Balkans.

**Indeed! Thanks for spotting this.*

Results

Line 19, page 6: What do the authors mean with "shallow whole genome sequencing"? A different term should be used instead of "shallow".

**We now use the term “low-depth” and provide the actual depth obtained.*

Line 22, page 6: Dataset S5 is mentioned, but no mention to Datasets S1-4 has been made before: the supplementary should be named in the order they appear in the text (and all the supplementary should be cited, but for example I could not find any mention to dataset S1).

**We now changed the numbering of all supplementary data sets, tables and figures to match the order in the text and also ensured that all material is cited in the main text.*

Line 11, page 7: "albeit noiser due to lower power", what do the Authors mean?

**We meant to say that the inference of PCA location is noisier due to lower power, and hence the grouping appears less obvious. We now rephrased to:*

***“..., although the samples appear more scattered due to lower power.”***

Line 23-24, page 7: the expression "direct continuity" is misleading here, because MEM seem to descend from MMP + additional sources (and so some sort of "direct" continuity can be indeed observed). The Authors should rephrase this sentence.

**We agree this terminology may not be appropriate and we changed to “simple continuity” to highlight that the MEM samples are not just the descendents of MMP samples.*

Lines 26-41, page 7: Basing the differentiation between MMP and MEM on the Relate data seems to be an overinterpretation of this result. The difference date back to 6000 BCE (so, approximately 7 millennia before the period under study) and, as the Authors themselves state, the higher Ne of MMP can result from individuals of different ancestries. I wonder if including more samples (i.e. using tools - such as ASCEND <https://github.com/sunyatin/ASCEND> - that can deal with low coverage aDNA data and including the other MMP and MEM individuals) can give different results. If so, I suggest removing this part from the main text (especially the figure 3) and/or tuning it down.

**We agree with the reviewer that the Relate analysis is unlikely reflecting the full complexity of the relationship between our MMP and MEM samples, and we also never intended to suggest it does. However, these results do show that the populations are somewhat distinct and does reject a model of simple continuity - while leaving room for a variety of alternative scenarios. We therefore decided to keep the analysis in the text but have amended the description to now read:*

***“Although the simple split-model of Relate is unlikely reflecting the complex relationship between these populations, the result does support the notion that these populations have distinct histories and rejects a simple model of continuity.”***

**As discussed above, the depth of our screening samples does not amend them for the type of additional analysis suggested.*

Lines 43-56, page 7: I cannot understand the logic behind this. Why should they form a clade if they are so diverse including individuals from different ancestries? Is it not exactly what we would expect? Maybe f3 and f4 statistics would be a better tool to investigate the relationship between Longobard, MMP and MEM, both by groups or specific individuals. Also a comparison with Avars can be informative?

**We have included f4(Outgroup, Lombard_period_population; MMP, MEM) where Lombard_period_population is either Szolad, Collegno, or Slovakia_Germanic_MP and added the results of this analysis to SI 3.3. None of these published Lombard populations break the clade of MMP and MEM, suggesting that the similarity of MMP to them is not significant.*

**The comparison with Avars is included in the Relationship with other populations section of the main text and other sections of SI.*

Line 59: again, the expression "direct descendants" should be rephrased.

**We now rephrased to:*

***“Since the MMP samples alone are not a good proxy for the ancestry of MEM samples, …”***

Lines 31-32, page 8: a closing bracket is missing.

**Done*.

Lines 11-13, page 8: since the Authors performed chromopainter/fineSTRUCTURE, can the MOSAIC results be confirmed by GLOBETROTTER/SOURCEFIND?

**Unfortunately, the painting we got with chromopainter is not suitable to run GLOBEROTTER/SOURCEFIND and we would need to re-paint all chromosomes for that analysis. We question whether that effort is worthwhile, particularly since GLOBEROTTER/SOURCEFIND and MOSAIC usually agree and are based on the same underlying model. In addition, other studies have estimated a similarly small East-Asian contribution of 1-3% when using modern samples (Hellenthal et al. 2014, Salter-Townshend & Myers 2019) and we are unlikely to provide additional precision on the exact contribution with our samples. Finally, and given the small East-Asian contribution, a much larger sample size would be needed to disentangle different East-Asian sources. We hope the reviewer agrees, but remain open to embark on that extra effort if the reviewer judges this analysis as essential.*

Figures

Figure 1, panel B: I am not an expert of the Moravia historical periods. Is the chronological order: Migration period, Prague culture, Early Hillfort and Middle Hillfort? Considering the importance of this for the data interpretation, maybe it would be helpful to add also this information to the timeline in this panel (in addition to the useful Migration period/Early Middle Ages labels already present).

*Yes, we added this chronological differentiation to Fig. 1.

Figure 2, panel C: its utility and meaning is not clear, I suggest to remove it; a sentence in the text is sufficient.

*We followed this suggestion and removed that panel.

Supplementary Data

Supplementary figures 11-13: the images are at very low resolution and the colours are hardly distinguishable.

**We have replotted those figures to make them more readable.*

Supplementary figure 14: the symbols for LIB7, LIB11 and MMP seems to have been switched in the legend.

**Thanks for spotting this - we have now harmonized the symbols.*

## Reviewer #2

This manuscript provides an important contribution to the debate regarding the demographic history of Slavic-associated populations in Central Europe, presenting genomic data from two burial sites in South Moravia. While the study introduces new data to an underexplored period, there are several concerns regarding the interpretation of results, particularly in terms of the proposed genetic turnover and population movement.

**Thank you very much for this positive feedback.*

Key Points of Concern:

The conclusions regarding a major genetic turnover between the Migration Period and Early Medieval populations are drawn from a relatively small sample size of 18 individuals. While these samples are of high quality, the limited number of individuals, all from two sites, restricts the broader applicability of the findings. Given the small sample size, it is difficult to generalize these results to broader population dynamics in Central Europe, let alone infer a large-scale genetic turnover. A more extensive dataset from a wider range of sites would provide a stronger basis for the conclusions drawn.

**While we agree that a larger sample size is always providing more information, we still see a lot of value in our targeted approach. In particular, the small geographic scale allows us to trace the events in chronological order without the need to make assumptions about contemporary events that is often the case when working with samples from large geographic areas. But of course that does limit the generality of our findings, yet we do believe that a genetic turnover in one region may be strong evidence, albeit not proof, for genetic turnover in the region.*

The study presents a substantial temporal gap, particularly in the 6th century, a period critical for understanding the early phases of Slavic expansion. Most of the individuals in the Early Medieval sample date to after the 7th century, while the Migration Period samples date to the 5th century. Without sufficient representation from the 6th century, it becomes difficult to determine whether the genetic changes occurred gradually or as a result of abrupt events such as migration. This lack of temporal continuity limits the study's ability to trace the exact nature of population shifts, and additional sampling from this gap would enhance the analysis.

**The reviewer is certainly right that there exists a considerable and unfortunate temporal gap between the latest samples from the Migration Period and the earliest samples from the Early Medieval Period. However, that gap is not due to a lack of sampling, but because cremation was the primary funerary rite in the region during the 6th century. While there is thus little that can be done to close this gap, we stress that our study closes this gap considerably when compared to existing studies, not least because the sample PJP10 from the Pohansko site, is the earliest known inhumation associated with the Prague culture.*

**We note that there is a short horizon associated with Lombard material culture in the broader region. Lombards are thought to have been present transiently only and moved south from Moravia in the 6th century. The 5th century samples included here are thus thought to be a more relevant comparison for genetic shifts in Moravia. We further confirmed this interpretation by showing that neither our MMP nor our MEM samples are well represented by Lombards from other sites, be it Hungarian (Szolad, qpWave p-value: 2.18E-12 (MMP) and 9.94E-54 (MEM)) or Slovakian (Tesarske Mlynany, qpWave p-value: 5.29E-13 (MMP) and 7.77E-09 (MEM)).*

One significant issue is the broad temporal range (7th to 10th century CE) over which the Early Medieval samples are spread. Despite this, the study treats the Early Medieval individuals as a largely homogeneous group, which may oversimplify the genetic and social changes that occurred over this 300-year period. These centuries were marked by significant political and cultural shifts, and pooling the Early Medieval samples together risks overlooking important intra-period variation. Differentiating between the earlier and later Early Medieval samples could offer a more nuanced understanding of genetic continuity and change over time, avoiding overly broad conclusions about population stability or replacement.

**We initially pooled these individuals because they appear rather homogenous genetically as attested, for instance, by the chunk length PCA, but also because of the rather homogenous archeological material culture with a steady local development at the site, which was continuously occupied from the 6th to the 10th century. The most significant cultural change appears to have occurred at the turn of the 8th and 9th centuries, when the local population of Pohansko began to be inhumated, probably under the influence of Christianity (see also PŘICHYSTALOVÁ, Renáta. Die Bestattungen in Břeclav-Pohansko. Alte und neue Ausgrabungen. In Macháček, Jiří - Ungerman, Šimon. Frühgeschichtliche Zentralorte in Mitteleuropa. Studien zur Archäologie Europas, Band 14. 1. vyd. Bonn: Verlag Dr. Rudolf Habelt GmbH, 2011, s. 35-61. ISBN 978-3-7749-3730-7.)*

**However, based on this comment, we now conducted additional analysis to verify the homogeneity of the MEM cluster. Specifically, we split the MEM samples into early and late groups based on ^14^C dating (except for POH39) and then used qpWave to assess their cladility. Despite using a large number of reference populations, none of them was able to break the cladility between the two MEM groups. While that does not rule out interesting demographic changes over the studied period, to uncover such events would need a much larger sample size.*

The observed genetic differentiation between the Migration Period and Early Medieval individuals is interpreted as evidence of a major population movement. However, this interpretation might be somewhat overextended. Genetic shifts can result from a variety of factors, including internal social dynamics, marriage patterns, or selective gene flow, rather than outright population replacement. While migration is one possible explanation, the dataset does not provide unequivocal evidence for this scenario, and alternative processes should be considered more carefully. A more cautious interpretation of the data, acknowledging other potential drivers of genetic change, would strengthen the study.

**We agree with the reviewer that the migration may not necessarily have happened as a one-generation replacement event, but could have occurred across multiple generations instead. We assume this is what the reviewer meant by marriage patterns and selective gene flow and have updated our wording accordingly. Nonetheless, we emphasize that such a turnover must have occurred rather rapidly (within less than 200 years or seven generations). In addition, the genetic shift we observe coincides with a cultural shift including the founding of new villages, archeological evidence that also appears to point to a somewhat disruptive process.*

The connection drawn between genetic findings and the spread of Slavic-associated groups remains speculative. The study suggests that the genetic changes observed could be linked to the arrival of Slavic-speaking populations, but the evidence for this claim is indirect. Material culture, such as Prague-type pottery, does not necessarily imply linguistic or ethnic continuity. Without concrete historical or linguistic data directly tying the individuals in the study to Slavic identities, the conclusion that this represents the spread of Slavic-speaking groups should be considered carefully and framed as a hypothesis rather than a definitive finding.

**We fully agree that linking cultural artifacts with ethnicity or language use is difficult, as we also discuss at great length in our introduction. However, we believe that the samples presented here are as close to establishing such a link as possible, in particular since the Prague-type pottery is Slavic-associated based on archaeological and historical records, albeit in a bit later periods than our samples. Specifically, the majority of the sampled Slavic-associated burials are associated with Great Moravian material culture and are dated to the 9th century. At that time, Pohansko was a prominent centre of the Great Moravian polity (Macháček 2010), where the Slavic language was first used in written form (Cubberley 1993; Marti 2014). The site's origins can be traced back to the 6th century, when a settlement with Prague-type pottery and Slavic cremation burial grounds was established. The first known inhumation associated with the Slavic-speaking population (grave no. 205) was that of a newborn child buried in a pit alongside settlement refuse, including sherds of vessels that were typologically linked to both Prague-type and later Great Moravian pottery.*

**Nonetheless, we agree that our interpretation remains just that, an interpretation, and we have updated our wording in the final paragraph to more carefully reflect that fact.*

The study notes the challenge posed by the lack of reference populations from North-Eastern Europe, which limits the ability to precisely contextualize the findings. The absence of comparable ancient DNA data from nearby regions makes it difficult to determine whether the genetic shifts observed in South Moravia were part of a broader regional trend or a localized event. Incorporating more comparative datasets from surrounding regions and time periods would provide a clearer understanding of how the genetic changes in South Moravia fit within the broader population history of Europe.

**Our manuscript provides compelling evidence for a major genetic shift in Southern Moravia. We certainly agree that a larger sample from a larger geographic region would help in establishing the extent to which the shift observed in Southern Moravia was part of a broader trend. However, we believe that the localized context is actually a major strength of our study in that it allows us to compare populations through time without the confounding effect of space. To close the gap between late migration period and early medieval samples using available data, for instance, samples have to be compared between rather distant sites. That is particularly problematic if the processes under study were variable in time between different locations as was suggested for emergence of Slavic groups historically and archaeologically. Nonetheless, our findings are in good agreement with a pattern visible in the analysis of Stolarek et al. 2023 and Olalde et al. 2023, suggesting that the shift, for which we now provide a localized yet chronologically refined evidence, did occur in a broader region.*

This manuscript provides valuable insights into the genetic history of early medieval populations in South Moravia, contributing new data to the study of Slavic-associated groups. However, the small sample size, significant temporal gaps, and lack of differentiation among Early Medieval samples complicate the interpretation of genetic turnover. The conclusions regarding large-scale migration and population replacement should be approached with caution, given the multiple potential drivers of genetic shifts. More robust sampling and the inclusion of additional comparative data would help clarify the nature of population dynamics in this region. Further research is needed to fully understand the genetic and cultural transformations of early medieval Central Europe.

**We are happy to see that the reviewer sees value in our localized study. We fully agree that our finding of a major genetic shift in Southern Moravia is only hinting at a broader pattern, which would need to be confirmed with studies from other regions. And while we made an attempt to clarify this view, we nonetheless believe that our results provide a good basis to speculate that such a genetic shift was likely associated with the spread of Slavic culture.*

## Reviewer #3

I would thank authors for putting together a nice, structured and concise manuscript. I enjoyed reading it!

**Thank you very much for this very positive feedback!*

I have a few small comments and one concern. I deeply hope that they might help to strengthen the conclusion authors make.

**Title:**

I suggest editing the title to specify the region the authors are referring to when they mention 'demographic spread.' In its current form, it could be interpreted as applying to the entire Slavic area. While this might be true for the studied microregion in Central Europe, there is not yet sufficient data to generalize this to a wider area.

**We agree with this assessment and have changed our title slightly to*

***“Ancient genomes evidence demographic spread of Slavic-associated groups in Moravia”***

**Introduction:**

Line 9 (However, archaeological evidence …): This statement is questionable because there is no consensus among archaeological schools about which archaeological culture should be associated with the Slavic tribes. Additionally, the Prague culture is not homogenous.

**We agree that there exists some disagreement. We added a qualifying statement to this sentence and relevant references about complex relationship between archaeology, genetics, history and identity:*

***“However, while linking archeological finds to identities is challenging, archaeological evidence suggests that the Slavs were present in large parts of Central Europe several generations earlier (5–7). “***

**Results:**

Line 2 (North-Western Slavs such as ..): Lithuanian are Baltic-speaking population, not Slavs.

**We fully agree with this and apologise for this mistake. We only meant to imply that Polish are North-Western Slavs, but agree it was not formulated clearly and have thus changed to the term “North-Western Europeans” used for both groups.*

PCA projection and PCA on likelihoods: could authors provide their motivation in using these two approaches to confirm the differentiation between the two groups (besides adding PJP010)? The resolution power of these two is expected to be lower as compared to haplotype-based CP analysis.

**We fully agree that the haplotype based analyses have much higher power. But as the reviewer states, these methods did not allow us to include PJP010 due to its DNA preservation. Given that this sample constitutes the earliest inhumation associated with the culture, a PCA analysis that allowed us to involve that sample seems appropriate. We further note that the haplotype-based methods are not easily applied to many of the ancient samples. We therefore believe that these two approaches are helpful by being both complementary to the haplotype-based analyses in terms of samples that can be included, and additionally serve the purpose to confirm that the patterns we describe can be found regardless of the exact methodology used. That seems particularly relevant in light of a comment made by Reviewer 1 that now asks us to even include the projected PCA into the main text.*

**Relationship with other populations**

Lines 45-55: MMP individuals demonstrate high genetic diversity, which somewhat contradicts the description in the Introduction, particularly the characterization of this group as a local, sedentary, non-mobile population. The genetic findings from this study suggest that additional analyses (e.g., stable isotopes) may be needed to determine whether the 'outliers' in the PCA show signs of recent immigration into the region. If so, the observed 'turnover' in ancestry in this area may be called into question.

**We agree that our results do spark interest in 5th century period in the region and will lead to further analysis (eg. isotopes). We explicitly tried to focus on the population thought to be the least transient in the region at the time: Líbivá is dated to the beginning of the Migration Period, is characterised by mixed archaeological features consisting of different cultural components (e.g. East-Germanic, Alano-Sarmatians, Suebian, Vandalic) and cannot be considered an elite site. In line with this, the genetic diversity observed is similar to that observed at other sites of that period (Antonio et al. 2024, Vyas et al. 2023), suggesting that our samples are indeed representative of the genetic make-up in the region.*

**It is also important to note that the ancestry of MEM samples is distinct from the whole distribution of ancestries observed for MMP samples. So even if some of the MMP samples turned out to be recent immigrants or transient, it would not undermine our conclusion of a major turnover.*

**The population generally thought to have been transient in the region at that time were the Lombards. We explicitly compared both published Lombard period populations (from Hungary and Slovakia) to both MMP and MEM populations and found that neither cladded with Lombards.*

**We can of course not completely rule out that the genetic ancestry found in MEM samples was already present in the 5th century in a local and very distinct population that left no known archeological trace and was not sampled in any of the archaeogenetic studies in the wider region. However, that appears extremely unlikely.*

Lines 18-37:

East Eurasian ancestry in MEM individuals: perhaps, authors could use individuals from the Gnecchi-Ruscone 2024 to further explore the suggested "Avar" role in the origin of the East Asian component.

**We have added the samples from that study to gain power on the ancestry of the East Asian component, but as expected, it did not yield further insights as East Asian individuals from the sites we studied at the DTI region were largely mixed with local populations.*

**Discussion**

Line 45: If the Prague culture arrived in the Moravia region (as stated here), could the authors clarify from where, based on archaeological findings. The genetic findings are also interpreted in the context of an 'arrival' of people from other (eastern) region.

**The defining characteristics of Prague culture are settlements comprising sunken-floored huts built on a square plan, an economic structure based on autarchic agriculture, underdeveloped technology, distinctive elements of the social system, customs and beliefs, a funerary rite, a lack of foreign influences and a general standard of living that is relatively low. Many archaeologists argue that the great Slavic expansion began in central and upper Dniestria, in what is now Ukraine or Belarus, because archaeological findings from the second to fifth centuries in this region are in many aspects identical or similar to findings in central and south-eastern Europe from the fifth to sixth centuries. However, this is quite heavily disputed and since our samples can not shed light on the question of the source area (neither genetically nor culturally), we prefer not to delve into these discussions in the text.*

Lines 54-57: could Authors add here a reference to the analyses (of this study) that supported the complete replacement of the ancestry in MEM individuals. qpADM (in the form it is presented in the manuscript) seem not to support this statement.

**Estimating the exact contribution of the local MMP population to our MEM samples is difficult due to an apparent lack of reference populations representative for the true source of the MEM ancestry. Given the available reference populations, there are some qpAdm models we can not reject that indeed include MMP samples. While this may suggest a true MMP contribution, it appears at least as plausible that that contribution arose as an artifact because the true source population for MEM shared a component with MMP that is absent from the closest available sources.*

**One qpAdm model discussed in the main text involves MMP and Lithuania_Marvele_Roman, with a MMP contribution of 51.8% as this is the model with the largest MMP contribution from the plausible models obtained (with the populations newly added to the analysis the number of models that cannot be rejected increased). But given that Lithuania_Marvele_Roman predates MMP, it appears indeed very plausible that the true source for MEM had somewhat different ancestry that Lithuania_Marvele_Roman and that this missing ancestry was similar to MMP. This interpretation is further supported by the finding that MMP can also serve as a proxy for that missing part of the ancestry for samples from much further east such as the Volga-Oka region, for which a direct contribution of MMP can be safely excluded. In addition, we note that with the additional reference samples now included, there are several qpAdm models for MEM not rejected that do not require MMP. We now provide this reasoning more carefully in both the discussion and the results section.*

**In summary, and while we do not have evidence to prove a complete replacement, such a replacement appears plausible given our results, and hence our statement in the Discussion that “****a complete population replacement in this region cannot be excluded****”, which we believe is fair.*

Lines 17-18: The authors studied individuals from only two sites, where they observed clear signs of genetic discontinuity between them. Therefore, the findings of this study are not compatible with the idea of autochthonous development in this region. It is important to emphasize that this applies specifically to the studied region. I doubt that the data from this study is sufficient to make broader generalizations.

**We are grateful to read that the reviewer agrees with our interpretation of a major genetic discontinuity in the studied region. We also agree that our samples are far from proving a major genetic discontinuity on a larger geographic scale. In light of this comment, we are now emphasizing the regional nature of our study even more carefully. However, and especially when accounting for other available data, we do believe that our results allow us to speculate about the regional processes as we do in the discussion, where we clearly state that the model we put forth is just a proposition and is suggested for this region.*

**Methods**

Haplotype-based analyses:

Line 7: what reference panel was used to impute genotypes?

**We have now moved the citation for the reference panel to the main text (“****30x 1000 genomes dataset was used as a reference panel (59)”****).*

Fig. 2 (both plot and legend): check the population names spelling (e.g. Belarusians)

**Thank you for spotting this, we have corrected the spelling errors in the text and figures.*

Replies to the questions from the journal:

- Are the methods appropriate to the aims of the study, are they well described, and are necessary controls included? If not, please specify what is required. YES

- Are the conclusions adequately supported by the data shown? If not, please explain. PARTLY. The main concern is the 'sedentary' nature of the MMP, which is based on the archaeological context. The authors detected high genetic heterogeneity among these individuals, comparable to the Lombards, a group known for its mobility. Therefore, additional data supporting the sedentary nature of the MMP population (e.g., stable isotope analysis) is needed. Alternatively, if some MMP individuals are confirmed to be recent arrivals, this site may not be suitable to claim about ancestry change related to Slavic expansion.

*It appears the reviewer is questioning whether our MMP samples are representative of the local gene pool prior to the genetic shift we report because our MMP samples (or at least some of them) could represent a transient population in the region.

*As the reviewer writes, the key transient population in the 6th century were the Lombards, but as we show, Lombard samples do not clade with either MMP nor MEM samples. This indicates that our MMP samples were not Lombards, but also that the genetic shift observed cannot be explained by the ancestry Lombards brought to the region.

*There is also good evidence that supports the notion that our MMP samples represent the relevant genetic make-up of the local population at that time. First, we stress that the genetic diversity observed at the site was similar to that at other sites at other regions at the time, eg. close to Balaton lake (Vyas, Koncz et al. 2023). Second, the Líbivá site, in line with its genetic diversity, is characterised by mixed archaeological features consisting of different cultural components typical for local populations (e.g. East-Germanic, Alano-Sarmatians, Suebian, Vandalic) but given the grave goods present, it was unlikely an elite site. Third, genetic diversity observed for MEM samples is not represented by any of the MMP samples (Fig. 1A and 1C in particular). Hence, and while we cannot rule out that some of the MMP samples were recent arrivals, the observed genetic discontinuity cannot be an artifact of recent immigrants. Finally, we stress that it appears extremely unlikely that there was a population genetically largely unaffected by the Migration period that survived centuries in archaeological invisibility and then morphed into the MEM population observed.

- Are sufficient details provided to allow replication and comparison with related analyses that may have been performed? If not, please specify what is required. YES

- Does the work represent a significant advance over previously published studies? POTENTIALLY YES (but see the comment above)

- Is the paper of broad interest to others in the field, or of outstanding interest to a broad audience of biologists? YES

## Reviewer #4

Schulz et al. report novel ancient genomic data of 18 individuals from two sites in South Moravia spanning the migration period and early Medieval, to investigate the formation of the Slavs. The main result of the study is evidence for a substantial genetic shift between the individuals representing the local community during the Migration period and the later Early Medieval period individuals associated with Early Slavic culture. The authors conclude that this shift supports the notion of population movement being a major driver for the Slavic expansion.

Overall, the data and analyses presented support the conclusions of the authors. While genetic shifts associated with Slavic expansions have previously been shown in other archaegenetic studies in both Eastern (Peltola et al Curr Biol 2023, Stolarek et al Genome Biol 2023) and Southeastern Europe (Olalde et al Cell 2023), the novelty of this study is the first reporting of ancient genomes directly associated with early Slavic culture. Furthermore, the authors present high quality genomes which allow them to perform more fine-scale inferences using haplotype-based methods. The results are for the most part presented in a concise and clear way, however I do have a few concerns / comments which I will outline below.

*We thank the reviewer for this very positive feedback and are happy to read that they see our samples and work as a valuable contribution.

Dataset

The genomic data presented is of high quality, with genomic coverage ranging from 5.1X - 9.4X. I am a bit surprised though that the authors used bwa-mem for read alignment, instead of the normally used bwa-aln. What was the reason for this? Also, as bwa-mem performs local alignment which can lead to spurious mappings for due a smaller number of read bases aligning with the remainder masked out with soft clipping. How were those situations treated?

**We are happy to read that the reviewer judges our data as of high quality. We have been using bwa-mem successfully for quite a while in our pipeline and found it to perform desirably at lower computational costs. We are aware of some studies comparing bwa-mem vs bwa-aln that reported higher mapping rates with bwa-aln, but we failed to replicate these findings in the past once reads were compared after all our filters were applied (as opposed to just comparing raw counts). We are also aware that bwa-mem allows for soft-clipping, and while this may indeed lead to spurious mapping, it also allows for the identification of reads where adapter-trimming failed. To balance this trade-off, our ATLAS pipeline allows for two filters to avoid the issues of spurious mappings: The first is a soft-clip fraction filter which discarded all reads for which soft-clipping bases make up more that an allowed fraction of bases. The second is a strict filter that ignores all reads that have any soft-clipped bases (filterSoftClips). In this study, we applied this second, more stringent filter for all our analyses.*

I was also a bit confused on the different datasets being used in different analyses. In section 4.2.1, the authors describe a set of reference samples that appear to have been processed in the same way as the newly generated samples, i.e. re-mapping from read data in fastq format. The full list in Dataset S5 contains a total of 215 reference samples processed in this way. From what I can gather, the only analyses carried out where those samples are included is the PCAngsd analysis presented in Supp Fig 11-13, but that does not include the Damgaard et al 2018 individuals which appear not be used at all.

For analyses presented in 5.2 (Projected PCA), the authors use a different panel including also capture samples, on a pulldown of 1240K sites. Confusingly, in section 5.3 (ADMIXTURE) there is again a dataset description that includes a 1240K + HO panel generated by merging the AADR dataset. Presumably that the same merge that has been used for 5.2? Also, despite being described in 5.3, this 1240K + HO panel doesn't seem to have been used for any ADMIXTURE analyses, but mainly for the f-statistics. It would thus be very helpful if the authors could clarify/streamline those sections, and also include a table with metadata for the public data used in the analyses.

**We apologize that our description of data sets used was not straightforward.*

*We used three different datasets: 1) The first that allowed us to analyze the WGS samples using genotype likelihoods. 2) The second allowed us to include the PJP010 sample data that is 1240K captured. 3) The third was used for haplotype inference in chromopainter. This last data set differs from the former because chromosome painting requires a higher quality reference and because that reference was already preprocessed.*

**We have now updated the text to make these uses much more clear in the methods section, which now also includes most parts previously presented in the supplementary information. To streamline the presentation, we also removed samples not relevant for the analysis. However, we did not include the same rich metadata for the large number of public data used - we feel this would be overwhelming. But we remain open to amend if the reviewer insists.*

The f-statistic section uses population labels like DanubeTisza_early throughout, but there's no straightforward way to link those to the individuals included.

**We have now added all population labels used and the corresponding individual IDs as defined in AADR (Mallick and Reich, 2023) to the Population definitions sheet in Additional file 2 to ensure proper linking of populations to published individuals.*

Genetic shifts

This section describes multiple analyses providing evidence for a genetic shift between MMP and MEM samples. The distinction is already quite clearly visible in the projected PCA (Fig. S14), where the MMP samples form a cline towards the eastern Mediterranean, whereas most of the MEM diversity falls within NE Europe/Baltic region. The haplotype-based analyses using chromopainter/fine-structure provide a more fine-grained resolution, clearly demonstrating high diversity within MMP spanning N Europe to S Europe.

**We are happy to see that the reviewer finds our conclusion about a major genetic shift convincing.*

I am confused about figure S23, which is a fineSTRUCTURE PCA but contains only ancient samples, and from groups not described at all in other analyses (e.g. Upper_Paleolithic_Neolithic). This relates to my previous comments on the different datasets, please clarify whether this analysis was done using haplotype data and if so which individuals were included and how they were imputed/phased.

**We initially conducted this and many other analyses for purely technical reasons to check that the 1240k data set would result in similar results and hence our interpretations are robust to the choice of data set and method - which they appear to be. But we agree that this use of different data sets is a bit hard to follow. In light of the comment above and the request of Reviewer 1 to discuss all findings in the main text, we decided to remove this analysis in favor of having a more streamlined text to follow.*

Within the MEM group, structure is less pronounced but there does seem to be some difference in affinity to SE Europe/Balkans, seen in PCA, finestructure clustering and also in the MOSAIC analyses. It's not really discussed in the main text, but would be worth commenting on. From the chunklength PCA it appears to be the later samples of the MEM group, is this consistent with later gene flow from the Balkan region?

**This is an interesting observation. To follow up on this, we now present a new qpWave analysis on two groups of earlier and later MEM samples as based on ^14^C dates, which showed that none of the currently available reference population can break the cladality between these two groups and hence confirms they are rather homogenous.*

**Since the haplotype-based analyses identify three MEM samples that cluster more closely with modern Hungarians and Romanians than the remaining samples (see population dendrogram in Supplementary Fig. 23) and since two of those samples are among the three samples dating most recently, we also quickly checked whether there is any association between recency and cluster using a X2-test, but that was not significant (p = 0.12).*

**Based on these findings, our rather small sample size and the fact that the association of samples from the Balkans to cultural groups is rather difficult for that period, we do not think we can reach any robust conclusion on that question and would prefer not to pronounce ourselves either way.*

The relate analysis is interesting, but as the authors mention the difference observed is likely a consequence of the higher diversity of ancestry in the MMP group which would be expected to manifest itself in elevated Ne.

**We are happy to see that the review finds the relate analysis interesting and certainly agree that the simple split model underlying that analysis is too simple to reflect the full complexity of the relationship between these populations (as we now state explicitly).*

Relationships with other populations

This section provides a more in-depth examination of the relationships between MMP / MEM samples and other groups. Using qpAdm, the authors find that at model of >40% contribution of NE European populations is needed to fit MEM as descendants from MMP. While I don't have concerns about this result, the authors state in the supplement that "The only plausible qpAdm models that explain the genetic variability of MEM must comprise MMP as models without MMP and only other populations could be rejected (Dataset S4)". However this statement seems to be not supported in Dataset S4, as all models with MEM as target have MMP as one of the sources (Left population 1).

**We actually fully agree with the reviewer about the interpretation. The issue described arises because in Dataset S4 we only showed models that were not rejected due to the very large number of models tested. We have now amended this by adding all the qpAdm models tested, confirming that those were all rejected.*

**However, and as suggested by reviewers, we now added to this analysis additional populations (Olalde et al. 2023, Stolarek et al. 2023 and Antonio et al. 2024) that were not previously tested and also used the latest AADR version (v62.0) for other ancient populations. Some of these newer models do not include MMP samples but were still not rejected. We now discuss this in the main text.*

I also don't quite follow how a lack of ancient reference populations from NE Europe would lead to an overestimation of that ancestry.

**We originally found that the only models not rejected for MEM samples include at least two sources: a population from North-Eastern Europe, and MMP samples. The big question was whether MMP and a Norther-Eastern population were the true sources for MEM. It is important to stress here that any qpAdm analysis can only reject models, but never confirm them. So it is possible that there exist source populations much closer to MEM that could explain MEM without MMP or with a much smaller contribution of MMP. Whether such a population exists we do not know, but it appears likely, especially since the model that had the highest contribution of MMP was with Lithuania_Marvele_Roman, which is from a geographically rather distant region. The true source for the MEM samples must therefore have been a population that shared some ancestry with Lithuania_Marvele_Roman, but not Lithuania_Marvele_Roman itself (which is not surprising given their temporal distance).*

**To illustrate that, we now expanded the search for the true source by including recently published reference populations and found, for instance, that Poland_EMA and Russia_Krasnoe_Medieval can also be modeled using MMP (30.6% and 20.7% respectively) and Estonia_IA or Russia_Ingria_IA, respectively. It thus seems very likely that the true source was a population of mixed ancestry itself that shared some ancestry with MMP that groups like Lithuania_Marvele_Roman, Estonia_IA and Russia_Ingria_IA did not, for instance due to demographic events that occurred during antiquity. This interpretation is further supported by our finding that even further eastern samples (e.g. from the Olga-Oka region) can also be explained with MMP and North-Eastern European sources.*

**We not explain our reasoning regarding the lack of source populations in much more detail in the main text.*

For the MOSAIC analyses of modern Slavic-speaking populations, why were modern Slavs also included as sources in Fig. S25?

**The MOSAIC approach is not using any source population when inferring admixture proportions but does so uniquely on the inferred chunks. The so inferred sources are then compared to other populations solely for illustration - it was only at this stage that the modern Slavic-speaking populations were used.*

General

My only other general concern is the lack of inclusion of the samples from Stolarek et al in the 1240K analyses. Given the number of individuals and close geographic location of origin, their inclusion with the newly presented data would provide valuable context to the broader regional impact of the Slavic expansions.

**We agree that this omission was unfortunate and have now added the Storalek et al. 2023 dataset in several 1240K analyses. While this inclusion did not alter our interpretation (results fitted with our interpretation), it proved helpful both in providing more context to our results as well as in clarifying several points to various reviewers.*

Minor comments

The reference panel used for imputation should be mentioned. From the file names it looks like 1000G but not explicitly stated

**We indeed used 1000G, which we now state explicitly.*

Figure 2A is missing a legend for the color scale

**We have added a legend to this figure, thanks for spotting this.*

The symbols for the modern samples in Fig 2B are very small

**We have replotted this figure and improved the readability.*

In fig S10 it is very hard to distinguish samples due to small symbols and/or similar colors.

**The size of symbols was increased.*

Similarly, figs S11-13 would benefit from larger labels and less white space. Using the same colors for the new samples as in the main figures would also be helpful

**This was added and the colours were changed.*

In Fig S14 the legend seems to be switched for the LIB7,11 and other MMP samples. Labels for the individuals of the new samples would also be helpful

**This was adjusted and the figure was moved to the main text.*

Figs S16/S17 show outgroup f3, but the scale seems to be wrong? I.e. the f3 value for the highest sharing population discussed (e.g. Lithuania_BA for MEM) is the lowest on the axis

**We thank the reviewer for spotting the error in plotting. We have fixed this and added several published reference populations to the tests. The results of outgroup-f3 statistics are now updated in Additional file 2 and the supplementary figures were replotted. The results still support the similarity of MMP to Southern European and Lombard period populations, however, also demonstrate the low power of this analysis as reflected in large standard errors of f3 values. The similarity of both MMP and MEM to various small “Viking” groups likely reflects the diversity of these complexly defined groups and the results may also be distorted by low sample sizes (e.g. Poland_Viking which has 8 individuals).*

In the analyses of Avar ancestry, POH_Avar samples are defined as "outlying ancestry" in PCA, does that refer to the projected PCA? As mentioned above, labels would be helpful there.

**We have now described in full detail on which analysis this division was based on. The supplementary text 3.3 now reads: “****POH_Avar, contains individuals with an outlying ancestry on Chunklength PCA (Fig. 2A) and Projected PCA (Fig. 2B), Asian admixture component in ADMIXTURE (Supplementary Fig. 14) or forming a separate group on the fineSTRUCTURE population dendrogram (POH3+POH27+POH39). POH_N contains the rest of the individuals (POH11+POH13+POH28+POH36+POH40+POH41+POH44).”***

**Second round of review**

**Reviewer 1**

I thank the Authors for they kind replies and explanations. I think that the manuscript has really improved and the results are valuable and supported. It is basically ready for publication in this current form, with possibly some minor changes described below.

As for my previous question related to GLOBETROTTER/SOURCEFIND, I agree with the Authors’ response. My request was prompted by the chromopainter/fineSTRUCTURE analyses that the Authors’ already performed, but if they need to re-paint their samples, I agree that it is not worth doing it and MOSAIC is more than sufficient and perfectly reliable.

As for the MMP contribution to MEM groups (lines 23-25 page 10 and lines 1-8 page 11), I do not completely have the same point of view of Authors. From their Supplementary Table (Additional file 2), I could see at least 15 significant model including MMP. Although I can agree with their explanation that the true source population is lacking and MMP contributed to that source rather than to MEM directly, it is still speculative. Also, in their reply to my comment about this in Revision 1, the Authors said that qpAdm cannot be used to prove a contribution, but they use it in this way to prove a major genetic influx from Northern/Eastern Europe a few lines before this paragraph. So, I would suggest to slightly rephrase this part to tone it down a little to take more into account the results regarding the MMP contribution. Similarly, in line 24 of page 12 I suggest making the statement about complete replacement more nuanced (even just adding the word “virtually” or a similar one before “excluded”). I am not saying that the proposed scenario about the lack of the real ancient source is wrong: on the contrary, I may agree with Authors. But in the absence of such data, this should be presented as an alternative explanation.

I have some minor additional points (mainly related to form or minor details than to content):
- Additional file 1, Screening Results: it seems to me that only 53 samples reported, but in the Results they are 54 (line 12 page 20).
- Main text, line 2 page 6 and line 10 page 11 (and possibly other cases that I did not spot): it should be specified that it was a Supervised Admixture.
- Lines 6-11, page 13: the Authors start talking about continuity and conclude talking about shift. I think to have understood what they mean, but maybe this paragraph could be rephrased a little to make the logical link between first and second part stronger.
- Line 6, page 14: why do the Author assume that the local remaining individuals were “few”?

**Reviewer 3**

All the major points I raised were addressed.

**Reviewer 4**

The authors have addressed my concerns adequately, I only have a few minor questions remaining:

Dataset The genomic data presented is of high quality, with genomic coverage ranging from 5.1X - 9.4X. I am a bit surprised though that the authors used bwa-mem for read alignment, instead of the normally used bwa-aln. What was the reason for this? Also, as bwa-mem performs local alignment which can lead to spurious mappings for due a smaller number of read bases aligning with the remainder masked out with soft clipping. How were those situations treated?

**We are happy to read that the reviewer judges our data as of high quality. We have been using bwa-mem successfully for quite a while in our pipeline and found it to perform desirably at lower computational costs. We are aware of some studies comparing bwa-mem vs bwa-aln that reported higher mapping rates with bwa-aln, but we failed to replicate these findings in the past once reads were compared after all our filters were applied (as opposed to just comparing raw counts). We are also aware that bwa-mem allows for soft-clipping, and while this may indeed lead to spurious mapping, it also allows for the identification of reads where adapter-trimming failed. To balance this trade-off, our ATLAS pipeline allows for two filters to avoid the issues of spurious mappings: The first is a soft-clip fraction filter which discarded all reads for which softclipping bases make up more that an allowed fraction of bases. The second is a strict filter that ignores all reads that have any soft-clipped bases (filterSoftClips). In this study, we applied this second, more stringent filter for all our analyses.*

I thank the authors for the clarification and providing the relevant detail in the methods now. I apreciate that the authors are stringent with respect to reads mapped with soft-clipped bases. A minor issue with this is that in our own experience bwa mem will also often soft clip terminal DNA damage substitutions, so I would expect a small coverage dropoff for the non-UDG libraries using this approach. However it shouldn’t affect the majority of the deep-sequenced data here

I was also a bit confused on the different datasets being used in different analyses. In section 4.2.1, the authors describe a set of reference samples that appear to have been processed in the same way as the newly generated samples, i.e. re-mapping from read data in fastq format. The full list in Dataset S5 contains a total of 215 reference samples processed in this way. From what I can gather, the only analyses carried out where those samples are included is the PCAngsd analysis presented in Supp Fig 11-13, but that does not include the Damgaard et al 2018 individuals which appear not be used at all. For analyses presented in 5.2 (Projected PCA), the authors use a different panel including also capture samples, on a pulldown of 1240K sites. Confusingly, in section 5.3 (ADMIXTURE) there is again a dataset description that includes a 1240K + HO panel generated by merging the AADR dataset. Presumably that the same merge that has been used for 5.2? Also, despite being described in 5.3, this 1240K + HO panel doesn't seem to have been used for any ADMIXTURE analyses, but mainly for the fstatistics. It would thus be very helpful if the authors could clarify/streamline those sections, and also include a table with metadata for the public data used in the analyses. *We apologize that our description of data sets used was not straightforward. We used three different datasets: 1) The first that allowed us to analyze the WGS samples using genotype likelihoods. 2) The second allowed us to include the PJP010 sample data that is 1240K captured. 3) The third was used for haplotype inference in chromopainter. This last data set differs from the former because chromosome painting requires a higher quality reference and because that reference was already preprocessed.

**We have now updated the text to make these uses much more clear in the methods section, which now also includes most parts previously presented in the supplementary information. To streamline the presentation, we also removed samples not relevant for the analysis. However, we did not include the same rich metadata for the large number of public data used - we feel this would be overwhelming. But we remain open to amend if the reviewer insists.*

The updated methods section does make the dataset generation clearer. I still have some more minor comments and a question related to the imputation.

1) The updated text is still somewhat inconsistent/unclear as to which samples have been included in the GL dataset generated with ATLAS. I understand that the core of this are the newly sequenced samples, but in the PCAngsd public ancient samples are still inlcuded. Those were previously listed in dataset S5, but have now seem to have been removed from that table.

2) For the imputation of the newly sequenced samples, the authors use as input the biallelic VCF generated by ATLAS (dataset 1 for downstream analyses in the new methods). Does that mean that only sites that were called bi-allelic in the newly sequenced samples are included in this VCF file? This relates somewhat to the above comment about what final set of samples was included in the GL dataset, as the diversity of the population sample would impact which sites were retained in the final VCF. Presumably the authors extracted GLs for all sites present in the GLIMPSE reference panel as input for the imputation, but would be good to clarify.

I am confused about figure S23, which is a fineSTRUCTURE PCA but contains only ancient samples, and from groups not described at all in other analyses (e.g. Upper_Paleolithic_Neolithic). This relates to my previous comments on the different datasets, please clarify whether this analysis was done using haplotype data and if so which individuals were included and how they were imputed/phased.

**We initially conducted this and many other analyses for purely technical reasons to check that the 1240k data set would result in similar results and hence our interpretations are robust to the choice of data set and method - which they appear to be. But we agree that this use of different data sets is a bit hard to follow. In light of the comment above and the request of Reviewer 1 to discuss all findings in the main text, we decided to remove this analysis in favor of having a more streamlined text to follow.*

Unless I have downloaded the wrong version the fineSTRUCTURE PCA is still in the supplement (Fig. S22).

**Authors Response**

**Point-by-point responses to the reviewers’ comments:**

Reviewer 1 (Minor revisions):

I thank the Authors for they kind replies and explanations. I think that the manuscript has really improved and the results are valuable and supported. It is basically ready for publication in this current form, with possibly some minor changes described below.

As for my previous question related to GLOBETROTTER/SOURCEFIND, I agree with the Authors’ response. My request was prompted by the chromopainter/fineSTRUCTURE analyses that the Authors’ already performed, but if they need to re-paint their samples, I agree that it is not worth doing it and MOSAIC is more than sufficient and perfectly reliable.

We are grateful to read that the reviewer is happy with the analysis we performed.

As for the MMP contribution to MEM groups (lines 23-25 page 10 and lines 1-8 page 11), I do not completely have the same point of view of Authors. From their Supplementary Table (Additional file 2), I could see at least 15 significant model including MMP. Although I can agree with their explanation that the true source population is lacking and MMP contributed to that source rather than to MEM directly, it is still speculative. Also, in their reply to my comment about this in Revision 1, the Authors said that qpAdm cannot be used to prove a contribution, but they use it in this way to prove a major genetic influx from Northern/Eastern Europe a few lines before this paragraph. So, I would suggest to slightly rephrase this part to tone it down a little to take more into account the results regarding the MMP contribution.

We have toned down the language, the new sentence now reads:

*“Consequently, the only qpAdm models for MEM not rejected (i.e. with p ≥ 0.05) involve Eastern European precursors (e.g. Russia_Ingria_IA, Poland_Roman, Lithuania_Marvele_Roman, Poland_Viking, Sweden_Viking, Russia_Viking, Norway_Viking, Estonia_IA, Additional File 1: Section 3.2), thus suggesting a substantial genetic influx into Moravia between the 5th and 7th century.”*

Similarly, in line 24 of page 12 I suggest making the statement about complete replacement more nuanced (even just adding the word “virtually” or a similar one before “excluded”). I am not saying that the proposed scenario about the lack of the real ancient source is wrong: on the contrary, I may agree with Authors. But in the absence of such data, this should be presented as an alternative explanation.

We have corrected the statement as the reviewer suggests, the sentence now reads:

*“Our estimates however, suggest that the contribution of the immigrating population was substantial and given the scarcity of data from relevant reference populations, we note that even a scenario involving* ***virtually*** *complete population replacement in this region cannot be ruled out, although this represents one of several possible explanations*.”

We also toned down another statement along those lines:

*“Consequently, the true contribution of MMP to MEM was likely much smaller than 51.8% and even a* ***potential*** *complete replacement cannot be fully excluded.”*

I have some minor additional points (mainly related to form or minor details than to content):

- Additional file 1, Screening Results: it seems to me that only 53 samples reported, but in the Results they are 54 (line 12 page 20).

The screening of the additional sample was performed in the different laboratory through a slightly different process and we thus reported those findings separately in PJP010 Results, Additional file 1.

- Main text, line 2 page 6 and line 10 page 11 (and possibly other cases that I did not spot): it should be specified that it was a Supervised Admixture.

We have specified all relevant mentions of supervised admixture analysis in the text.

- Lines 6-11, page 13: the Authors start talking about continuity and conclude talking about shift. I think to have understood what they mean, but maybe this paragraph could be rephrased a little to make the logical link between first and second part stronger.

We agree that the structure of the paragraph should emphasize the conclusions and distinguish between the continuity and discontinuity results more clearly. The edited paragraph now reads:

*“The Prague-Korchak culture is often directly connected to the spread of Slavic speakers (26, 27) and it is thus tempting to interpret our findings as evidence that the Slavic language was brought to Central Europe by immigrating people. It is important to note, however, that the language spoken by the early bearers of the Prague-Korchak culture remains unknown as the earliest written sources connected to the region date to the 9th century, with the exception of an animal bone from the 6th century incised with Germanic runes (31). Yet our data does support genetic continuity at the studied microregion from our earliest samples (PJP10 and LIB11) that dated to the 7th to 8th century up to our latest samples that dated to the 10th century, a continuity that is also apparent from the material culture (69). Given this observed continuity from the 7th century onward, it seems plausible to interpret the preceding genetic and cultural shift as the arrival of Slavic speakers in the region.”*

- Line 6, page 14: why do the Author assume that the local remaining individuals were “few”?

We have reviewed the sentence and indeed, the assumption that the remaining individuals were few does not fully correspond to e.g. qpAdm contribution of MMP in possible models. The sentence now reads: “…*we propose a major population shift in most localities, followed by the assimilation of the remaining individuals of the previously local population,...”*

Reviewer 3:

All the major points I raised were addressed.

We thank the reviewer for his evaluation and for the review process.

Reviewer 4:

The authors have addressed my concerns adequately, I only have a few minor questions remaining (see attached review file).

1) I thank the authors for the clarification and providing the relevant detail in the methods now. I appreciate that the authors are stringent with respect to reads mapped with soft-clipped bases. A minor issue with this is that in our own experience bwa mem will also often soft clip terminal DNA damage substitutions, so I would expect a small coverage dropoff for the non-UDG libraries using this approach. However it shouldn’t affect the majority of the deep-sequenced data here.

We are happy to read that the reviewer agrees that the procedure we took was rigorous and adequate.

2) The updated methods section does make the dataset generation clearer. I still have some more minor comments and a question related to the imputation. 1) The updated text is still somewhat inconsistent/unclear as to which samples have been included in the GL dataset generated with ATLAS. I understand that the core of this are the newly sequenced samples, but in the PCAngsd public ancient samples are still inlcuded. Those were previously listed in dataset S5, but have now seem to have been removed from that table. 2) For the imputation of the newly sequenced samples, the authors use as input the biallelic VCF generated by ATLAS (dataset 1 for downstream analyses in the new methods). Does that mean that only sites that were called bi-allelic in the newly sequenced samples are included in this VCF file? This relates somewhat to the above comment about what final set of samples was included in the GL dataset, as the diversity of the population sample would impact which sites were retained in the final VCF. Presumably the authors extracted GLs for all sites present in the GLIMPSE reference panel as input for the imputation, but would be good to clarify.

We apologize for this omission which we have tried to explain in our answer but we have now added back the former Dataset S5 that is now available as Additional File 1: WG Results. This data set was used for the GL data set and to identify bi-allelic sites. For imputation we then indeed used all sites present in the GLIMPSE reference panel, as the reviewer correctly speculated and we now specify in the Methods section.

3) Unless I have downloaded the wrong version the fineSTRUCTURE PCA is still in the supplement (Fig. S22).

Indeed - very sorry for this. We did now remove the figure from the supplementary file.
